# Supplementary material for: A scalable platform to discover antimicrobials of ribosomal origin
Source: Nat Commun. 2022 Oct 17;13:6135. doi: 10.1038/s41467-022-33890-w (PMC9576775; doi:10.1038/s41467-022-33890-w)
Supplement: Supplementary file 1 — Supplementary Information [file 41467_2022_33890_MOESM1_ESM.docx]

*Supplementary Information for:*

**A Scalable Platform to Discover Antimicrobials of Ribosomal Origin**

Richard S. Ayikpoe^1,6,†^, Chengyou Shi^2,6,†^, Alexander J. Battiste^1,6,†^, Sara M. Eslami^1,6^, Sangeetha Ramesh^3,6^, Max A. Simon^4,6^, Ian R. Bothwell^1,6^, Hyunji Lee^1,6^, Andrew J. Rice^1,6^, Hengqian Ren^2,6^, Qiqi Tian^5,6^, Lonnie A. Harris^1,6^, Raymond Sarksian^1,6^, Lingyang Zhu^7^, Autumn M. Frerk^1,6^, Timothy W. Precord^1,6^, Wilfred A. van der Donk^1,4,5,6,8*^, Douglas A. Mitchell^1,3,6*^, and Huimin Zhao^1,2,4,6*^

^1^Department of Chemistry, ^2^Department of Chemical and Biological Engineering, ^3^Department of Microbiology, ^4^Department of Bioengineering, ^5^Department of Biochemistry, ^6^Carl R. Woese Institute for Genomic Biology, ^7^School of Chemical Sciences NMR Laboratory, University of Illinois at Urbana-Champaign, Urbana, IL, 61801, USA; ^8^Howard Hughes Medical Institute

^†^These authors contributed equally.

*To whom correspondence should be addressed:

Phone: 1-217-244-5360; Fax: 1-217-244 8533; E-mail: [vddonk@illinois.edu](mailto:douglasm@illinois.edu)

Phone: 1-217-333-1345; Fax: 1-217-333-0508; E-mail: [douglasm@illinois.edu](mailto:douglasm@illinois.edu)

Phone: 1-217-333-2631; Fax: 1-217-333-5052; E-mail: [zhao5@illinois.edu](mailto:douglasm@illinois.edu)

**Table of Contents:**

[Supplementary Methods 4](#_heading=h.30j0zll)

[Supplementary Figures 7](#_heading=h.2et92p0)

[Supplementary Fig. 1 7](#_heading=h.tyjcwt)

[Supplementary Fig. 2 8](#_heading=h.3dy6vkm)

[Supplementary Fig. 3 9](#_heading=h.1t3h5sf)

[Supplementary Fig. 4 10](#_heading=h.4d34og8)

[Supplementary Fig. 5 11](#_heading=h.2s8eyo1)

[Supplementary Fig. 6 12](#_heading=h.17dp8vu)

[Supplementary Fig. 7 13](#_heading=h.3rdcrjn)

[Supplementary Fig. 8 14](#_heading=h.26in1rg)

[Supplementary Fig. 9 15](#_heading=h.1ksv4uv)

[Supplementary Fig. 10 16](#_heading=h.44sinio)

[Supplementary Fig. 11 17](#_heading=h.2xcytpi)

[Supplementary Fig. 12 18](#_heading=h.1ci93xb)

[Supplementary Fig. 13 19](#_heading=h.2jxsxqh)

[Supplementary Fig. 14 20](#_heading=h.z337ya)

[Supplementary Fig. 15 21](#_heading=h.lnxbz9)

[Supplementary Fig. 16 22](#_heading=h.35nkun2)

[Supplementary Fig. 17 23](#_heading=h.3j2qqm3)

[Supplementary Fig. 18 24](#_heading=h.1y810tw)

[Supplementary Fig. 19 25](#_heading=h.4i7ojhp)

[Supplementary Fig. 20 26](#_heading=h.3whwml4)

[Supplementary Fig. 21 27](#_heading=h.qsh70q)

[Supplementary Fig. 22 28](#_heading=h.2bn6wsx)

[Supplementary Fig. 23 29](#_heading=h.3as4poj)

[Supplementary Fig. 24 30](#_heading=h.1pxezwc)

[Supplementary Fig. 25 31](#_heading=h.49x2ik5)

[Supplementary Fig. 26 32](#_heading=h.2p2csry)

[Supplementary Fig. 27 33](#_heading=h.147n2zr)

[Supplementary Fig. 28 34](#_heading=h.3o7alnk)

[Supplementary Fig. 29 35](#_heading=h.23ckvvd)

[Supplementary Fig. 30 36](#_heading=h.ihv636)

[Supplementary Fig. 31 37](#_heading=h.32hioqz)

[Supplementary Fig. 32 38](#_heading=h.1hmsyys)

[Supplementary Fig. 33 39](#_heading=h.41mghml)

[Supplementary Fig. 34 40](#_heading=h.2grqrue)

[Supplementary Fig. 35 41](#_heading=h.vx1227)

[Supplementary Fig. 36 42](#_heading=h.3fwokq0)

[Supplementary Fig. 37 42](#_heading=h.1v1yuxt)

[Supplementary Fig. 38 43](#_heading=h.4f1mdlm)

[Supplementary Fig. 39 44](#_heading=h.2u6wntf)

[Supplementary Fig. 40 45](#_heading=h.19c6y18)

[Supplementary Fig. 41 46](#_heading=h.3tbugp1)

[Supplementary Fig. 42 47](#_heading=h.28h4qwu)

[Supplementary Fig. 43 48](#_heading=h.nmf14n)

[Supplementary Fig. 44 49](#_heading=h.37m2jsg)

[Supplementary Fig. 45 50](#_heading=h.1mrcu09)

[Supplementary Fig. 46 51](#_heading=h.46r0co2)

[Supplementary Fig. 47 52](#_heading=h.2lwamvv)

[Supplementary Fig. 48 53](#_heading=h.111kx3o)

[Supplementary Fig. 49 54](#_heading=h.3l18frh)

[Supplementary Fig. 50 55](#_heading=h.206ipza)

[Supplementary Fig. 51 56](#_heading=h.4k668n3)

[Supplementary Fig. 52 57](#_heading=h.2zbgiuw)

[Supplementary Fig. 53 58](#_heading=h.1egqt2p)

[Supplementary Fig. 54 59](#_heading=h.3ygebqi)

[Supplementary Fig. 55 60](#_heading=h.2dlolyb)

[Supplementary Fig. 56 62](#_heading=h.2dlolyb)

[Supplementary Fig. 57 63](#_heading=h.2dlolyb)

[Supplementary Fig. 58 64](#_heading=h.2dlolyb)

Supplementary Tables 65

[Supplementary Table 1 6](#_heading=h.2dlolyb)6

[Supplementary Table 2 6](#_heading=h.2dlolyb)7

[Supplementary Table 3 6](#_heading=h.2dlolyb)9

[Supplementary References 71](#_heading=h.1rvwp1q)

# Supplementary Methods

**Selection of RiPP BGC targets**

***Epipeptides.***  A total of three epipeptides were selected from three different phyla, Pseudomonadota, Cyanobacteriota, and Bacillota. The selection criteria were based on the novelty of core peptide sequence, phylogenetic diversity, and the presence of a radical SAM epimerase in the biosynthetic gene cluster (BGC).

***Glycocins*.** Glycocin clusters were mostly selected from a previous bioinformatic study of glycocins from Bacillota^1^. Each BGC encodes a distinct precursor peptide that does not group with any characterized glycocins. To uncover glycocins that originate beyond Bacillota, another putative glycocin BGC from Bacteroidota was also investigated in this study.

***Graspetides*.** Graspetide biosynthetic gene clusters were selected from a recently reported genome mining study of the graspetide RiPP class^2^. BGCs were prioritized based on predicted structural novelty of the putative graspetide product, i.e., graspetide groups with no/few characterized members were chosen. Specifically, BGCs were chosen from groups 2 (Gra-5), 7 (Gra-3), 8 (Gra-7), 10 (Gra-6), 13 (Gra-2, Gra-1), 18 (Gra-8), and an unnumbered cluster (Gra-4).

***Lanthipeptides*.** BGCs encoding putative lanthipeptides were selected from a recent report^3^ using the following criteria: 1) the placement of Ser/Thr/Cys residues were expected to yield novel ring patterns, 2) the BGCs were from bacterial taxa underrepresented in the lanthipeptide literature, and 3) the selected BGCs belong to large families of to-date uncharacterized lanthipeptides. A total of seven class I, fourteen class II, seven class III, and seven class IV lanthipeptide BGCs were selected (**Table S1**). These included: (a) several groups of class I lanthipeptide BGCs from Bacteroidota, Bacillota, and Actinomycetota that lack characterized members, (b) a group of class II lanthipeptides with several members of the heterogeneous II-2 group, which is unusual given its polyphyletic composition (i.e., Actinomycetota, Bacillota, Pseudomonadota, and Chloroflexota), (c) several groups of class III lanthipeptides from Bacillota, Pseudomonadota, Actinomycetota and Deinococcota, for which until recently^4^ no examples had been reported, and (d) several groups of class IV lanthipeptides from Actinomycetota and Pseudomonadota for which no characterized members have been reported.

***Lasso peptides*.** Lasso peptide BGCs were selected from previous bioinformatic surveys which used the leader peptidase as the marker^5^. BGCs were only considered if they contained a lasso cyclase, leader peptidase, RRE (fused to the protease or stand-alone), and a precursor with a RODEO score greater than 10. Uncomplicated clusters were selected based on core peptide novelty, phylogenetic diversity of the harboring organism, and the presence of possible immunity genes. Complicated lasso peptides were selected with the same criteria as well as the presence of putative secondary modification enzymes^6^.

***Linaridins*.** Linaridins were selected from a recent survey of all known linaridin BGCs. These were defined by encoding *linL* alongside *linE* and *linG*, or *linH*, which is a fusion of the latter two genes^7^. Targets were selected owing to the uniqueness of the precursor sequence, primarily the quantity and location of Cys and Thr. All predicted linaridins are confined to the Actinomycetota, therefore, taxonomical diversity was not used as a selection criterion.

***Linear azol(in)e-containing peptides (LAPs)*.** LAP BGCs harboring only core biosynthetic enzymes (YcaO cyclodehydratase, E1-like/F1-like protein and dehydrogenase) were selected from a comprehensive bioinformatic study of LAPs^8^. Each target belongs to a LAP group distinct from characterized representatives. Another six complicated LAP BGCs encoding additional dehydratases were also investigated in the study.

***Sactipeptides and ranthipeptides*.** Sactipeptide and ranthipeptide targets were selected from a previous bioinformatic study^9^. Simple examples of ranthipeptides and sactipeptides that consisted of only a precursor peptide and radical SAM enzyme were prioritized, owing to the expected difficulty in heterologous expression of the radical SAM enzyme.

***Thioamitides*.** Thioamitides were selected from a previous bioinformatic survey^10^. Given that TfuA-associated YcaO proteins modify non-RiPP substrates^10,11^, targets were chosen only if a high-confidence precursor peptide (e.g., a member of PF07862)^12^ was encoded directly adjacent to *ycaO* or *tfuA*. Further, comparison of similar BGCs from divergent organisms aided candidate prioritization and BGC boundary determination. Clusters encoding sulfur delivery machinery were also prioritized to maximize the chance of successful heterologous production.

***Thiopeptides*.** Thiopeptides were selected from a previous bioinformatic survey^13^. Selection criteria included: sequence uniqueness of the predicted precursor peptide and examples where the predicted biosynthetic proteins were fused. This latter criterion minimizes the number of genes needed for refactoring and was expected to increase the success probability.

# Discussion of DUF6229

A BLAST-P search using the precursor peptide of LanII as query returns a group of lanthipeptide precursors that are represented by PF19740.1 (DUF6229) (Supplementary Fig. 57A). The accession identifier WP_043693394.1 for the peptide from *Dyella* is linked in NCBI to the lysobactin BGC reported by Marahiel and coworkers (<https://www.ncbi.nlm.nih.gov/protein/WP_043693394.1>). However, the article describing the lysobactin BGC does not indicate that this peptide (nor other members of DUF6229) is involved in lysobactin biosynthesis^14^. Our data suggest these are lanthipeptide precursor peptides instead.

In addition to not being associated with lysobactin biosynthesis, we believe that assigning a single DUF6229 in an attempt to define this group of peptides is inappropriate, as we believe that they will produce unrelated products. The sequence deposited by Marahiel *et al.* for a contig that contains the lysobactin BGC encodes a peptide ([WP_036102482.1](https://www.ncbi.nlm.nih.gov/protein/WP_036102482.1/)) with a sequence that is highly similar in the leader peptide to that of the LanII precursor, but that has considerable differences in the core peptide (Supplementary Fig. 57B). The positions of Cys and Ser/Thr in the sequence are sufficiently different from that in the precursor of the LanII BGC that the final products will have different ring patterns and may have very different functions. Thus, collecting these sequences in one DUF/Pfam could be functionally misleading, as the similarity is driven by the leader peptide.

# Supplementary Figures


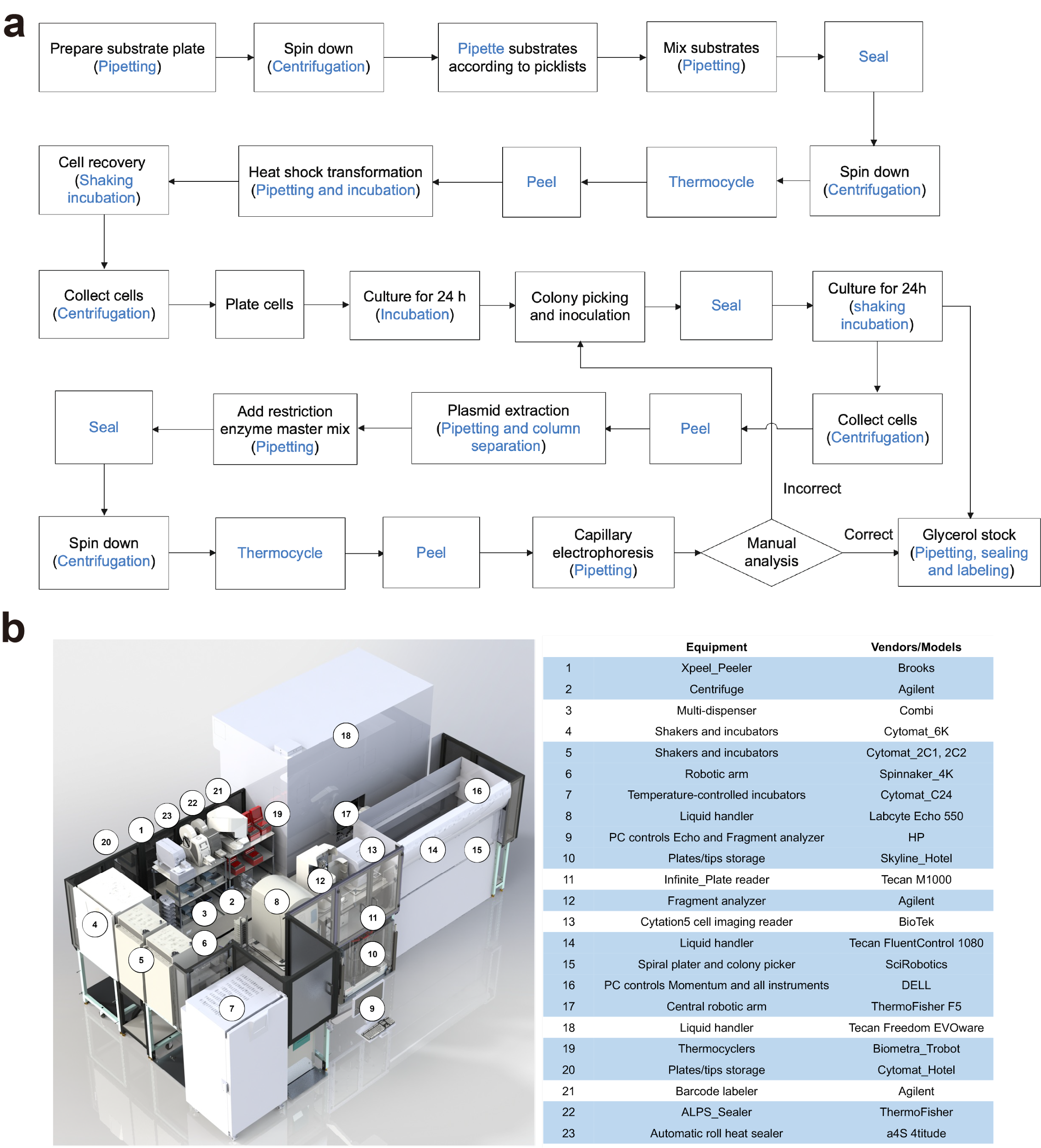


**Supplementary Fig. 1. Automated pathway refactoring using iBioFAB**. **a)** Process flow diagram for the build step. **b)** Equipment inside iBioFAB system (adapted from ref. 15 with permission from *Nature Communications*)^15^. Unit operations used in the automated pathway refactoring workflow and equipment involved are marked in blue in **a** and **b**.


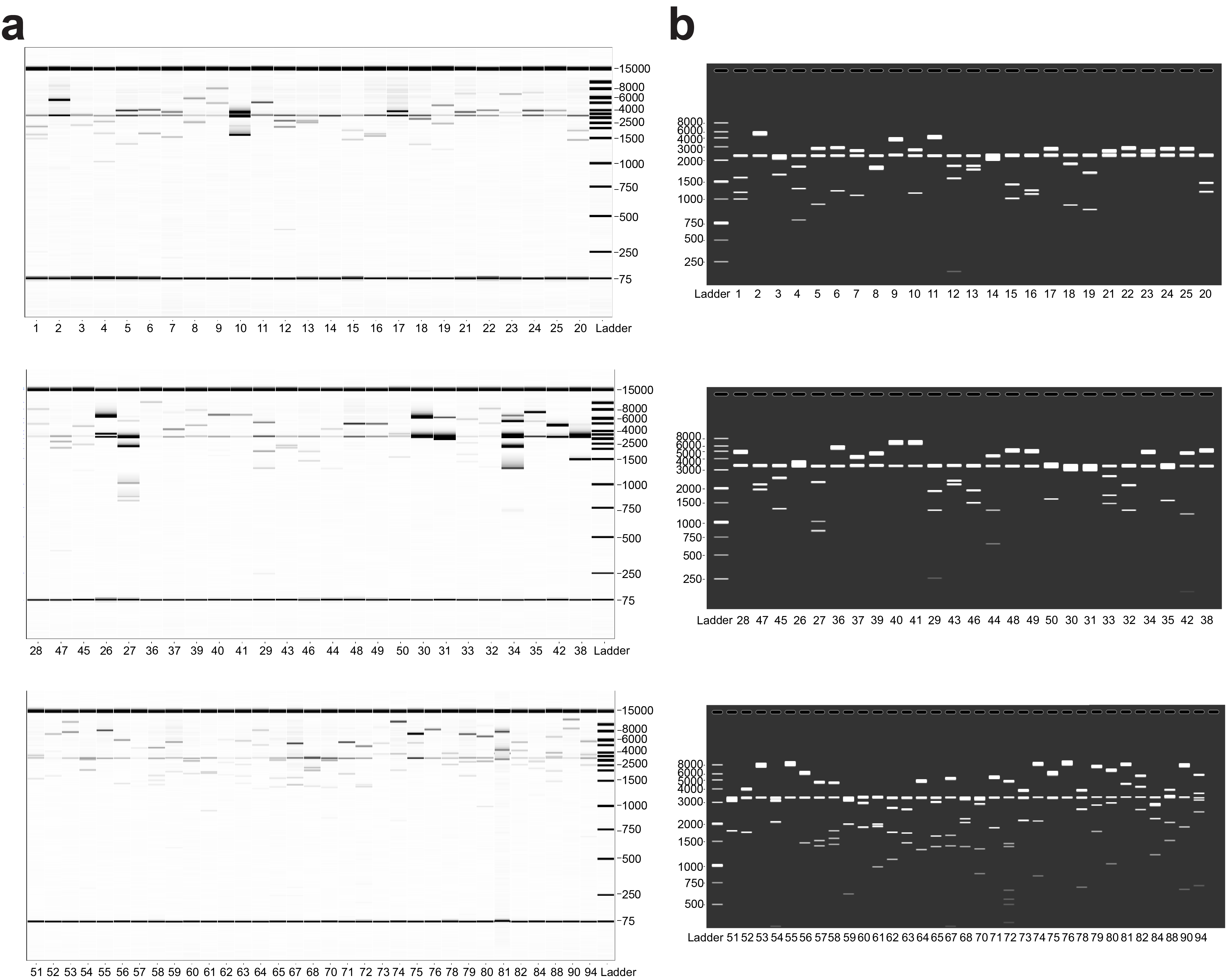


**Supplementary Fig. 2. Verification of 83 correctly assembled RiPP BGC expression constructs via *Asi*SI and *Mlu*I-HF digestion. a)** Capillary gel electrophoresis analysis. Sample numbering corresponds to the list of 83 RiPP BGCs in **Supplementary Table 1**. 75-15,000 bp ladder was shown. **b)** Simulated gel electrophoresis result using SnapGene. 250-8,000 bp ladder was shown. The automated construction of 83 plasmids and capillary gel electrophoresis analysis were performed only once.

**
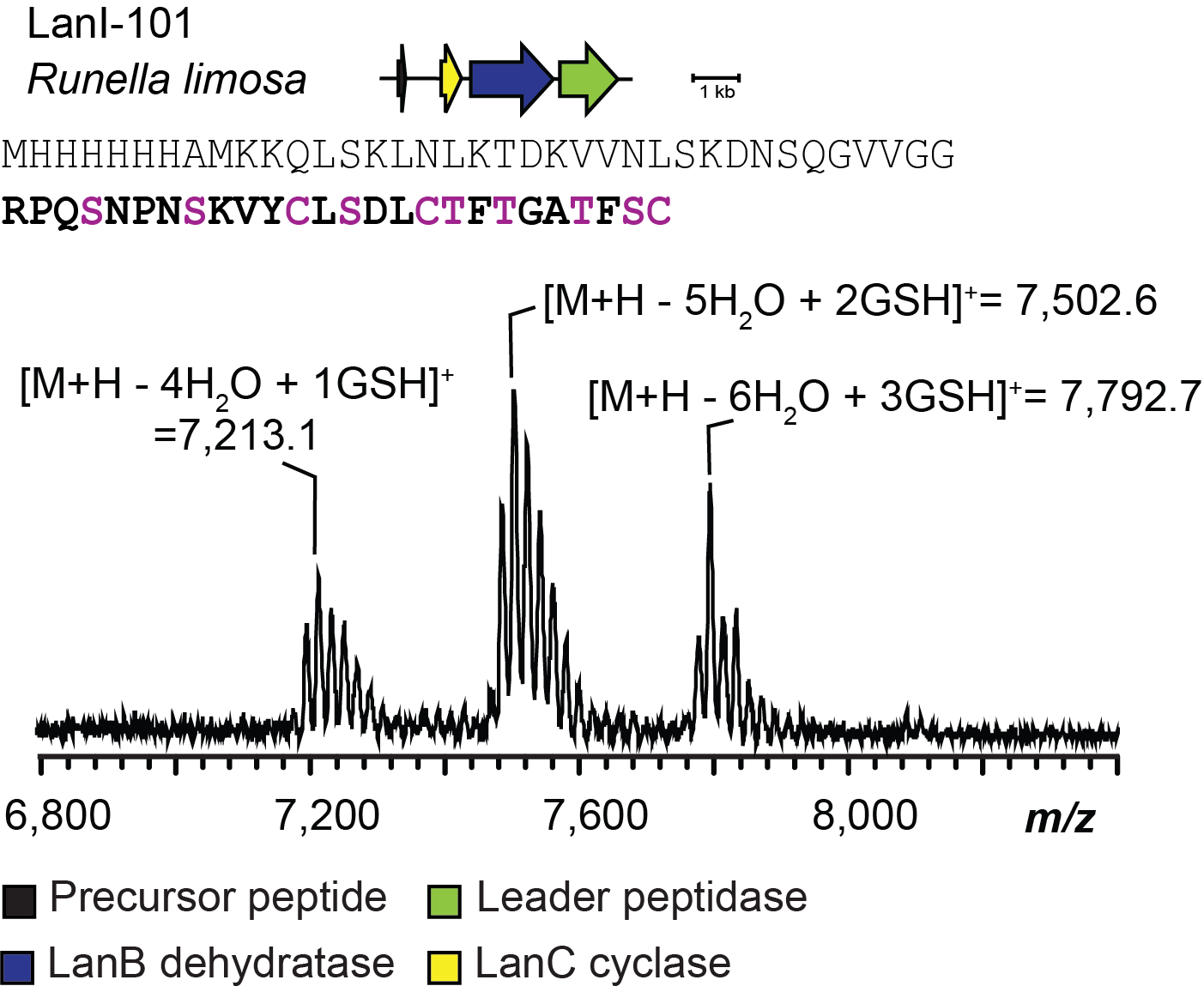
**

**Supplementary Fig. 3. Production of class I lanthipeptides.** MALDI-TOF mass spectrum of a class I lanthipeptide from the LanI-101 refactored BGC expressed in *E. coli*. Shown are the producing organism, gene diagram for the BGC, sequence of the precursor peptide with the predicted core peptide in bold font, and MALDI-TOF mass spectrum of the isolated peptide post Ni-NTA purification. Residues colored purple are possible sites of dehydration and/or cyclization. GSH, glutathione adduct (not disulfide). Calculated masses: 4-fold dehydrated + 1 GSH adduct [M+H] monoisotopic, *m/z* 7,212.5 calc., 7,213.1 obs., 5-fold dehydrated + 2 GSH adducts [M+H] monoiso., *m/z* 7,502.6 calc., 7,502.6 obs., 6-fold dehydrated + 3 GSH adducts [M+H] monoiso., *m/z* 7,792.7 calc., 7,792.7 obs.


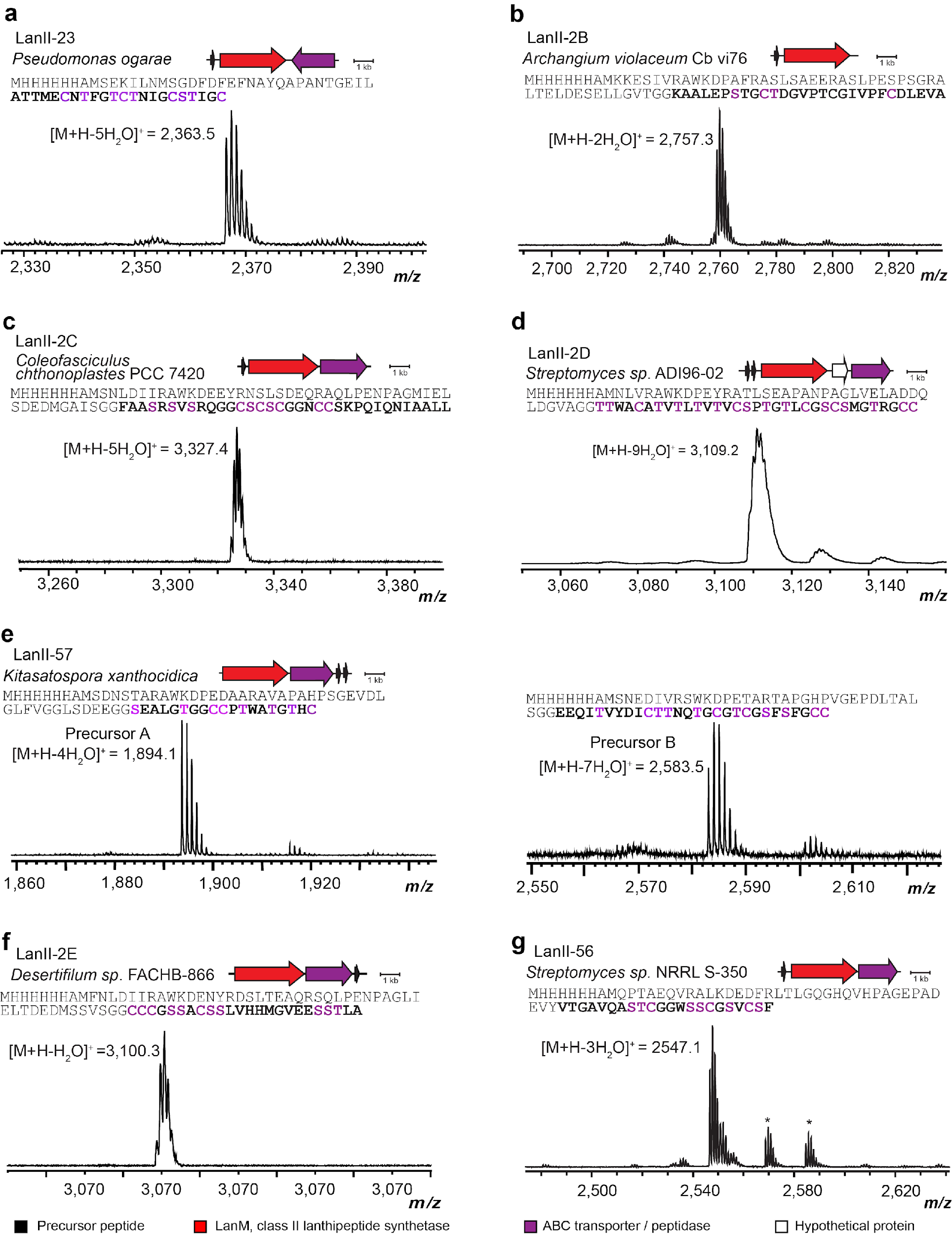


**Supplementary Fig. 4. Co-expression of class II lanthipeptides.** (previous page) MALDI-TOF mass spectra of purified class II lanthipeptides produced in *E. coli*. Shown are the producing organism, schematic BGC, sequence of the precursor peptide with the predicted core peptide bolded, and MALDI-TOF mass spectrum of the core peptide formed by digestion (endoproteinase AspN for panels f and g, endoproteinase GluC for panels a and e, and LahT150 for all other panels) of full-length peptide after HPLC purification. Calculated masses: panel a, 5-fold dehydrated [M+H] monoiso., *m/z* 2,363.9 calc., 2,363.5 obs., panel b, 2-fold dehydrated [M+H] monoiso., *m/z* 2,757.4 calc., 2,757.3 obs., panel c, 5-fold dehydrated [M+H] monoiso., *m/z* 3,327.9 calc., 3,327.4 obs., panel d, 9-fold dehydrated [M+H] monoiso., *m/z* 3,109.3 calc., 3,109.2 obs., panel e, right, 7-fold dehydrated [M+H] monoiso., *m/z* 2,883.1 calc., 2,583.5 obs., panel f, singly dehydrated [M+H] monoiso., *m/z* 3,100.3 calc., 3,100.3 obs., panel g, 3-fold dehydrated [M+H] monoiso., *m/z* 2,547.8 calc., 2,547.1 obs. Sodium and potassium adducts are indicated with asterisks.


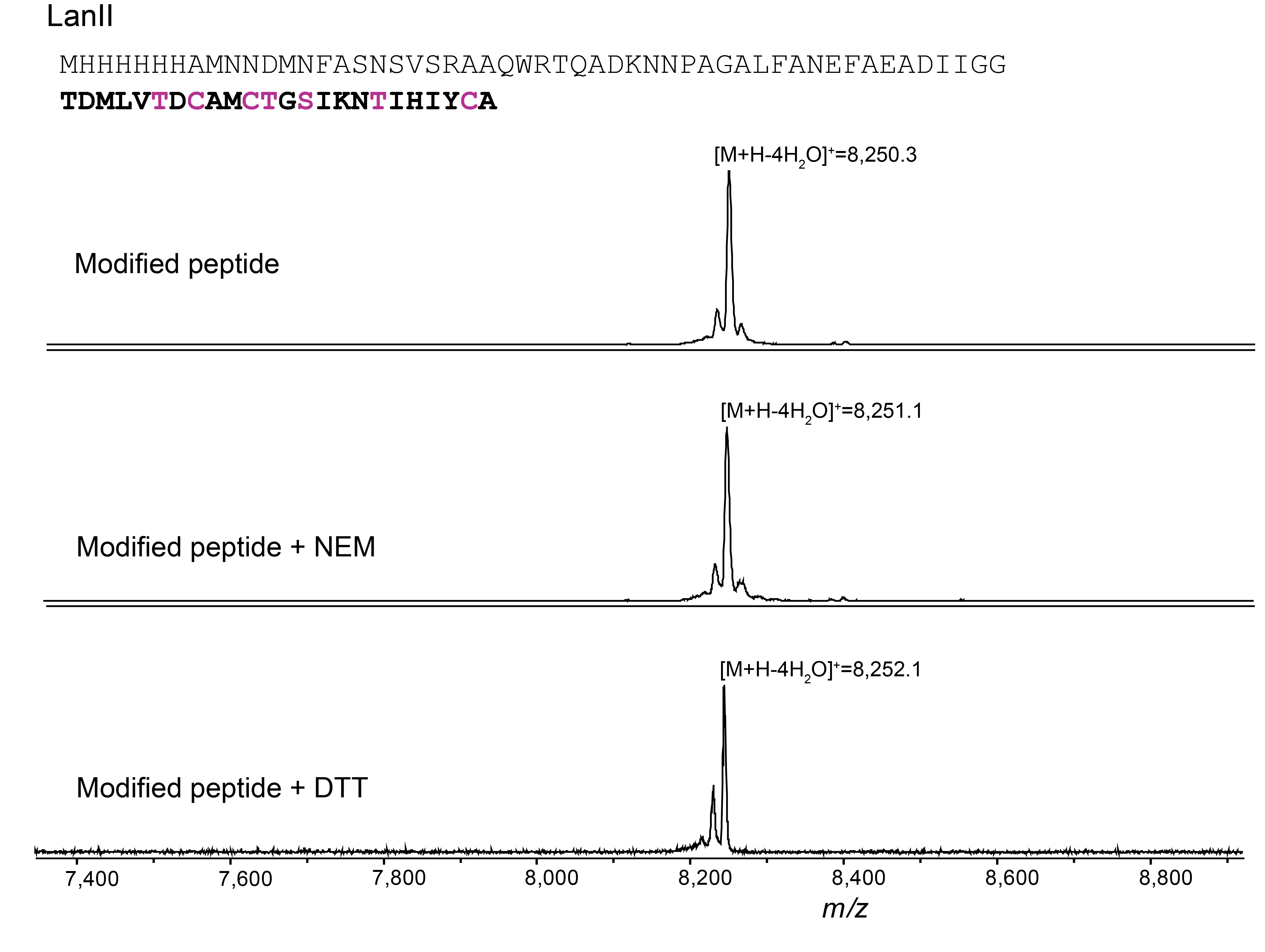


**Supplementary Fig. 5. Characterization of LanII peptide produced in *E. coli*.** MALDI-TOF mass spectra of NEM and DTT assays with LanII. The BGC is shown in **Fig. 5a**. Four-fold dehydrated [M+H] calc. average *m/z* 8257.3.


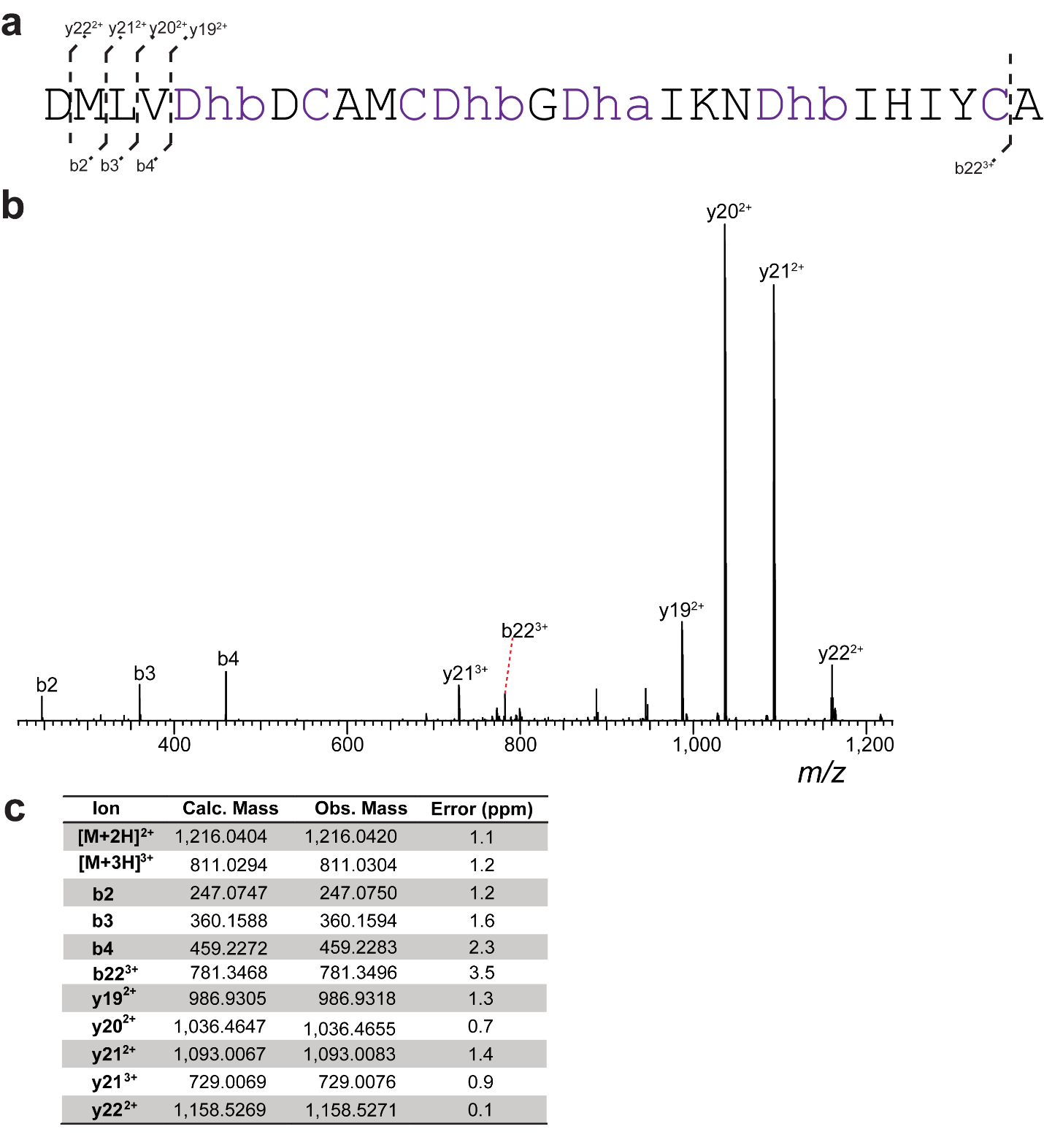


**Supplementary Fig. 6. HR-ESI tandem mass spectrometry analysis of LanII digested with endoproteinase AspN**. **a)** Core peptide sequence and observed MS/MS ions. **b)** Tandem mass spectrum. **c)** Observed and calculated masses for fragments.

**
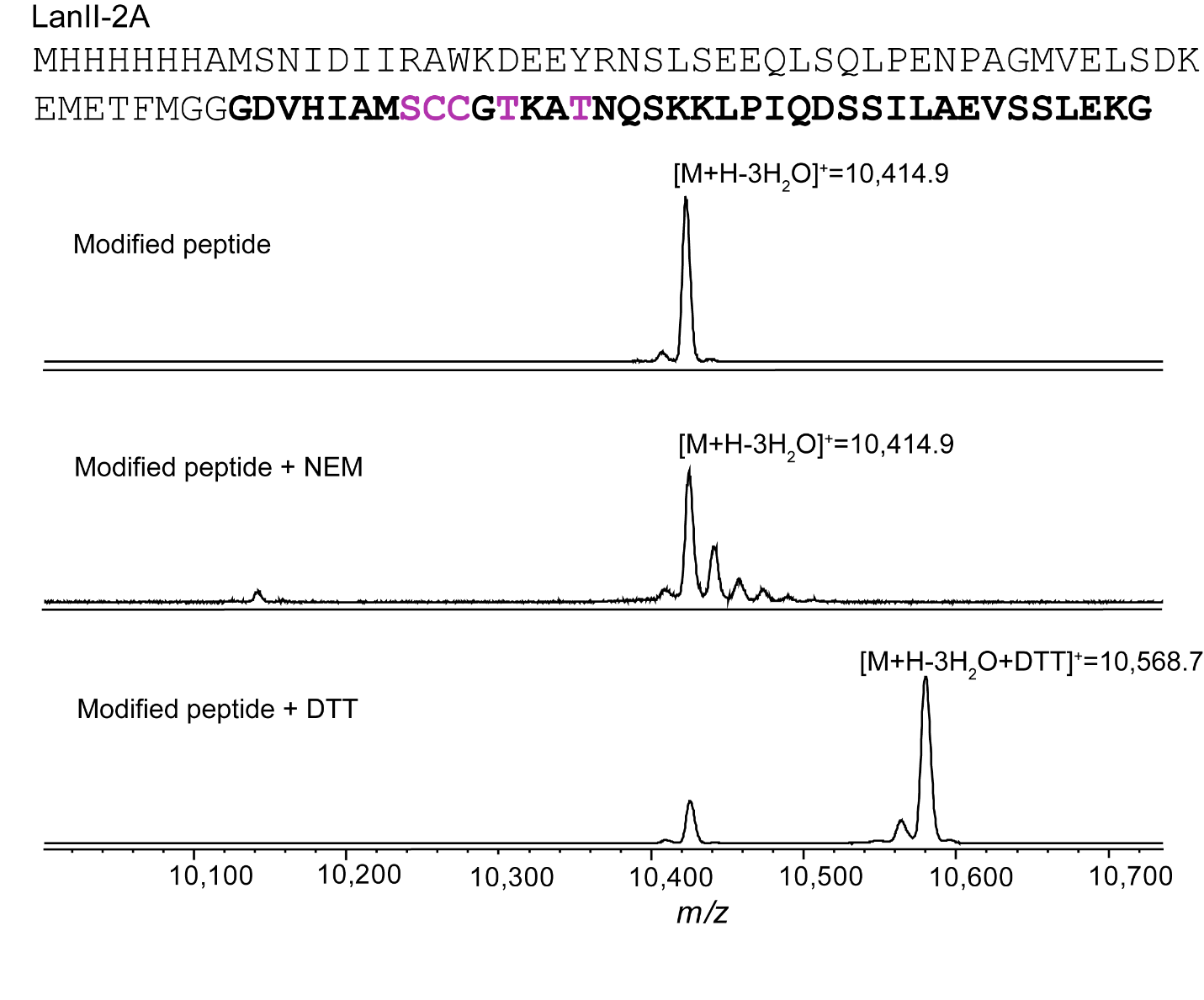
**

**Supplementary Fig. 7. Characterization of LanII-2A peptide produced in *E. coli***. MALDI-TOF mass spectra of NEM and DTT assays on LanII-2A. The BGC is shown in Fig. 5b.


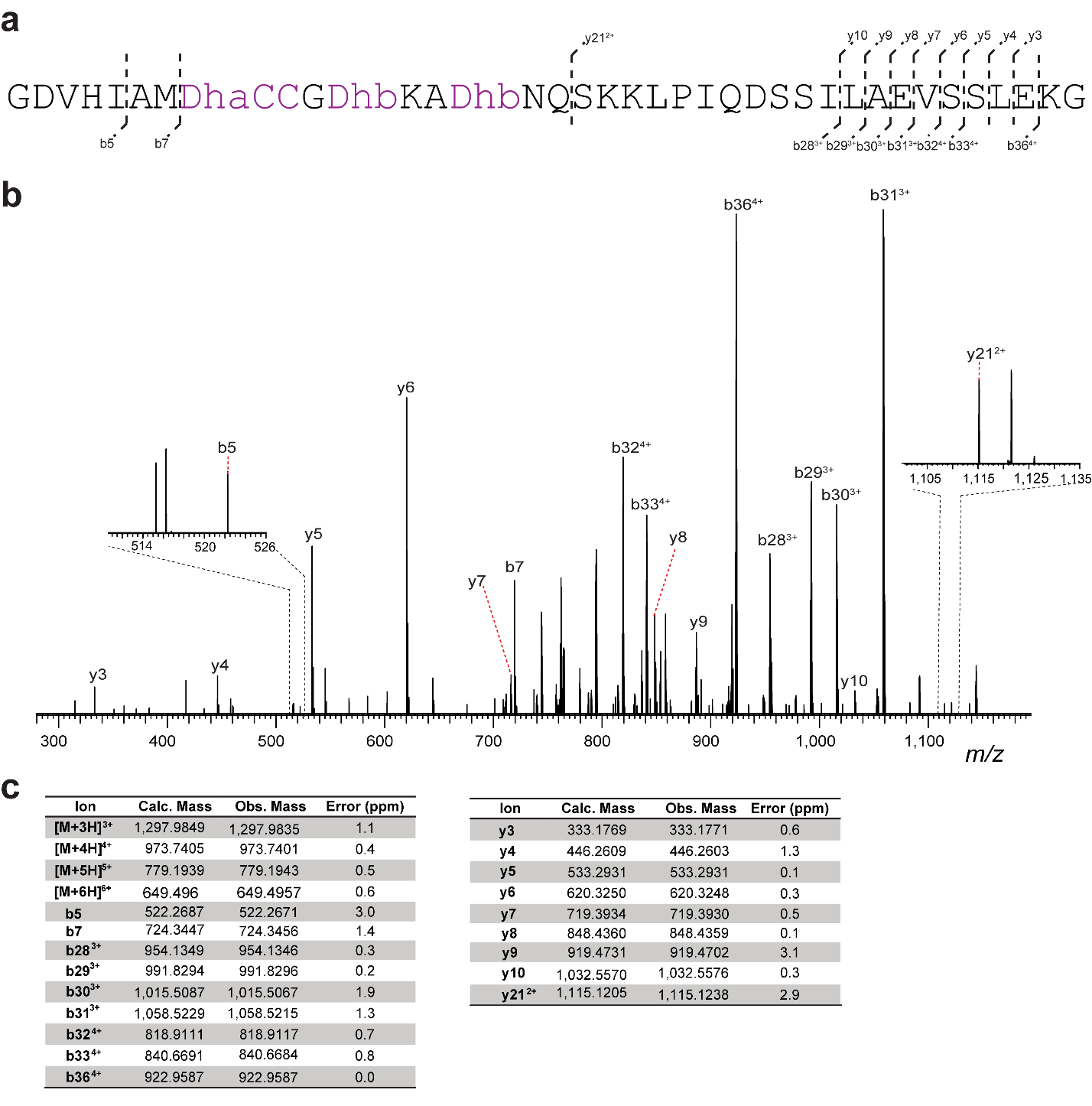


**Supplementary Fig. 8. HR-ESI tandem mass spectrometry analysis of LanII-2A after digestion of full-length product with LahT150**. **a)** Core peptide sequence and observed MS/MS ions. **b)** Tandem mass spectrum. **c)** Observed and calculated masses for fragments.

**
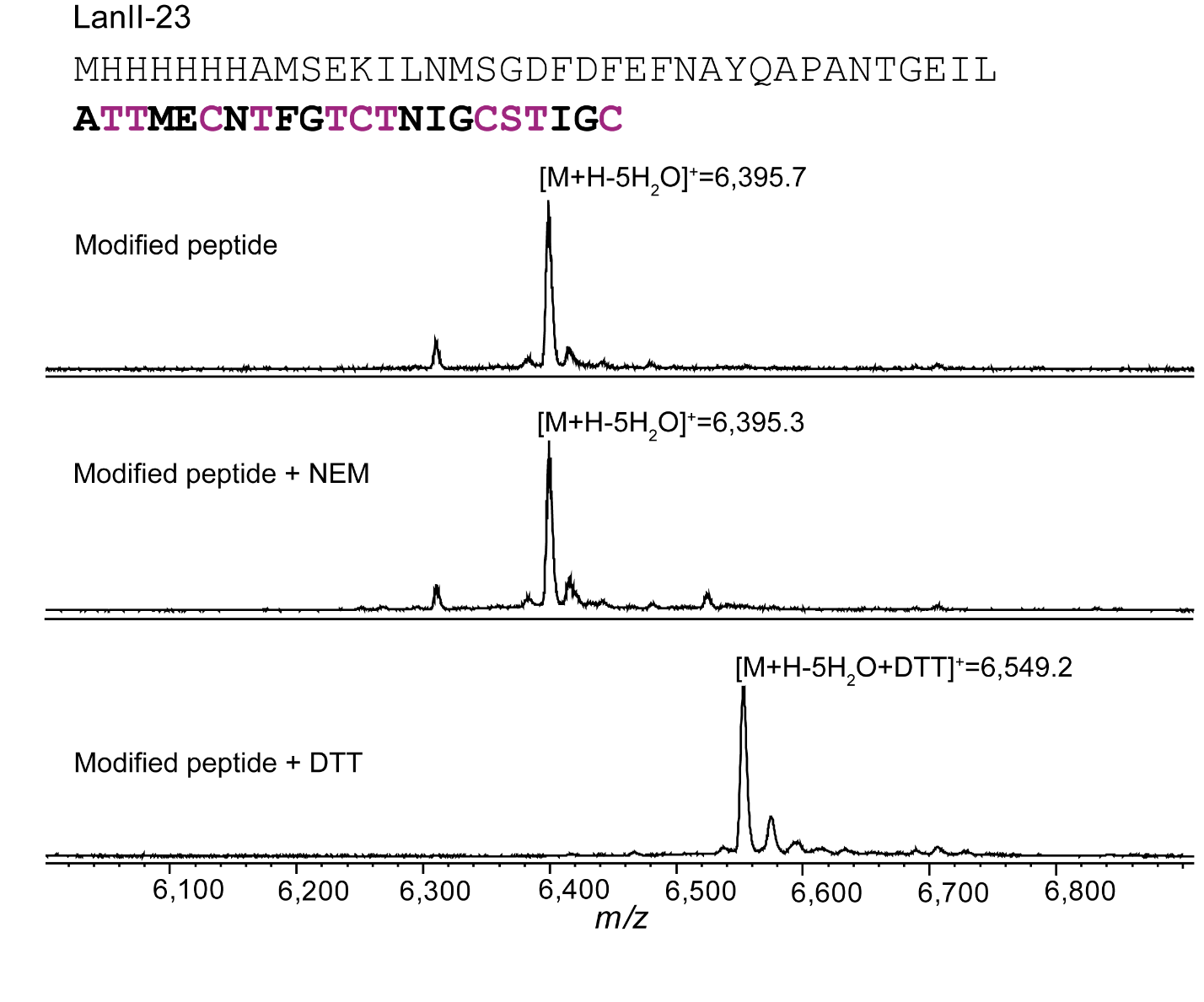
**

**Supplementary Fig. 9. Characterization of LanII-23 peptide produced in *E. coli.*** MALDI-TOF mass spectra of NEM and DTT assays with LanII-23.

**
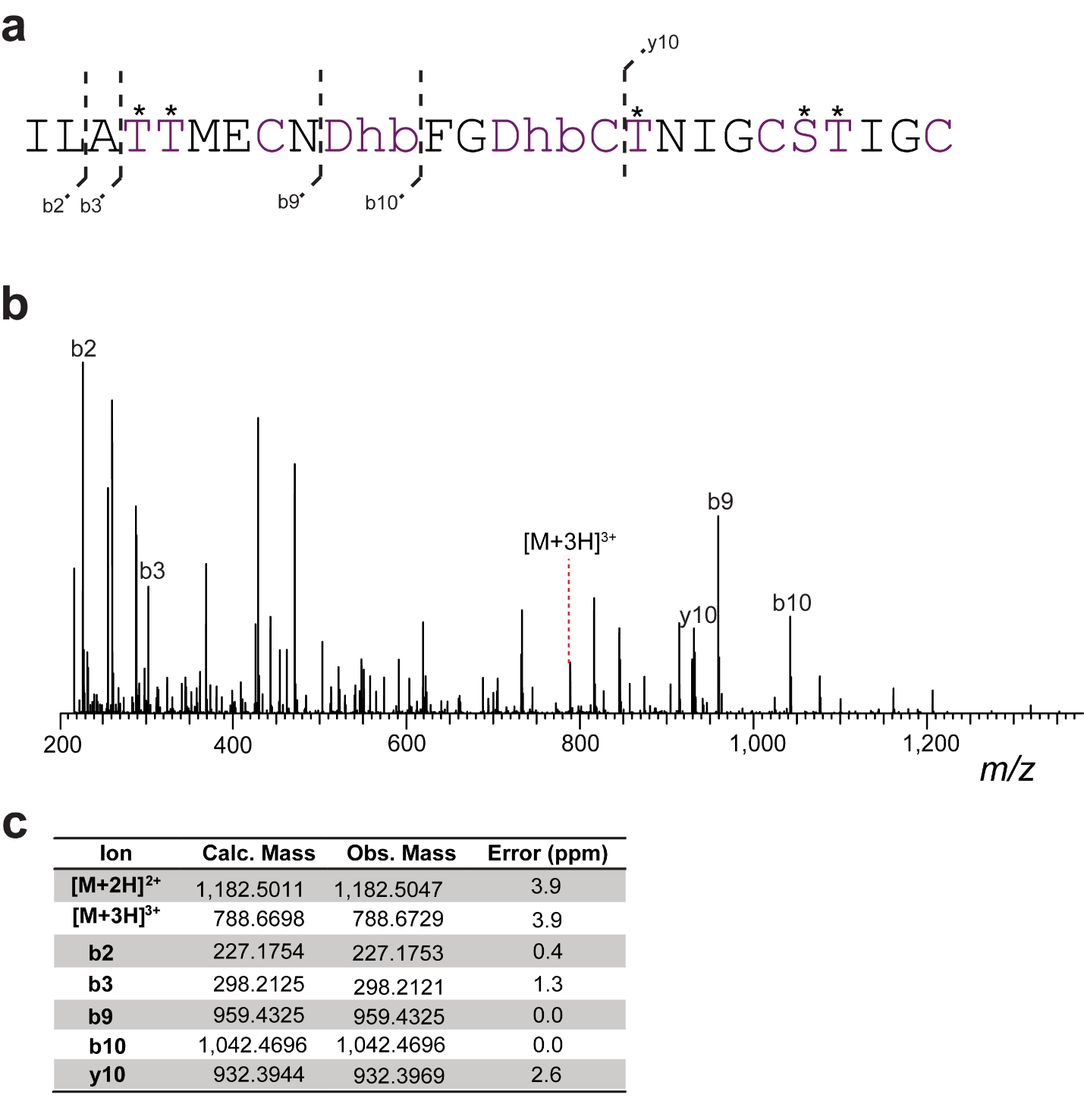
**

**Supplementary Fig. 10. HR-ESI tandem mass spectrometry analysis of LanII-23 after digestion with endoproteinase GluC**. **a)** Core peptide sequence and observed MS/MS ions. All possible dehydrated Ser and Thr residues are marked with asterisks (one of the two N-terminal Thr residues and two of the three C-terminal starred residues are dehydrated). **b)** Tandem mass spectrum. **c)** Observed and calculated masses for fragments.

**
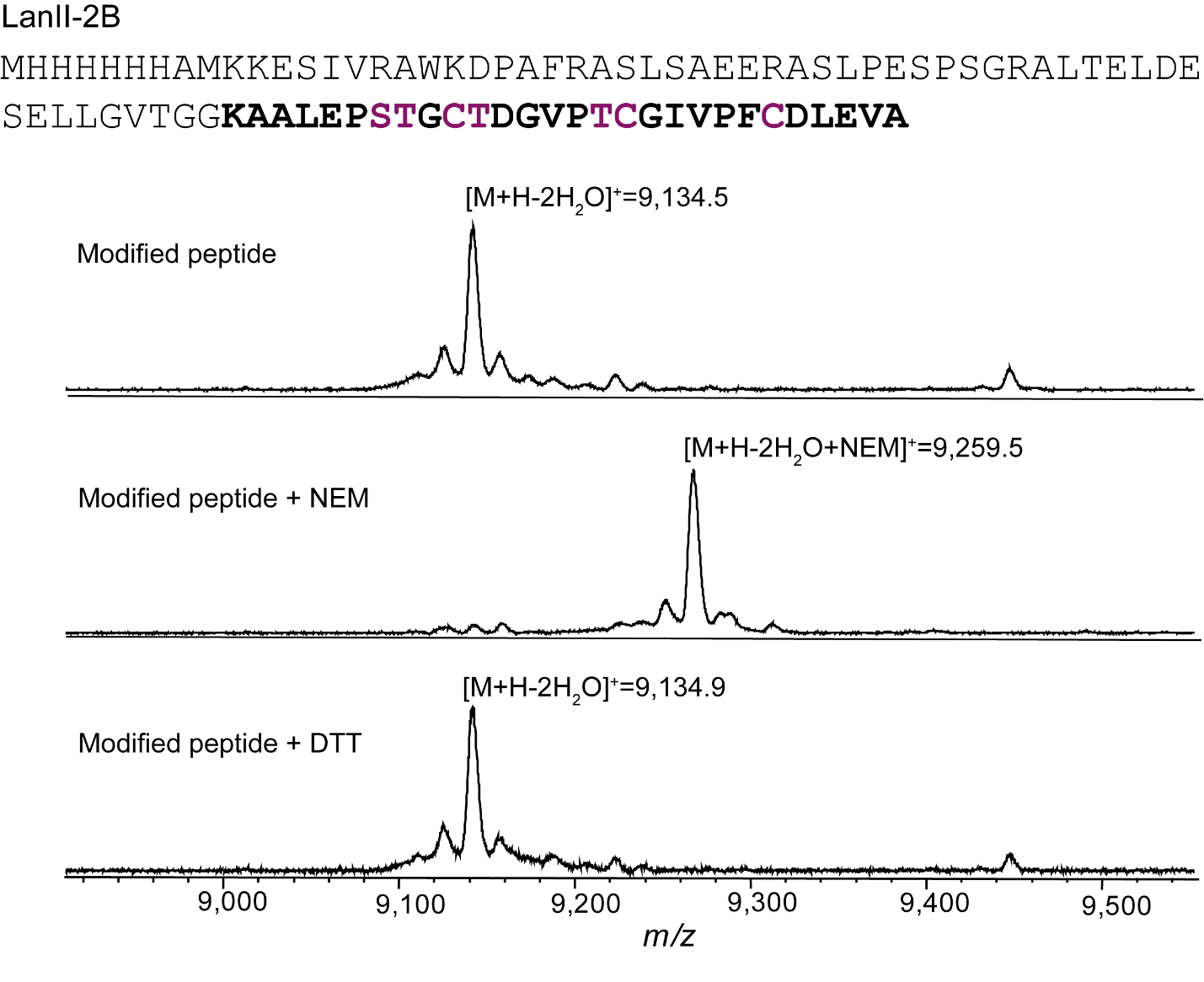
**

**Supplementary Fig. 11. Characterization of LanII-2B peptide produced in *E. coli.*** MALDI-TOF mass spectra of NEM and DTT assays with LanII-2B.

**
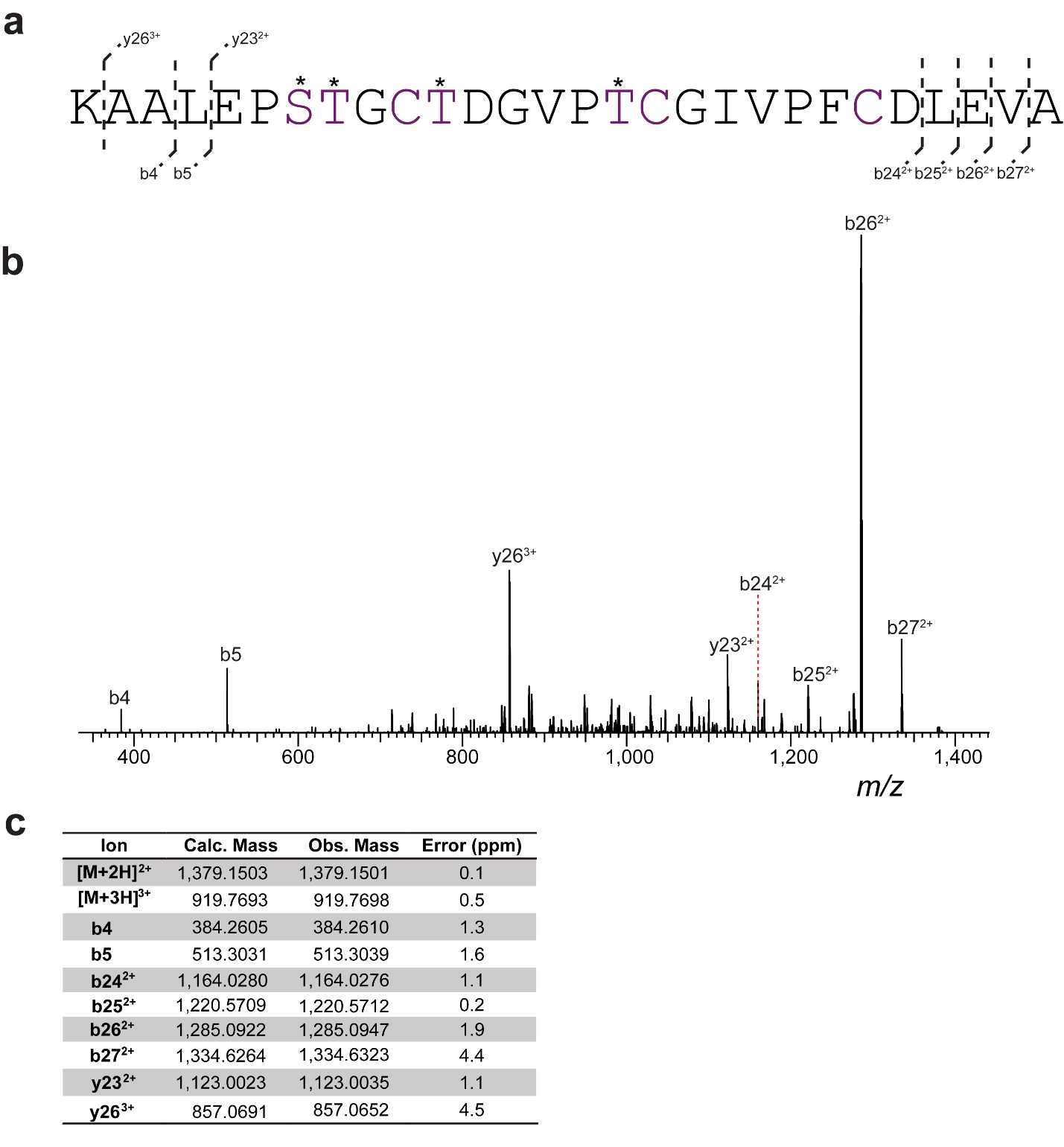
**

**Supplementary Fig. 12. HR-ESI tandem mass spectrometry analysis of LanII-2B produced by digestion of full-length product with LahT150**. **a)** Core peptide sequence and observed MS/MS ions. Possible dehydrated Ser and Thr residues are marked with asterisks (two of the four residues are dehydrated). **b)** Tandem mass spectrum. **c)** Observed and calculated masses for fragments.

**
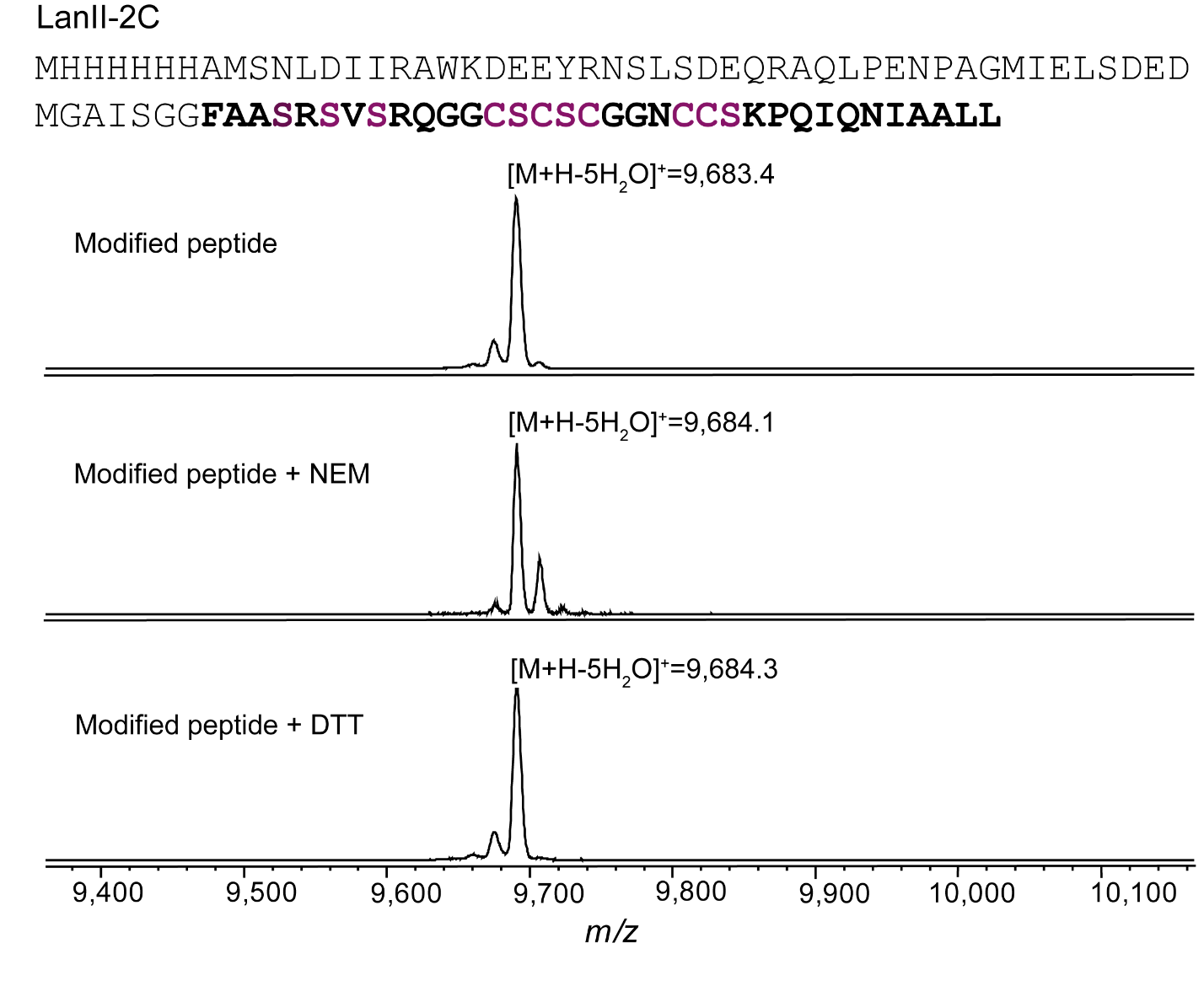
**

**Supplementary Fig. 13. Characterization of LanII-2C peptide produced in *E. coli.*** MALDI-TOF mass spectra of NEM and DTT reactions with LanII-2C.

**
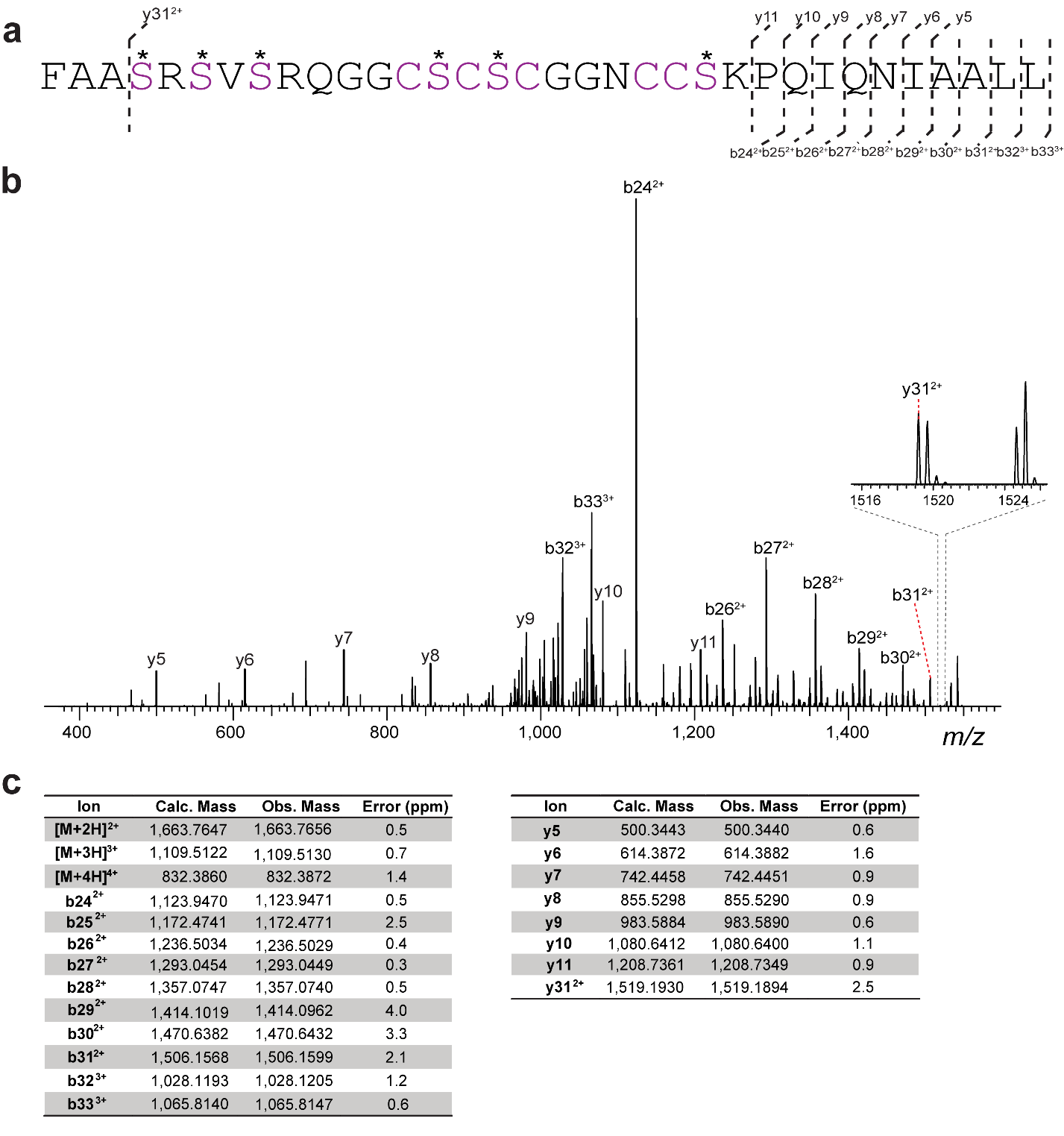
**

**Supplementary Fig. 14. HR-ESI tandem mass spectrometry analysis of LanII-2C after digestion of full-length product with LahT150**. **a)** Core peptide sequence and observed MS/MS ions. All possible dehydrated Ser residues are marked with asterisks (five of six residues are dehydrated). **b)** Tandem mass spectrum. **c)** Observed and calculated masses for fragments.

**
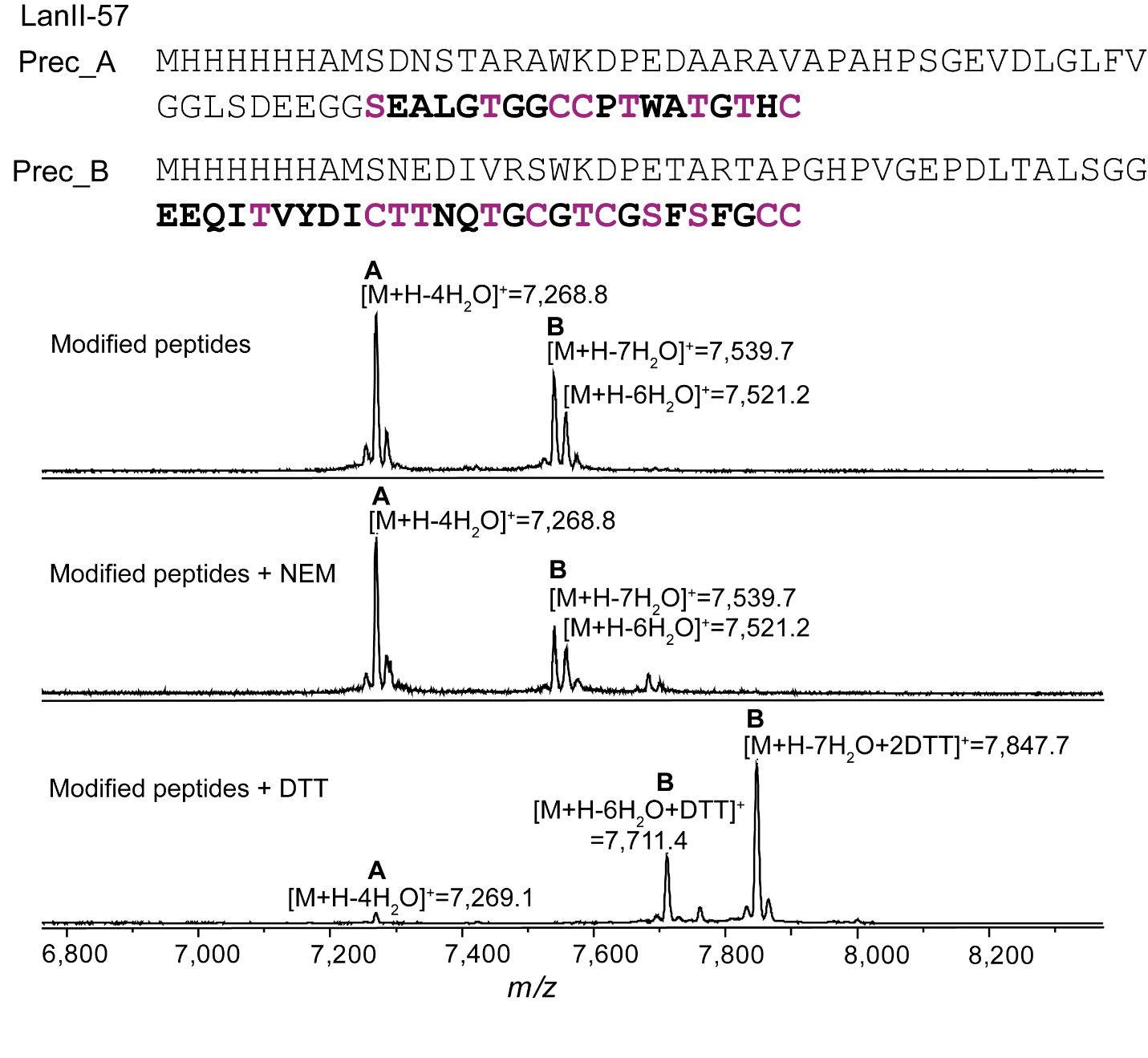
**

**Supplementary Fig. 15. Characterization of LanII-57 peptides produced in *E. coli.*** MALDI-TOF mass spectra of NEM and DTT assays with LanII-57 peptides A and B.


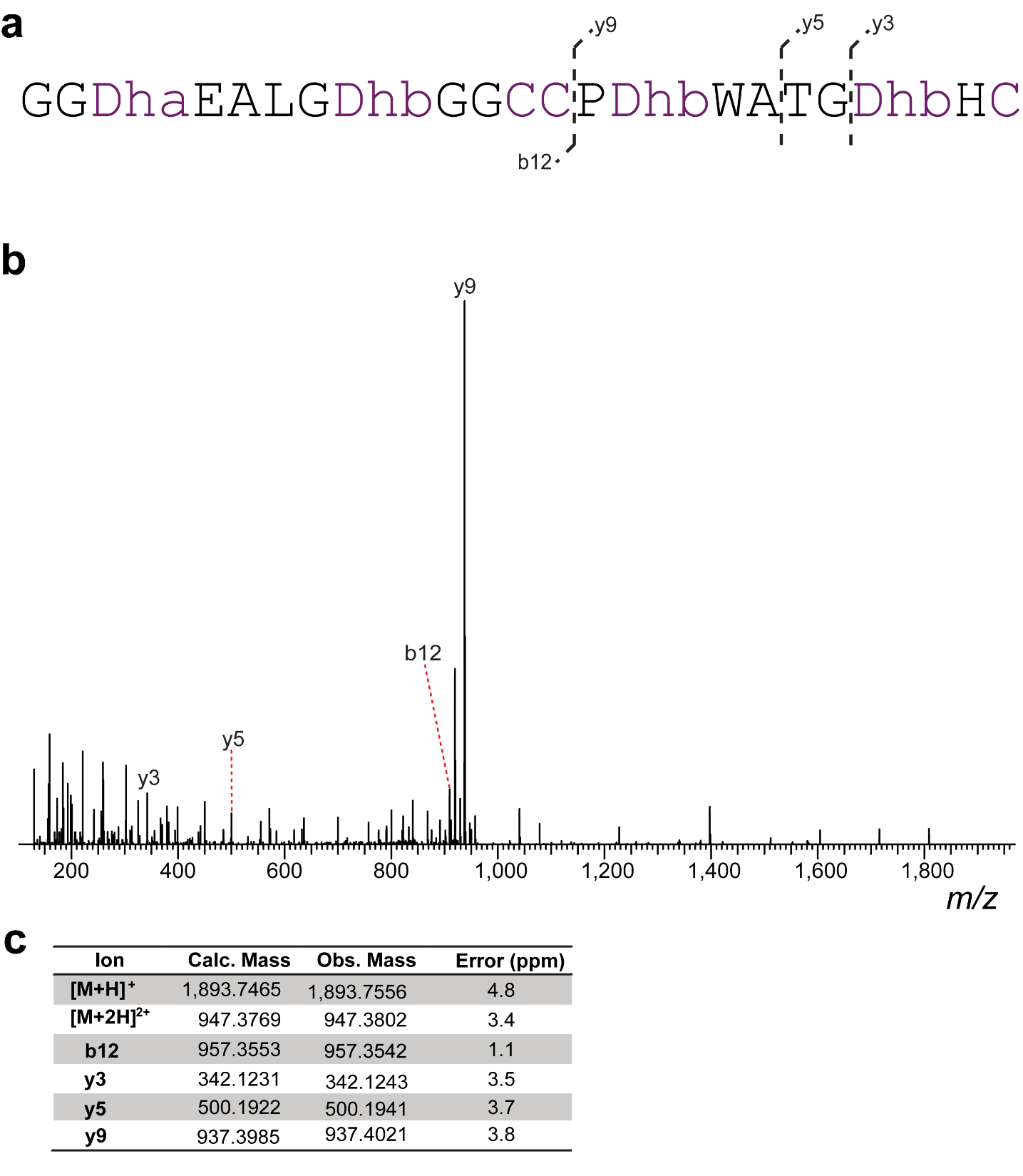


**Supplementary Fig. 16. HR-ESI tandem mass spectrometry analysis of LanII-57 peptide A digested with endoproteinase GluC**. **a)** Sequence of the C-terminal peptide after GluC treatment and observed MS/MS ions. **b)** Tandem mass spectrum. **c)** Observed and calculated masses for fragments.

**
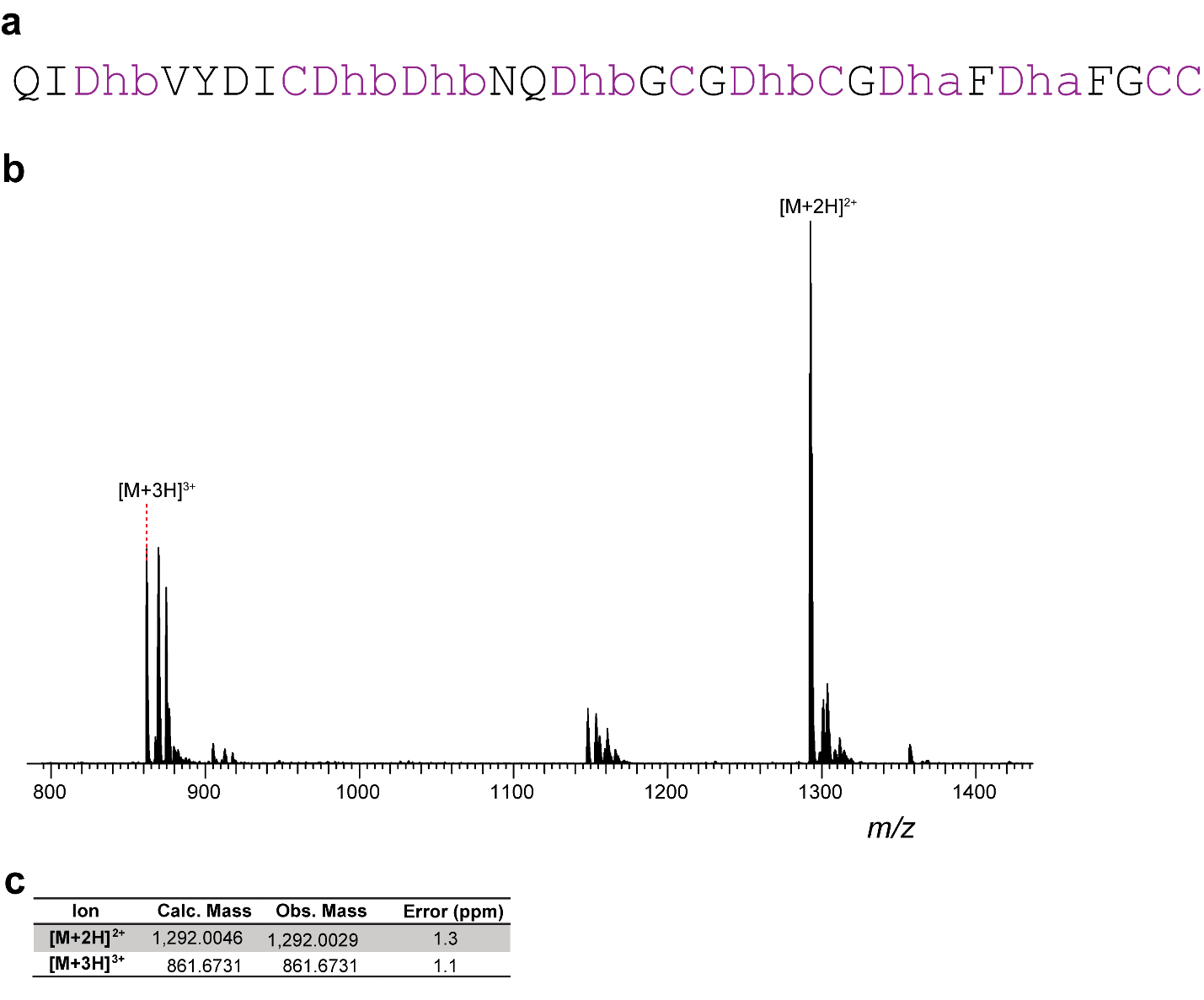
**

**Supplementary Fig. 17. HRMS analysis of LanII-57 peptide B digested with endoproteinase GluC**. **a)** Sequence of the C-terminal peptide after GluC treatment. **b)** Mass spectrum and observed mass ions. **c)** Observed and expected masses for the GluC digested and modified core peptide.

**
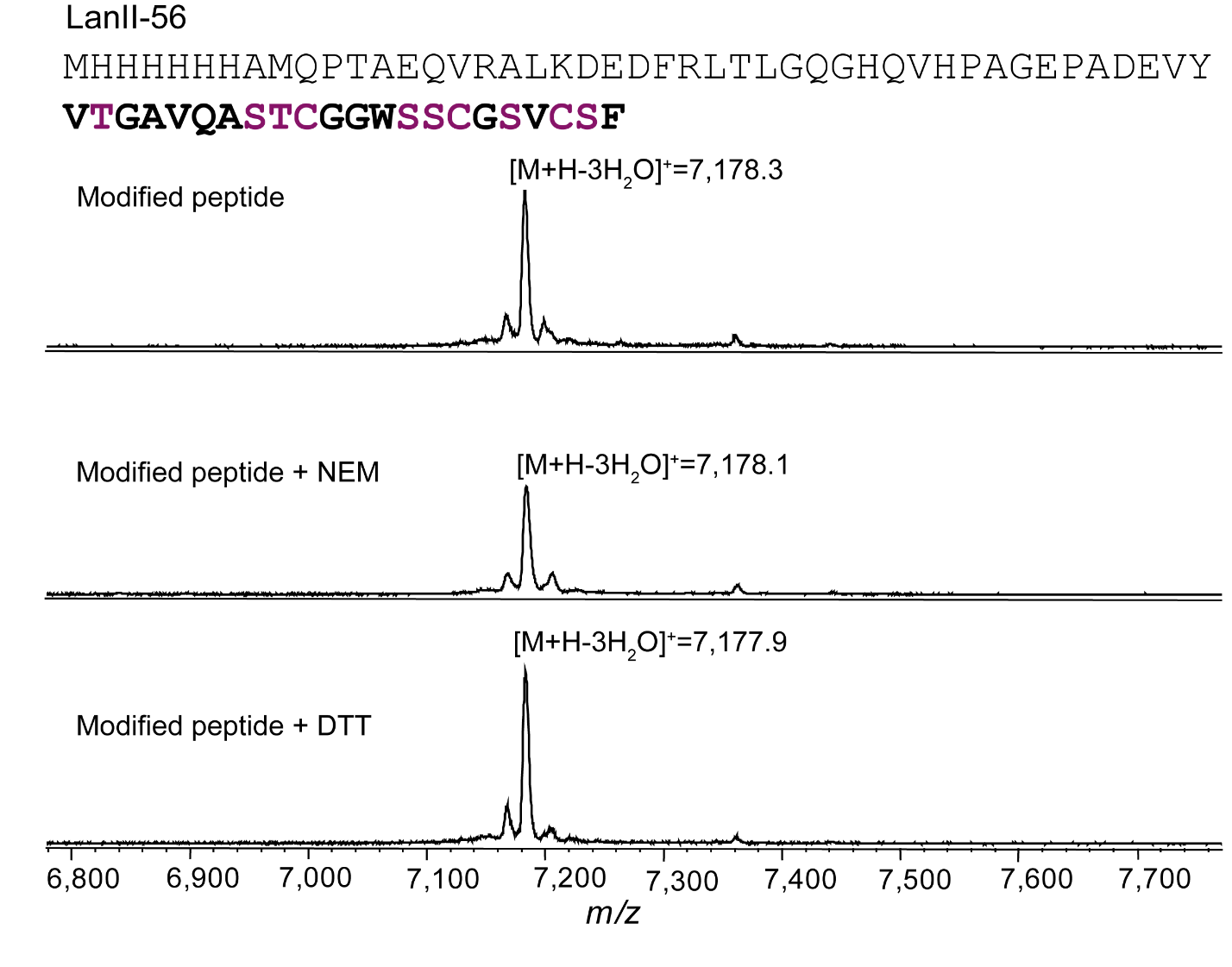
**

**Supplementary Fig. 18. Characterization of LanII-56 peptide produced in *E. coli.*** MALDI-TOF mass spectra of NEM and DTT assays with LanII-56.


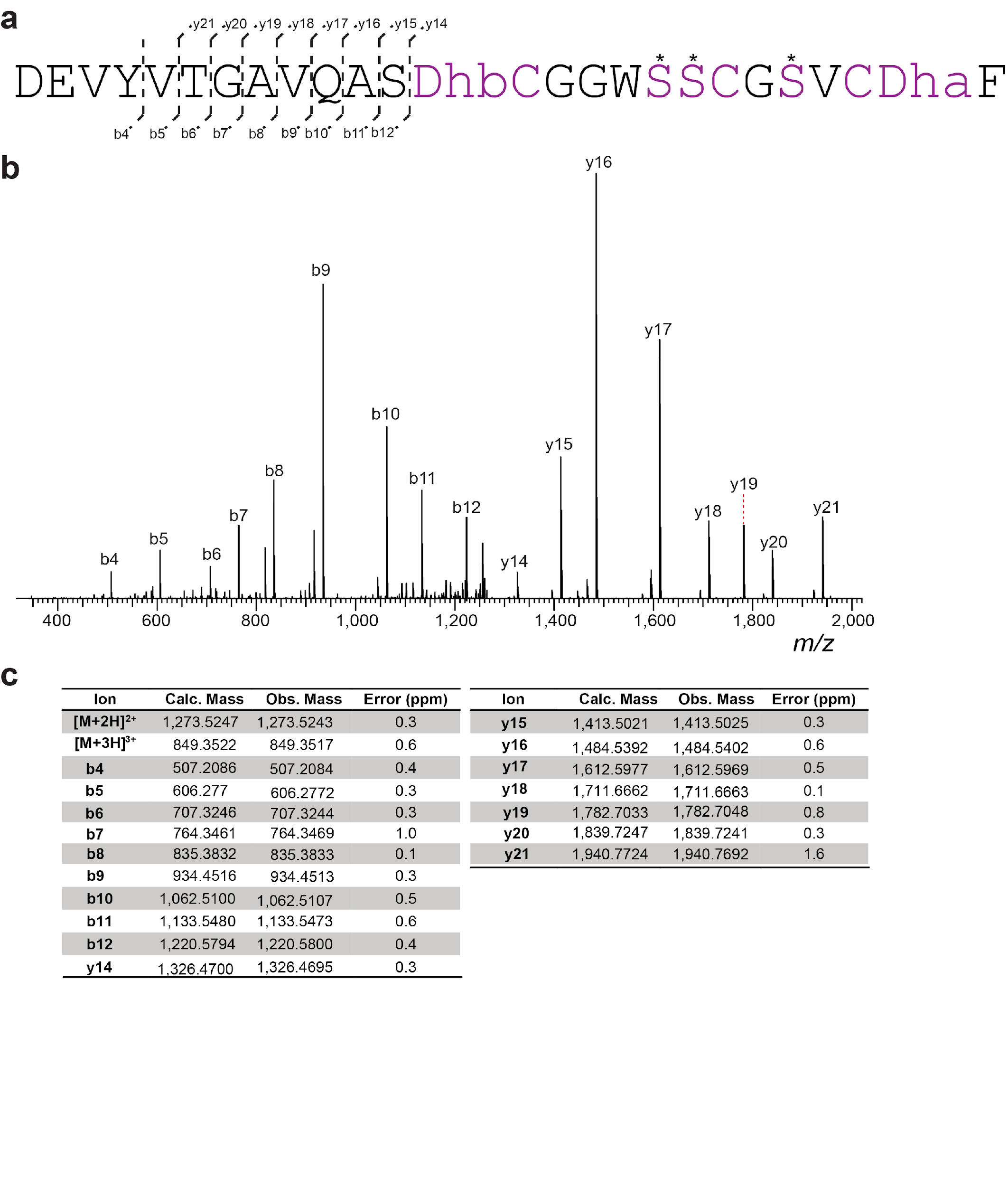


**Supplementary Fig. 19. HR-ESI tandem mass spectrometry analysis of LanII-56 after digestion of full-length product with endoproteinase GluC**. **a)** Core peptide sequence and observed MS/MS ions. Possible dehydrated Ser and Thr residues are marked with asterisks (three of five residues are dehydrated). **b)** Tandem mass spectrum. **c)** Observed and calculated masses for fragments.

**
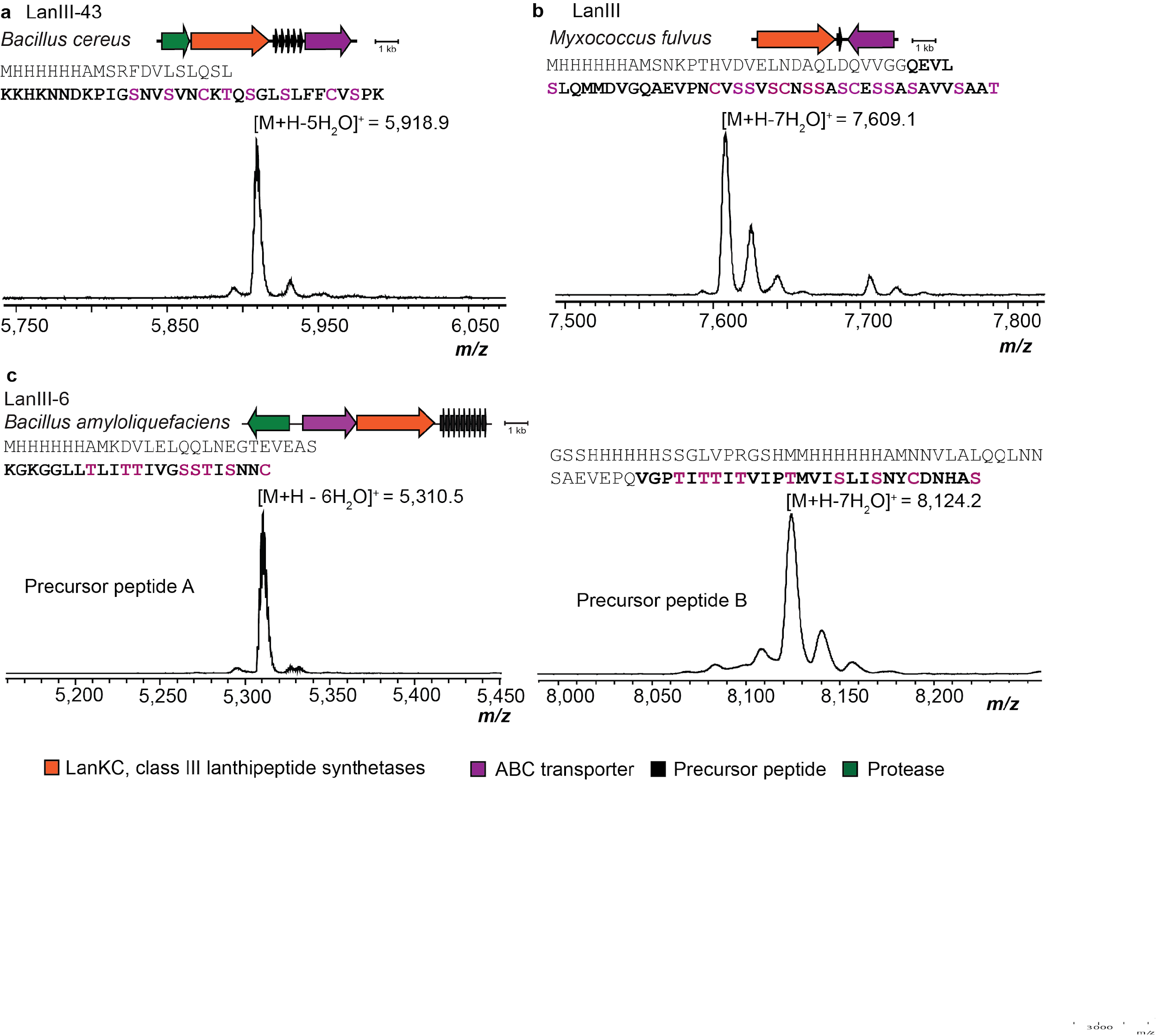
**

**Supplementary Fig. 20. Production of class III lanthipeptides in *E. coli*.** MALDI-TOF mass spectra of purified class III lanthipeptides produced in *E. coli*. Shown are the producing organism, gene diagram for the BGC, sequence of the precursor peptide with the predicted core peptide bolded, and MALDI-TOF mass spectrum of the isolated peptide post Ni-NTA purification. Calculated masses: panel a, 5-fold dehydrated peptide [M+H]^+^ monoiso., *m/z* 5,919.9 calc., 5,918.9 obs., panel b, 7-fold dehydrated peptide [M+H]^+^ monoiso., *m/z* 7,613.5 calc., 7,609.1 obs., panel c left, 6-fold dehydrated peptide [M+H]^+^ monoiso., *m/z* 5311.1 calc., 5310.5 obs., panel C right, 7-fold dehydrated peptide [M+H]^+^ monoiso., *m/z* 8119.2 calc., 8,124.2 obs.


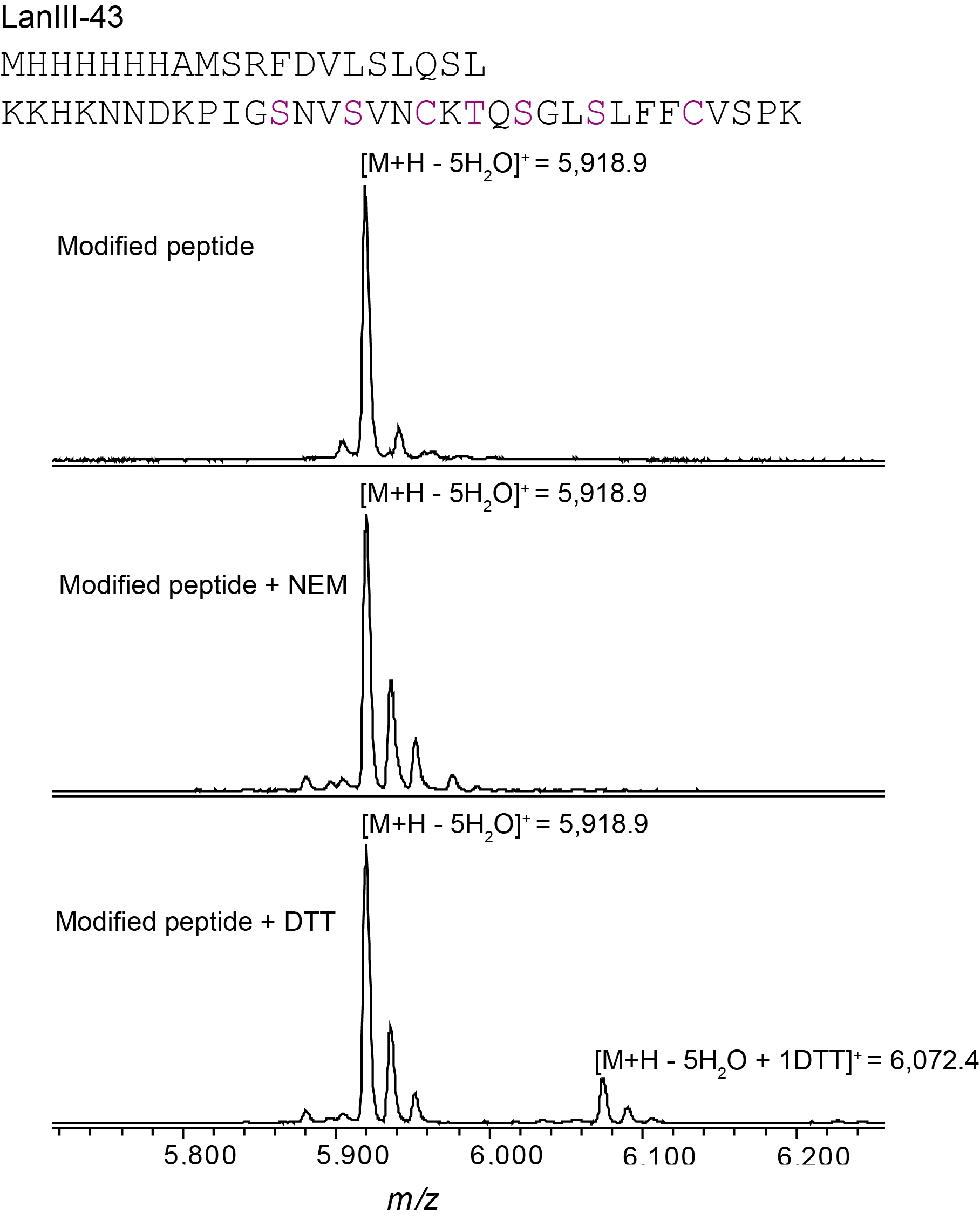


**Supplementary Fig. 21. Characterization of LanIII-43 produced in *E. coli*.** MALDI-TOF mass spectra of NEM and DTT assays with LanIII-43.

**
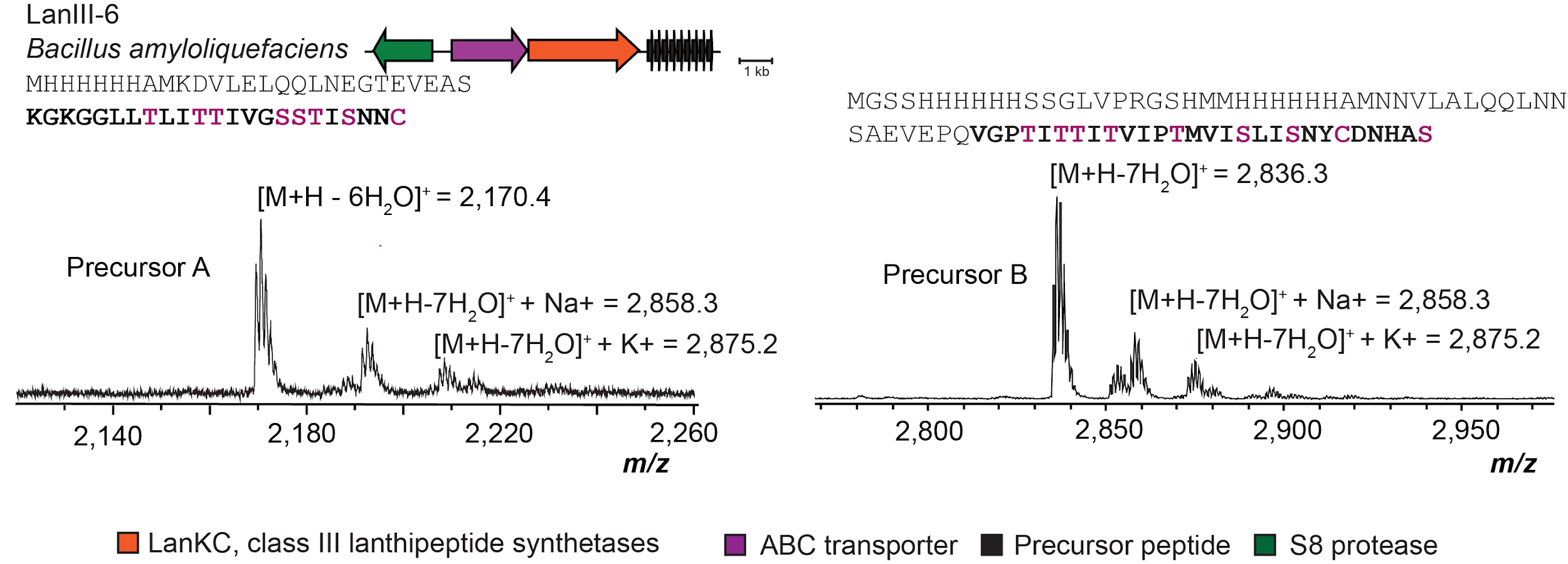
**

**Supplementary Fig. 22. Removal of leader sequence of LanIII-6 peptides by the S8 protease encoded in the BGC.** MALDI-TOF mass spectra of purified class III-6 lanthipeptides produced in *E. coli*. Shown are the producing organism, gene diagram for the BGC, sequence of the precursor peptide with the predicted core peptide bolded, and MALDI-TOF mass spectrum of S8 protease digested peptide after Ni-NTA purification. Calculated masses: left panel, 6-fold dehydrated peptide [M+H]^+^ monoiso., *m/z* 2170.6 calc., 2170.4 obs., panel C right, 7-fold dehydrated peptide [M+H]^+^ monoiso., *m/z* 2836.4 calc., 2836.3 obs.


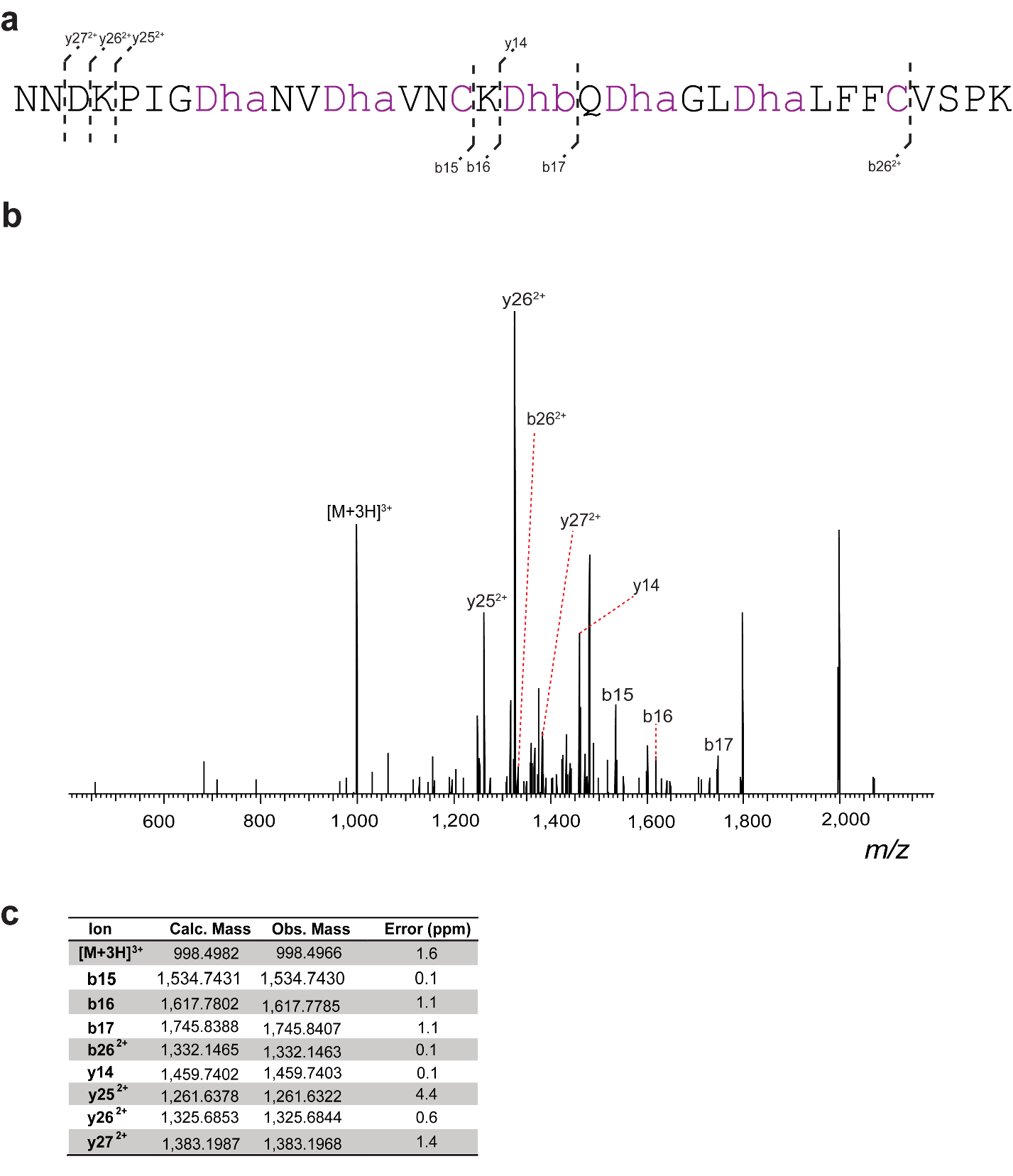


**Supplementary Fig. 23. HR-ESI tandem mass spectrometry analysis of LanIII-43 digested with trypsin**. **a)** Core peptide sequence, macrocycle location, and observed MS/MS ions. **b)** Tandem mass spectrum. **c)** Observed and calculated masses for fragments.

**
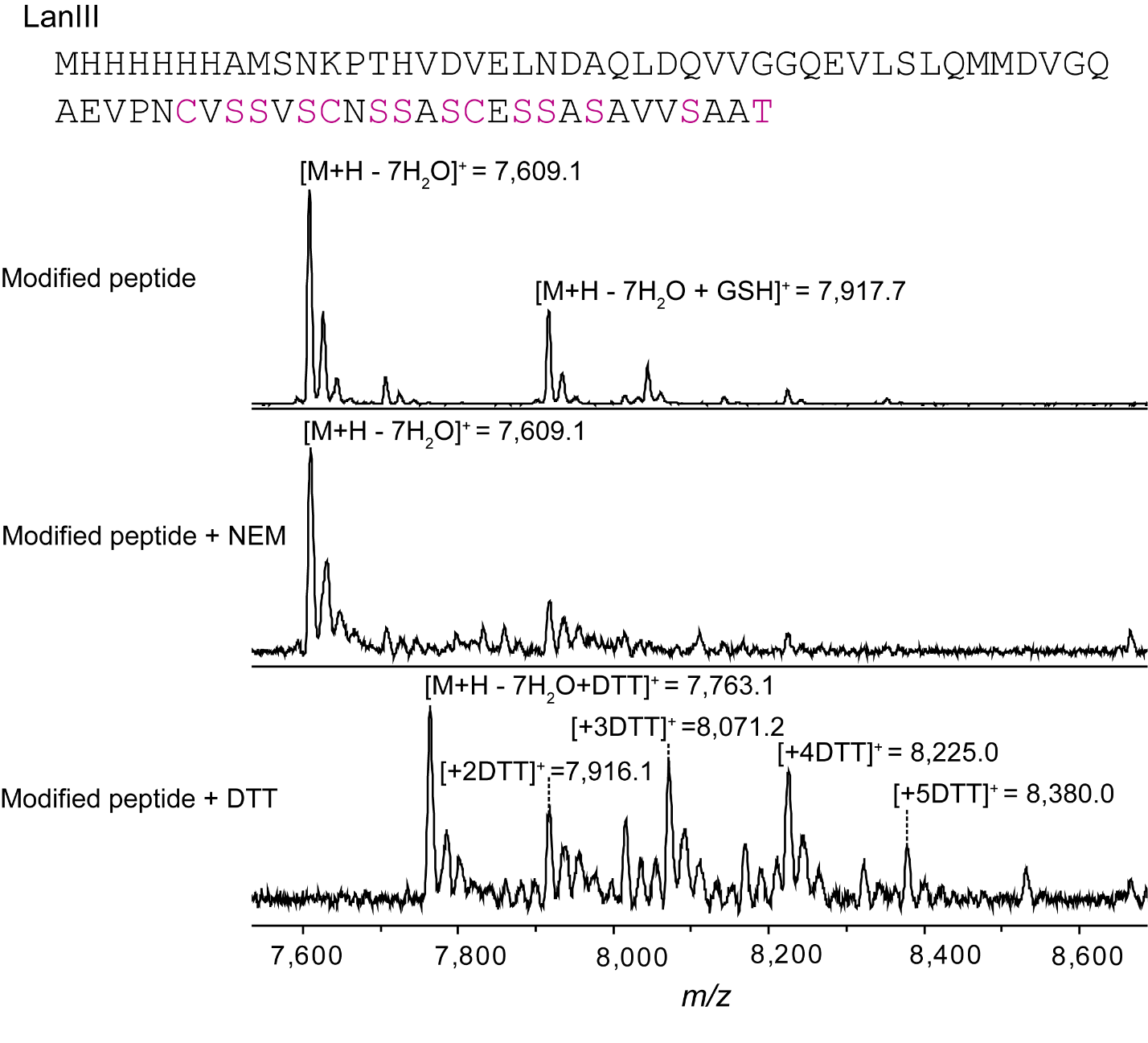
**

**Supplementary Fig. 24. Characterization of modified LanIII peptide derived from *Myxococcus fulvus* and produced in *E. coli*.** MALDI-TOF mass spectra of NEM and DTT assays with LanIII peptide.


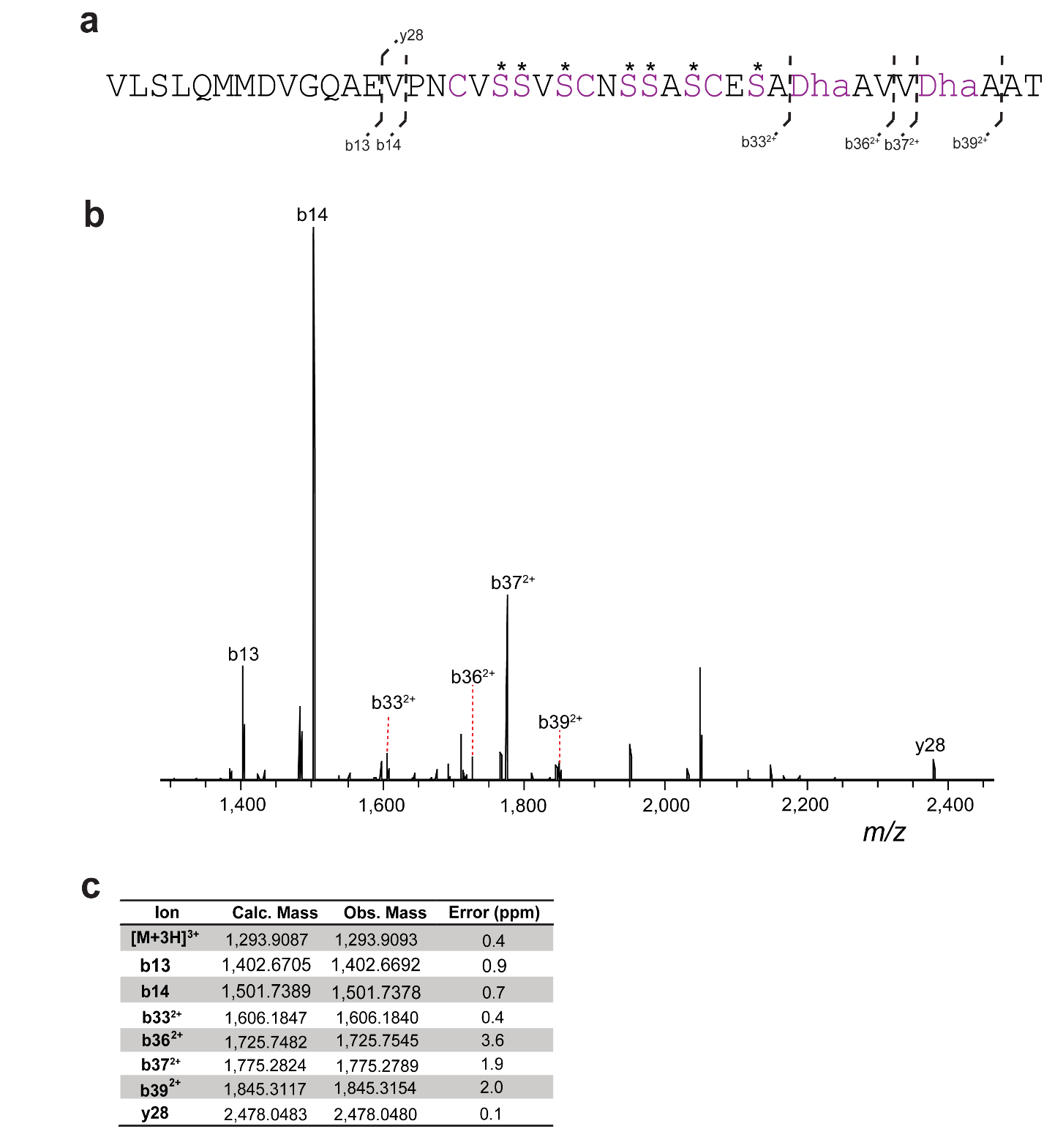


**Supplementary Fig. 25. HR-ESI tandem mass spectrometry analysis of LanIII peptide digested with endoproteinase GluC.** Possible dehydrated Ser residues are marked with asterisks (five of seven starred residues are dehydrated). **a)** Sequence of the C-terminal peptide after GluC treatment and observed MS/MS ions. **b)** Tandem mass spectrum. **c)** Observed and calculated masses for fragments.

**
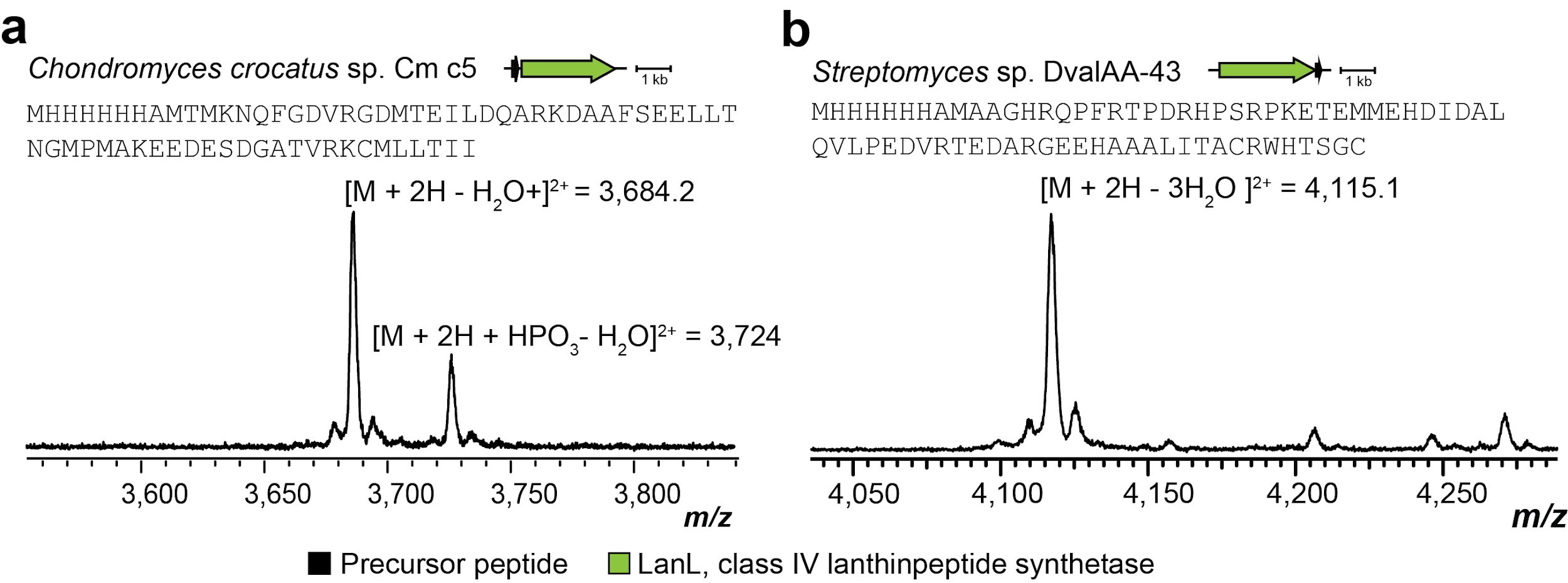
**

**Supplementary Fig. 26. Production of class IV lanthipeptides in *E. coli*.** MALDI-TOF mass spectra of purified class IV lanthipeptides produced in *E. coli*. Shown are the producer organism, gene diagram for the BGC, sequence of the precursor peptide, and MALDI-TOF mass spectrum of the isolated peptide post Ni-NTA purification. Calculated masses: panel a, singly dehydrated [M+H]^2+^ monoiso., *m/z* 3,684.7 calc., 3,684.2 obs., panel b, 3-fold dehydrated [M+H]^2+^ monoiso., *m/z* 4,115.4 calc., 4,115.1 obs.


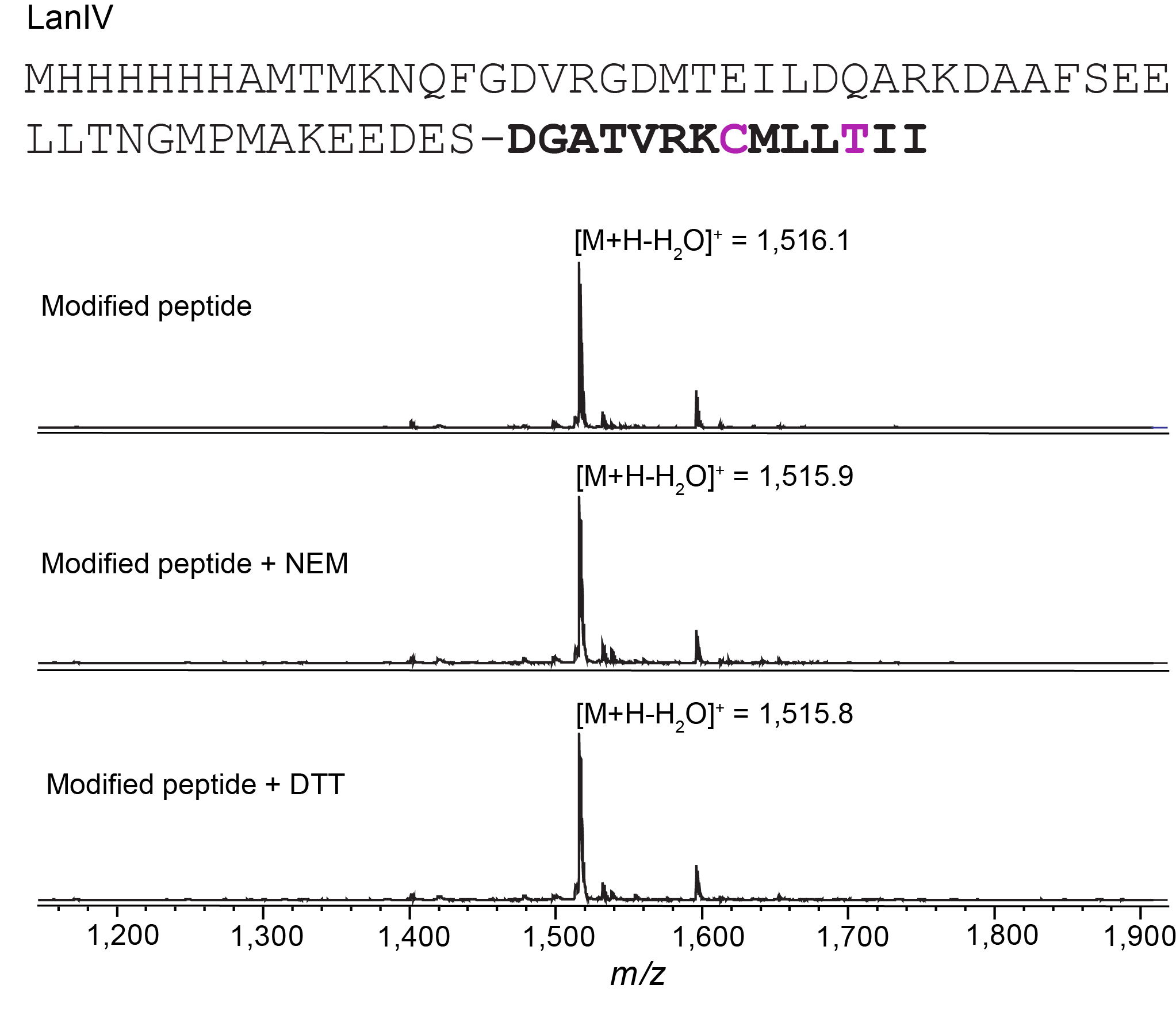


**Supplementary Fig. 27. Characterization of modified LanIV peptide derived from *Chondromyces crocatus* and produced in *E. coli*.** MALDI-TOF mass spectra of NEM and DTT assays with isolated peptide digested with endoproteinase AspN.


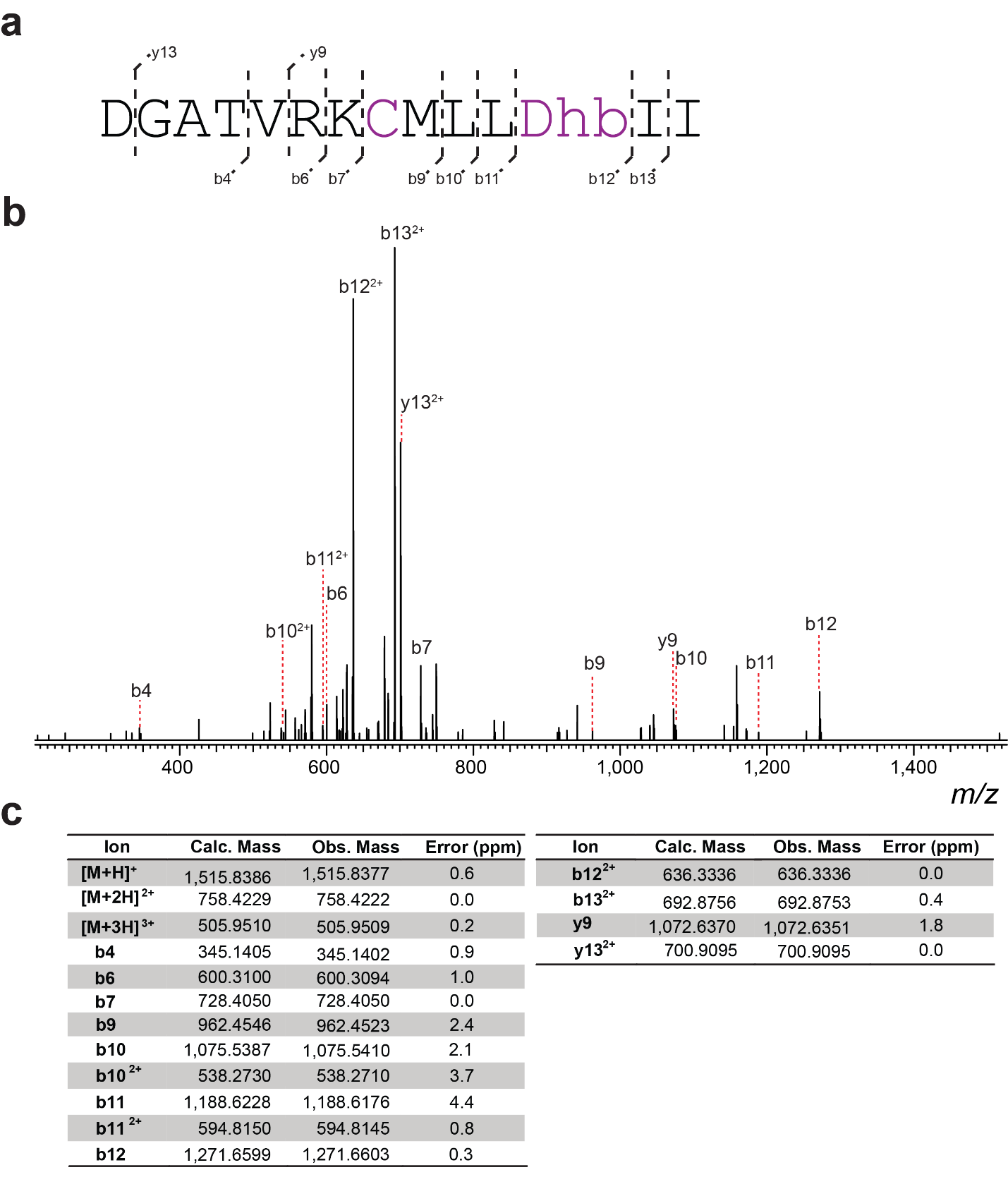


**Supplementary Fig. 28. HR-ESI tandem mass spectrometry analysis of LanIV from *Chondromyces crocatus* digested with endoproteinase AspN.** **a)** Sequence of the C-terminal peptide after AspN treatment and observed MS/MS ions. **b)** Tandem mass spectrum. **c)** Observed and calculated masses for fragments.

**
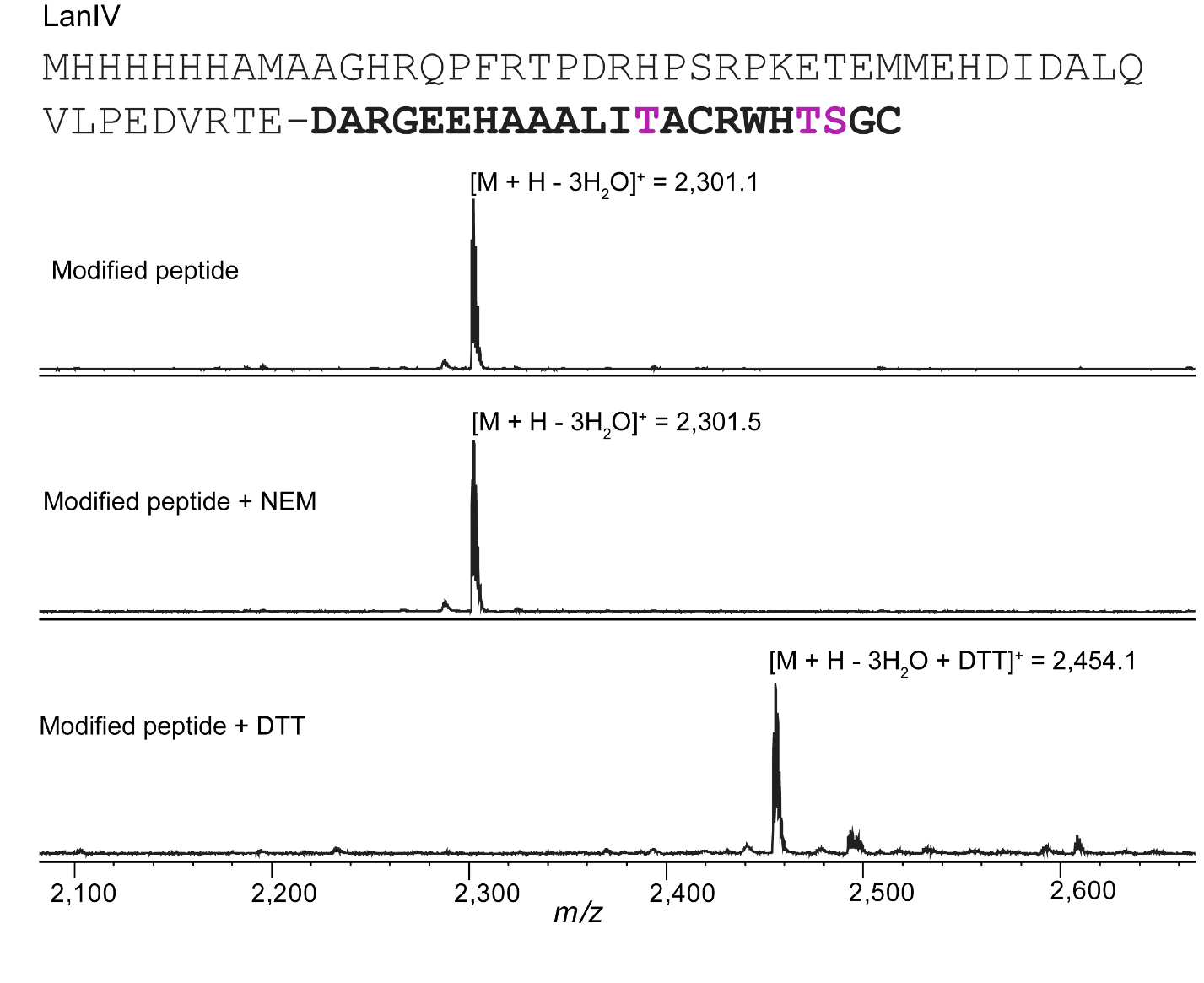
**

**Supplementary Fig. 29. Characterization of modified LanIV peptide derived from *Streptomyces sp.* SID4936 and produced in *E. coli*.** MALDI-TOF mass spectra of NEM and DTT assays with isolated LanIV peptide digested with endoproteinase AspN.

**
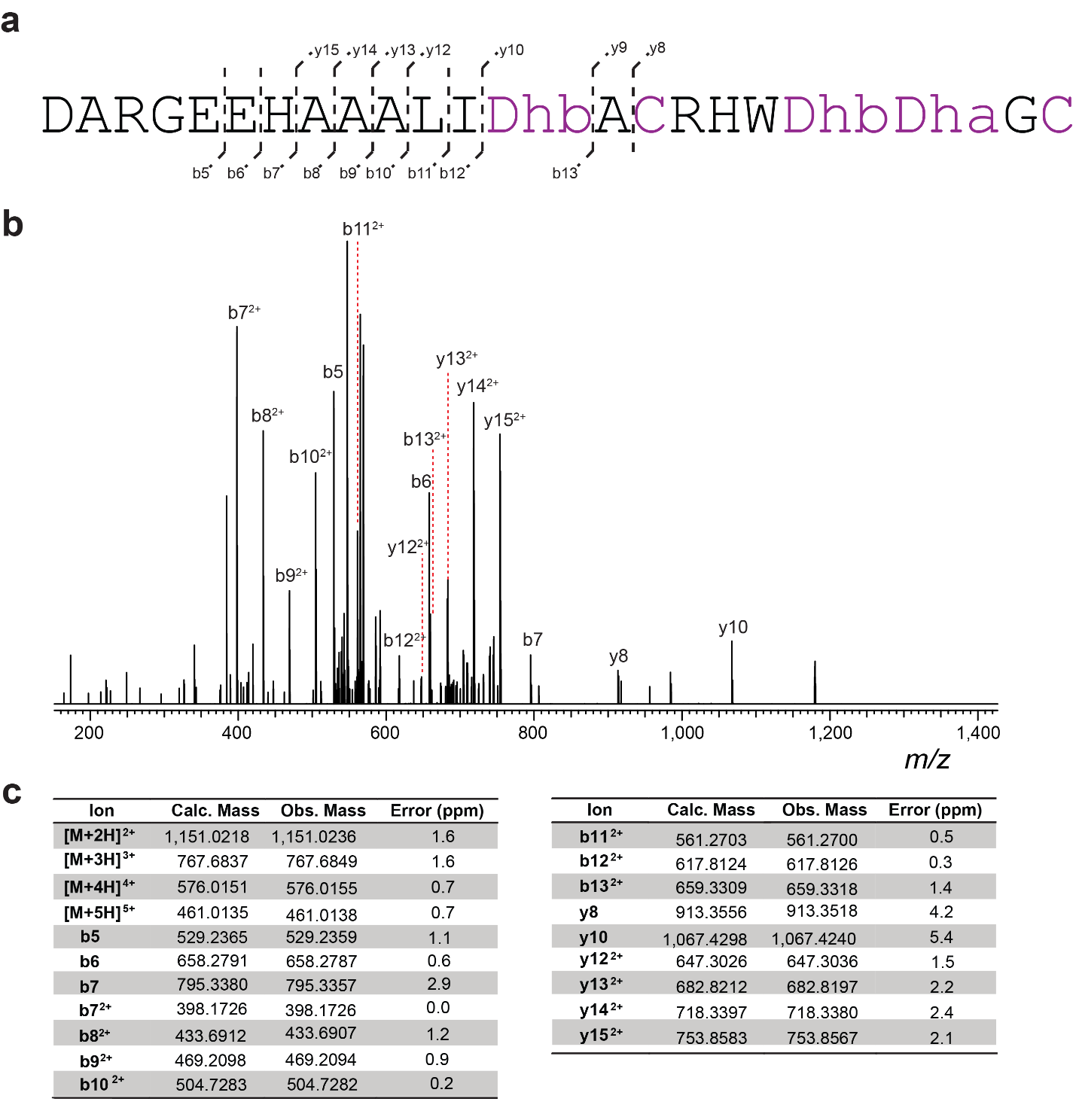
**

**Supplementary Fig. 30. HR-ESI tandem mass spectrometry analysis of LanIV peptide derived from *Streptomyces* sp. SID4936 after digestion with endoproteinase AspN**. **a)** Sequence of the C-terminal peptide after AspN treatment and observed MS/MS ions. **b)** Tandem mass spectrum. **c)** Observed and calculated masses for fragments.

**
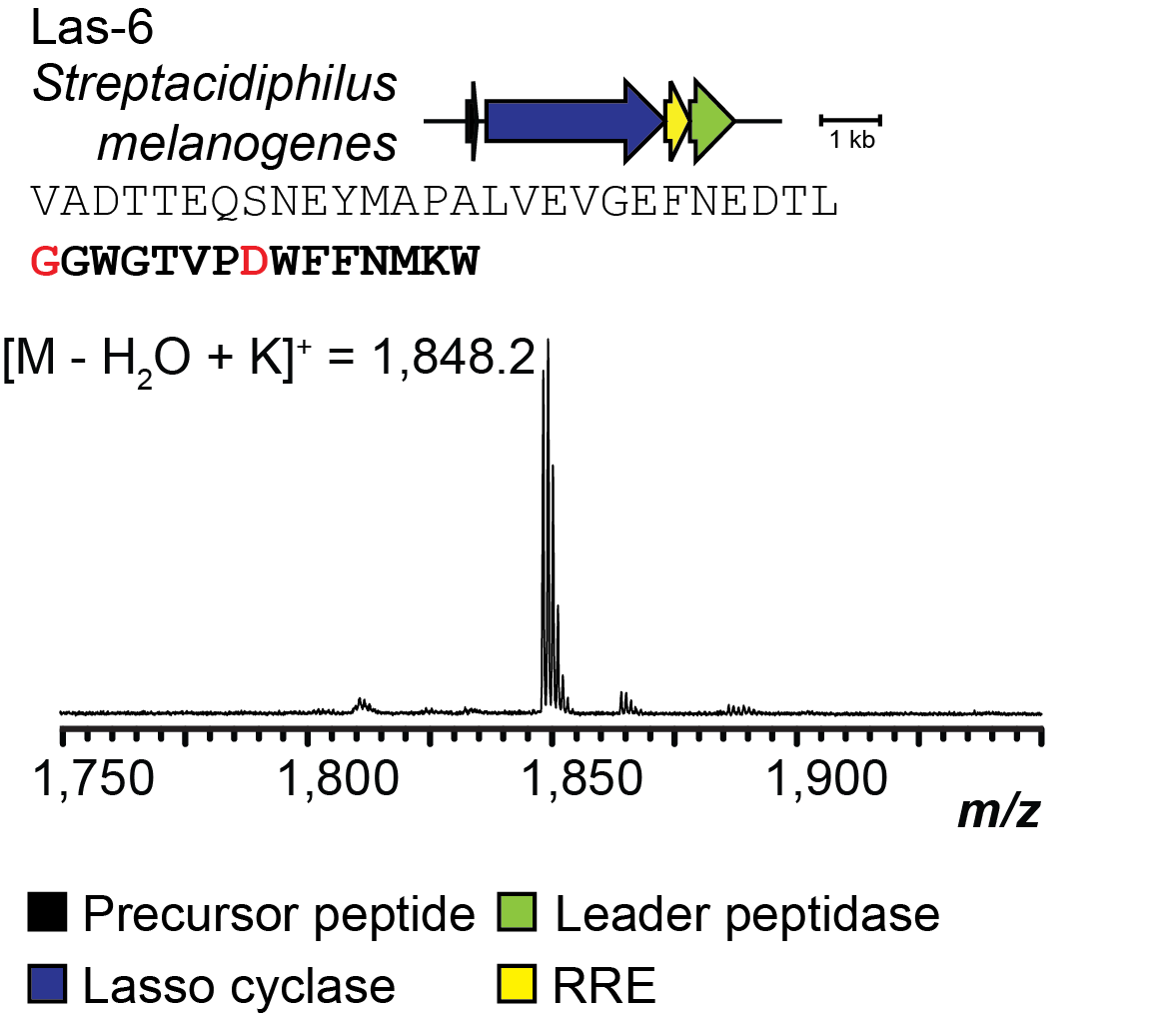
**

**Supplementary Fig. 31. Production of lasso peptides in *E. coli*.** MALDI-TOF mass spectrum of purified lasso peptide produced in *E. coli*. Shown are the producing organism, sequence of the precursor peptide with the core bolded and modified residues indicated in red, and MALDI-TOF mass spectrum of the isolated core peptide post HPLC purification. Calculated mass: singly dehydrated [M+H] monoiso., 1,809.8 (calc.), 1,809.8 (obs.). RRE, RiPP recognition element.


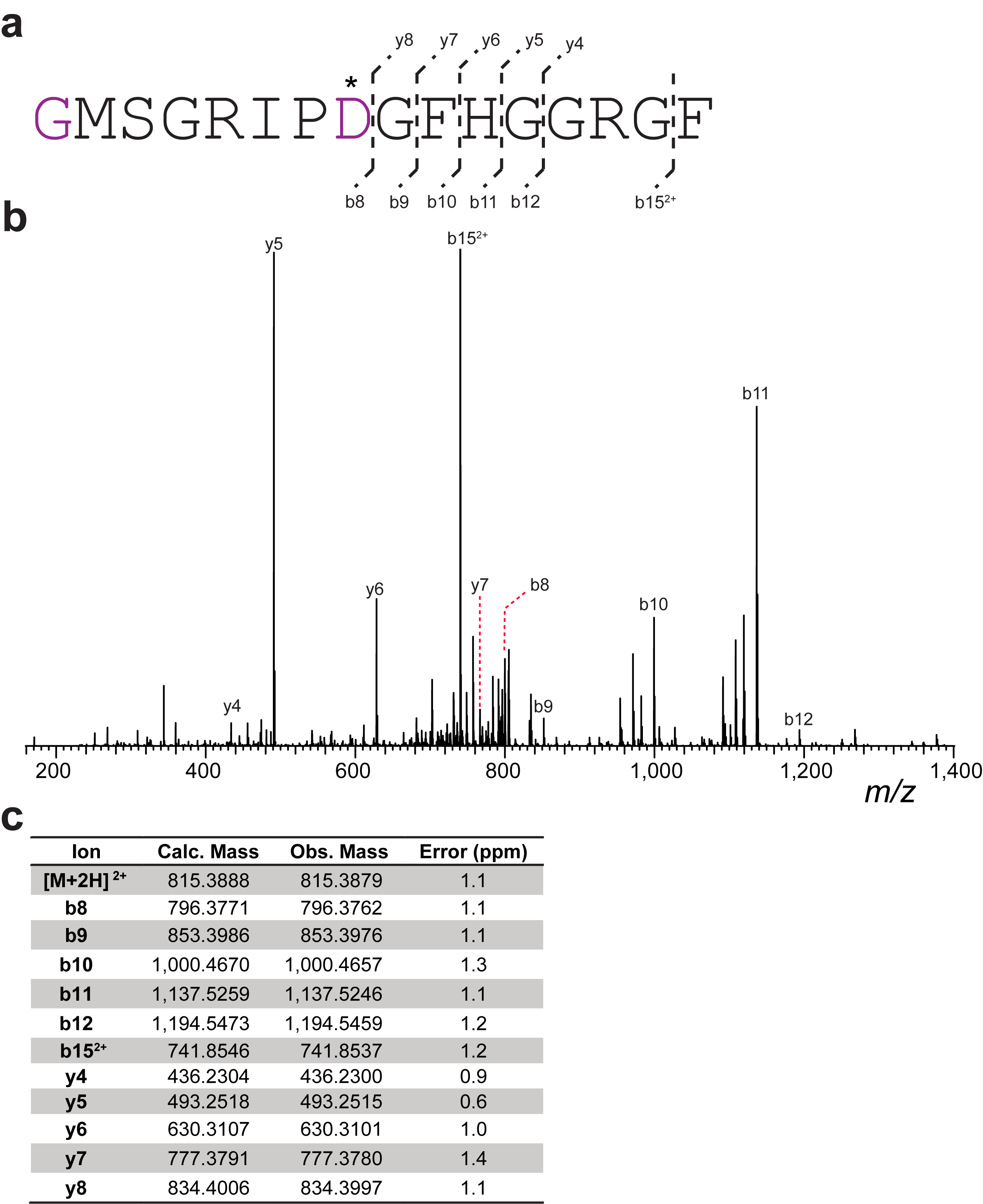


**Supplementary Fig. 32. HR-ESI MS/MS analysis of Las-2. a)** Core peptide sequence and observed MS/MS ions for Las-2. Fragments containing the residue marked with an asterisk were observed with the loss of one water molecule. **b)** Tandem mass spectrum. **c)** Observed and calculated masses for fragments.

**
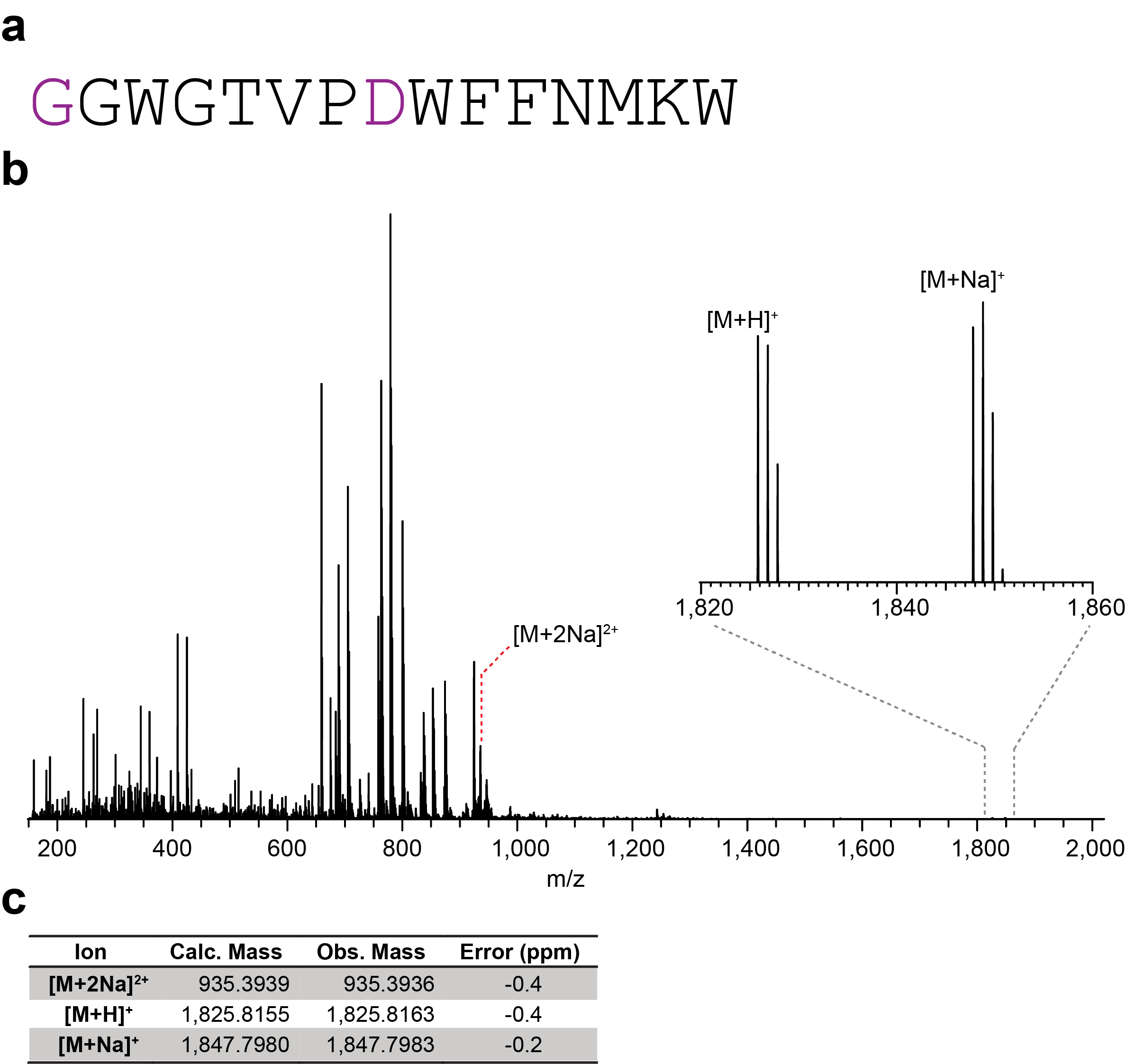
**

**Supplementary Fig. 33. HR-ESI mass spectrometry analysis of Las-6. a)** Core peptide sequence for Las-6. Residues highlighted in blue are the proposed sites of modification. **b)** HR-ESI mass spectrum. **c)** Observed and calculated masses for ions.


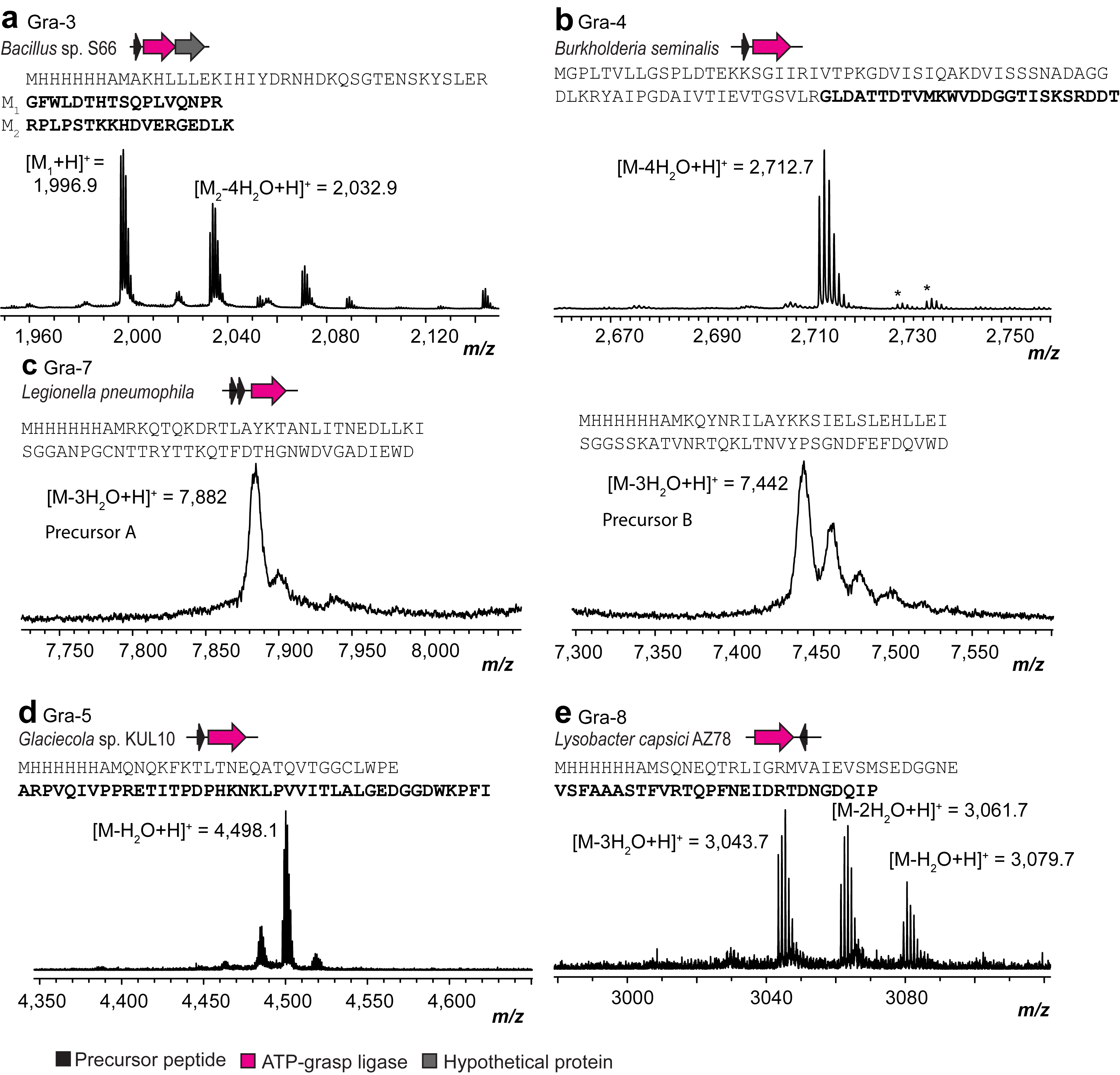


**Supplementary Fig. 34. Production of graspetides.** MALDI-TOF mass spectra of purified graspetides produced in *E. coli*. Shown are the producing organism, gene diagram for the BGC, sequence of the precursor peptide and MALDI-TOF-MS spectrum of the isolated peptide. Bolded segments are fragments represented in the MALDI-TOF mass spectra. **a)** Gra-3 after trypsin digestion. Calculated masses: unmodified [M_1_+H] monoiso. 1,996.0 calc., 1,996.9 obs., 3-fold dehydrated [M_2_+H] monoiso. 2,033.1 calc., 2,032.9 obs. **b)** Gra-4 after trypsin digestion. Calculated mass: 4-fold dehydrated [M+H] monoiso. 2,712.2 calc., 2,712.7 obs. **c)** Gra-7A after LahT150 digestion and full length Gra-7B precursor peptide. Calculated masses: Gra-7A, three-fold dehydrated [M+H] monoiso. 3,459.5 calc., 3,460.5 obs., Gra-7B, three-fold dehydrated [M+H] avg. 7,436 calc., 7,442 obs. **d)** Gra-5 after GluC digestion. Calculated mass: 1-fold dehydrated [M+H] monoiso. 4,496.5 calc., 4,498.1 obs. **e)** Gra-8 after GluC digestion. Calculated mass: 3-fold dehydrated [M+H] monoiso. 3,042.5 calc., 3,043.7 obs. Sodium and potassium adducts are indicated with asterisks.

WP_139755112.1 MKKTKQVLN-------------QITPVEATEVNGGL-------VLTSPDKERL-------

WP_143837842.1 -MEHPFTHKM------------ELTENTVEKVSAGT-------VVDIILIECKEGG----

WP_216047942.1 -MEHPFNTKM------------ELNNLSTEQVAAGS-------LAIQLPKDAKLPS----

WP_008845972.1 -MEHPFNTKM------------ELTILTTEQIAGGT-------IEHRLPKEVKLPT----

WP_199246147.1 -MCHPFNLEPDDIEAVELDFLQALGDEETAAVGGGT-------IFATTLALGEEGGCWYP

WP_144391585.1 -MKHPFALNKAELESAKDSVK-ELSVEEINAVSGAC-------GDYTTMALGEEGG----

PSB20738.1 -MRHPFDLNLDEIEAIDLEFLEELSEAESSQVD-GS-------FRSTTKALGEEGGGFPV

WP_110429223.1|Gra-5 MQNQKFK---------------TLTNEQATQVTGGCLWPEARPVQIVPPRETITPD----

: : .

WP_139755112.1 --------------TLP-KKPIRYYTQAIGEDGGDLPDLLTL------------------

WP_143837842.1 --------------PVPTKRPPMGTTMAIGEEGGGFCY----------------------

WP_216047942.1 --------------RSP-IKPPMETTMAIGEEGGSFGNWF--------------------

WP_008845972.1 --------------PTP-IKPPMETTMAIGEEGGSFGNWY--------------------

WP_199246147.1 RYPYFPSKPYPLPLPVE-KEPPVFTTQAVGEEGGGICPPSLCQF----------------

WP_144391585.1 ----------------------DYTTLAIGEEGGEGPQPTTMAIGEEGGDYTTLALGEEG

PSB20738.1 PAPKPYPSPRSHPRPHP-IAPPEVTTLALGEEGGEWATTLALGE-EGGDVVTTMAIGEEG

WP_110429223.1|Gra-5 --------------PHK-NKLPVVITLALGEDGGDWKPFI--------------------

* *:**:**

WP_139755112.1 -----

WP_143837842.1 -----

WP_216047942.1 -----

WP_008845972.1 -----

WP_199246147.1 -----

WP_144391585.1 GDWGF

PSB20738.1 RFQIQ

WP_110429223.1|Gra-5 -----

**Supplementary Fig. 35. Alignment of precursor peptides from the seven graspetide BGCs most similar to Gra-5.** Similarity was determined based on the sequence of the ATP-grasp enzyme in each cluster.

WP_057943710.1 MSQVEETRLIGRKVAVETPVLDVPGNSATFATASTVRRTAPTNEIDFADNGDQIP

WP_052756140.1 MSQNEQTRLIGRMVAIEVSMSEDGGNEVSFAAASTFVRTQPFNEIDRTDNGDQIP

WP_139175200.1 --MSEQTRLIGRMVAIEAEATDAAAQDAAFVTASTLRRTGPFGEIDSADNGDHIP

WP_096377273.1 --MSEQTRLIGRMVAIEAEAIDAAAQDAAFVTASTLRRTGPLGEIDSADNGDHIP

OPD62753.1 --MSEQTRLIGRMVAIESEQMEGAEQNAAFVAATTYRRTSPFNEIDFADNGDQIP

WP_139178447.1 --MSEQTRLIGRMVAIESEQMEGAEQNAAFVAATTYRRTSPFNEIDFADNGDQIP

WP_057948389.1 --MSEQTRLIGRMVAIESEQMEGAEQNAAFVAATTYRRTSPFNEIDFADNGDQIP

WP_123647320.1 --MSEQTRLIGRMVAIESEQMEGAEQNAAFVAATTYRRTSPFNEIDFADNGDQIP

WP_064748375.1 --MSEQTRLIGRMVAIESEQMEGAAQDAAFVAATTYRRTSPFGEIDSADNGDQIP

WP_051546700.1|Gra-8 MSQNEQTRLIGRMVAIEVSMSEDGGNEVSFAAASTFVRTQPFNEIDRTDNGDQIP

*:****** **:* : :..:*.:*:* ** * .*** :****:**

**Supplementary Fig. 36. Alignment of the precursor peptides from the seven graspetide BGCs most similar to Gra-8. Similarity was determined based on the sequence of the ATP-grasp enzyme in each cluster.**

WP_102812961.1 MSKHLLLENIHIYSKNYNRKLGTDGSIYSLERGYWINNNTSLPLVNDPNRPLPST

WP_221840852.1 MSKHLLLENIHIYSKNYNRKLGTDGSIYSLERGYWINNITSLPLVKDPNRPLPST

WP_071458443.1 MAKHILLDKIHIYEKNYDKRSGTEDSMYSTEKGYWLDNSTIKPLVLNPKRPLPST

WP_091482313.1 MVKHLLLDNIHIYEKKSDKFEGTEGSFYSSKRGFWLEKVSTNPLVQDPRRPLPSS

WP_061687330.1 MAKHLLLEKIHIYEKNHDKQSGTEGSKYSLERGFWLDIHSSQPLVQNQRRPLPST

WP_153592772.1 MARHLLLEKIHIYDRNHDKQSGTENSKYSLERGFWLDIHTSQPLVQNPRRPLPST

WP_078178751.1 MARHLLLEKIHIYDRNHDKQSGTENSKYSLERGFWLDIHTSQPLVQNPRRPLPST

WP_061791498.1 MGKHLLLDNIYIYEEKHDKKMGTLGSQYSTERGFWLIEKNLQPLVQDPRRPLPST

WP_040204049.1 MAKHLLLDKIHIYEKQYDKRFGTEGSQYSPERGFWLIGDTMQPLVEDSRRPLPST

WP_121868132.1|Gra-3 MAKHLLLEKIHIYDRNHDKQSGTENSKYSLERGFWLDTHTSQPLVQNPRRPLPST

* .*:**::*:**. : :. ** .* ** :.*:*: . *** : .*****:

WP_102812961.1 KKHDVERGEDLK

WP_221840852.1 KKHDVERGEDLK

WP_071458443.1 KKHDVERGEDQK

WP_091482313.1 KKHDVERGEDKK

WP_061687330.1 KKCDVERGEDLK

WP_153592772.1 KKHDVERGEDLK

WP_078178751.1 KKHDVERGEDLK

WP_061791498.1 KKHDVERGEDLK

WP_040204049.1 KKHDVERGEDMK

WP_121868132.1|Gra-3 KKHDVERGEDLK

** ******* *

**Supplementary Fig. 37. Alignment of precursor peptides from the nine graspetide BGCs with ATP-grasp enzymes most similar to the ATP-grasp enzyme from the Gra-3 BGC.**


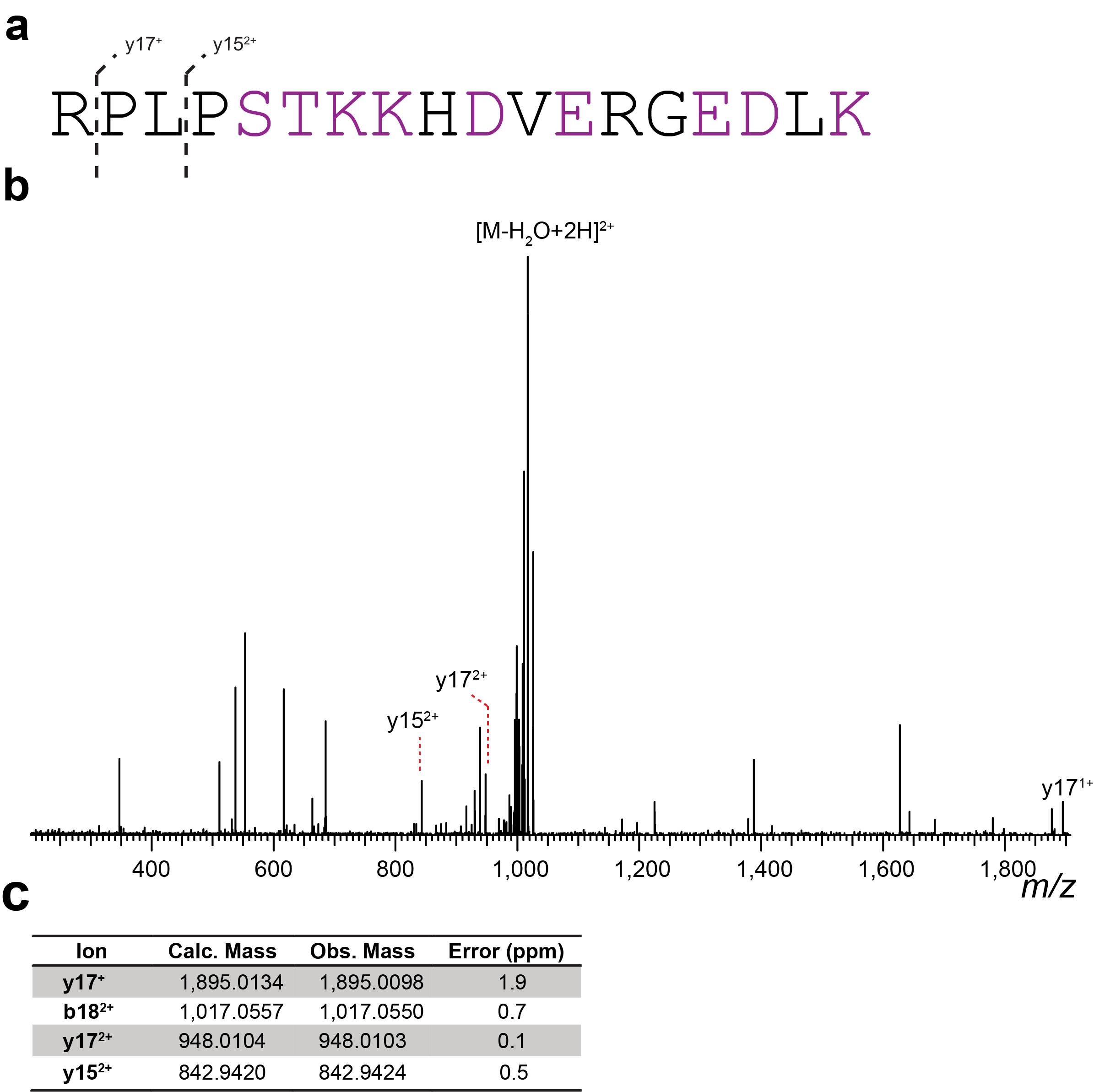


**Supplementary Fig. 38. HR-ESI tandem mass spectrometry data of Gra-3.** **a)** Sequence of the C-terminal peptide after trypsin treatment and observed MS/MS ions for Gra-3. Three of the purple donor residues are modified, and three of the four purple acceptor residues are modified. **b)** Tandem mass spectrum. **c)** Observed and calculated masses for fragments.

WP_165225913.1 MGPLTVILGSLLKGEEKSGIVRIVTPKGDVLCFNESDVVTSSSVDASGELRRYAIPADANVTIE

VTGAALRGLDVTVDSVLKWVDDGGTISKSRDDLS

WP_219940147.1 MADSKPNLVIHGYVEKDKAFPDYSRVTTATGEKLLIETKGIIKSFPIEASGESGLNRVLIEQNT

NIWMGISSESLQSFNDGNSDIGLKSIMKWVDDGGTISKSRDDTFSAGNLP

WP_192407277.1 MRDILYCFEHADQNKSDQTRLTFPNGKDIYVRTTDIEKIVPITSQSGGINGYYLKPDAQITIET

SVKDPALEAYADEEANTYPKYYDDDGYSPLKSYDDPLAAGYNIPHGPQPSPDLRLLRSGARKRV

EDTAFWPSW

WP_146203758.1 MHGSAFIPLVGSYLDKPADLKAVSDVKVVTPAGDVVRIPTDAIKHLQNVDASGELVRFFLNPEA

EIEVTIKGDMITASRAGTNTIYKYLDDGGTISKSRDDLAADPV

WP_124608905.1|Gra-4 MGPLTVLLGSPLDTEKKSGIIRIVTPKGDVISIQAKDVISSSNADAGGDLKRYAIPGDAIVTIE

VTGSVLRGLDATTDTVMKWVDDGGTISKSRDDT

**Supplementary Fig. 39. Alignment of precursor peptides from the four BGCs most similar to Gra-4.** T/SxxKxxDD repeats are highlighted in green.


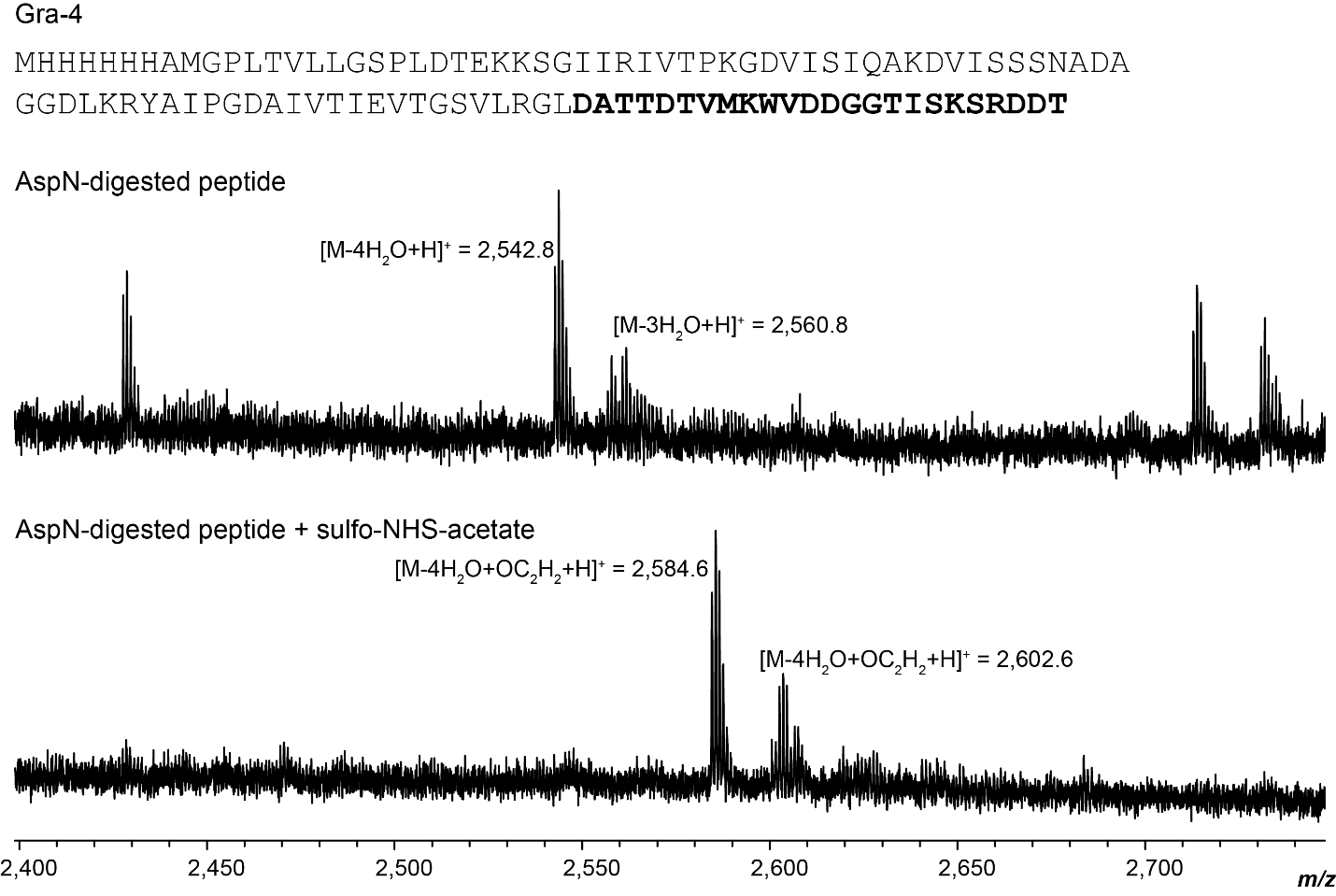


**Supplementary Fig. 40. Sulfo-NHS-acetate labeling of Gra-4.** *Top*. AspN digestion of modified peptide, with both three modifications and four modifications. Fragment in bold is the fragment after AspN digestion. Calculated mass, 4X dehydrated [M+H] monoiso. 2,542.1, obs. 2,542.8. *Bottom*. Peptide after reaction with sulfo-NHS-acetate showing one addition of acetate, corresponding to one free amine. Calculated mass: 4-fold dehydrated, monoacetylated [M+H] monoiso. 2,584.2 calc., 2,584.6 obs.


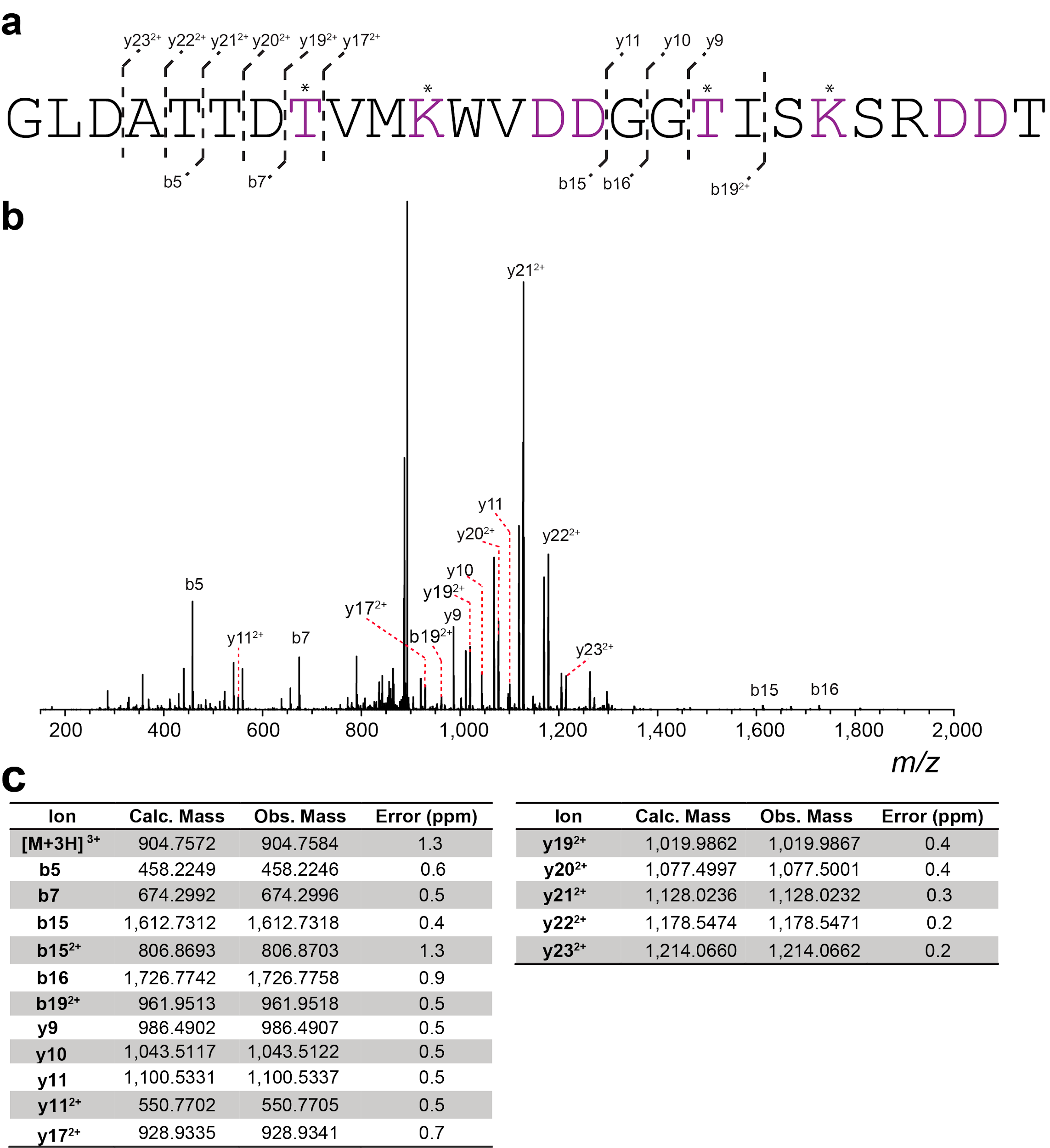


**Supplementary Fig. 41. HR-ESI tandem mass spectrometry data of Gra-4.** **a)** Sequence of the C-terminal peptide after trypsin treatment and observed MS/MS ions for Gra-4. Fragments containing a residue marked by an asterisk were observed with the loss of one water molecule per marked residue. **b)** Tandem mass spectrum. **c)** Observed and calculated masses for fragments.


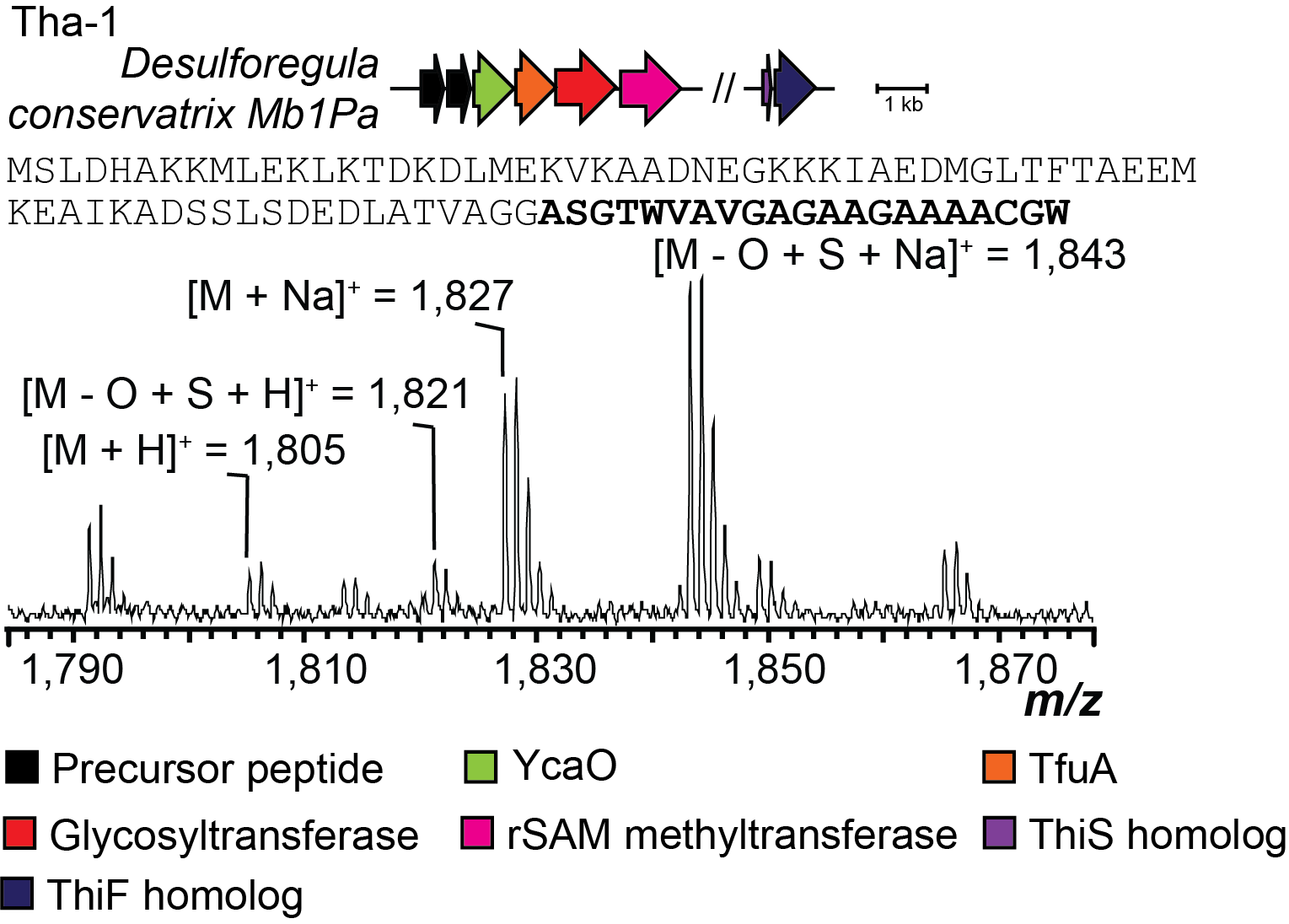


**Supplementary Fig. 42. Production of thioamitides.** MALDI-TOF mass spectra of a thioamitide produced in *E. coli*. Shown are the producing organism, gene diagram for the BGC, sequence of the precursor peptide with the putative core peptide bolded, and MALDI-TOF mass spectrum of the isolated peptide after digestion with LahT150. Calculated mass: singly thioamidated [M+H] monoiso. 1,820.8, obs. 1,821. rSAM, radical SAM enzyme.


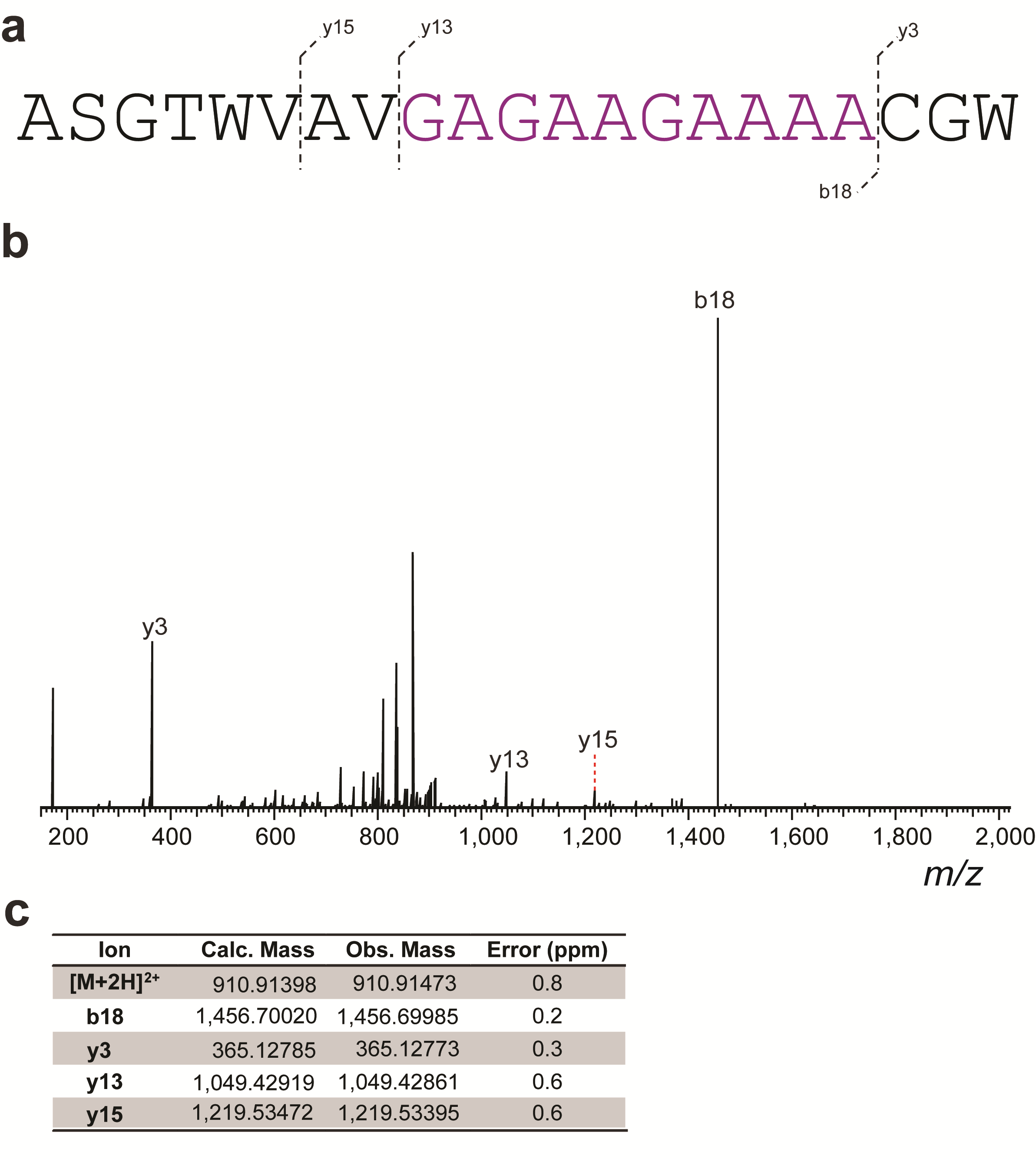


**Supplementary Fig. 43. HR-ESI tandem mass spectrum of Tha-1.** **a)** Core peptide sequence and observed MS/MS ions for Tha-1. Fragments containing the series of residues marked in purple had a mass consistent with replacement of oxygen with sulfur. Because of the incomplete fragmentation, the location of the thioamide could not be further localized. **b)** Tandem mass spectrum **c)** Observed and calculated masses for fragments.

**
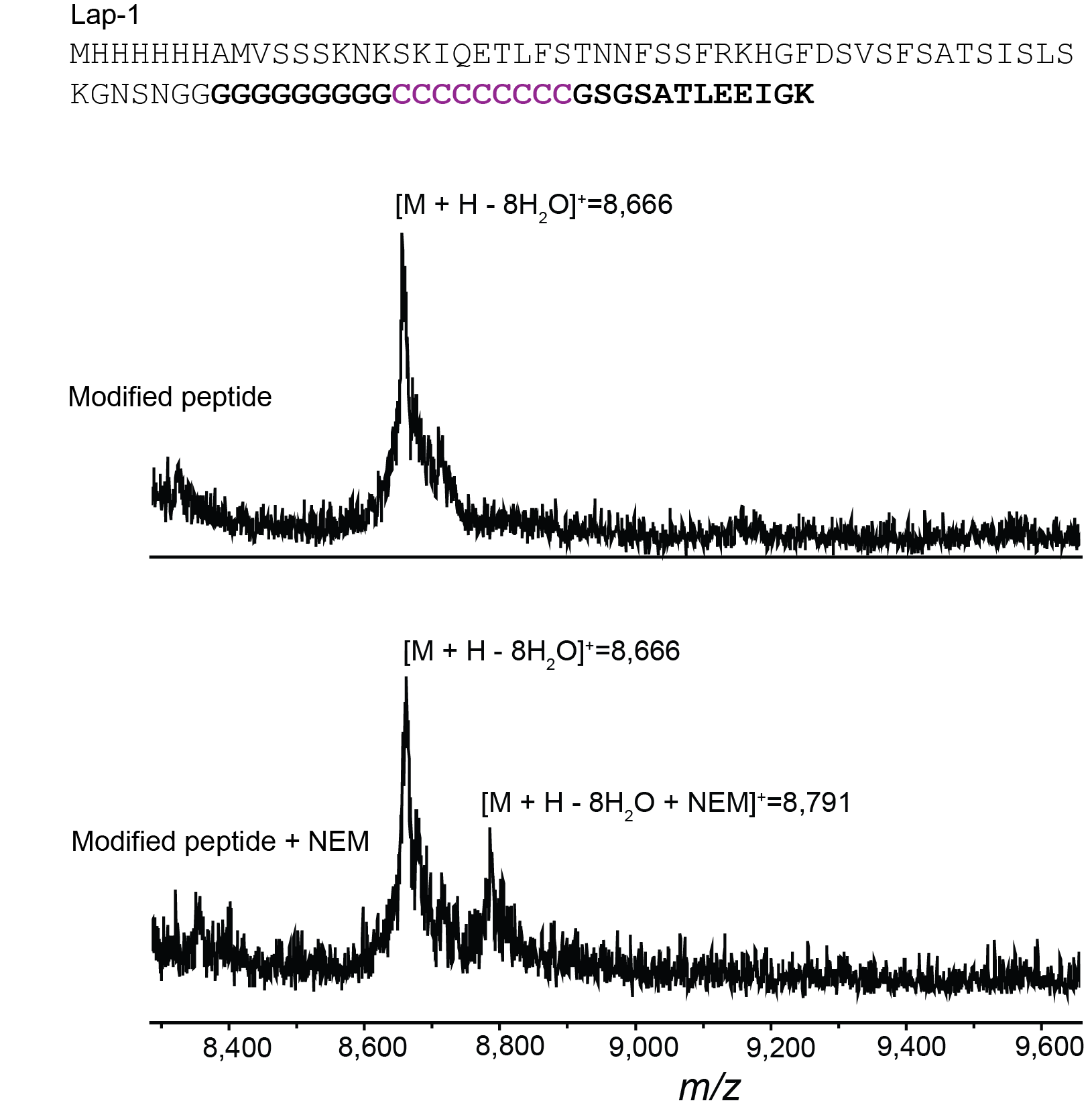
**

**Supplementary Fig. 44. NEM labeling of Lap-1**. Modified peptide with eight cyclodehydrations out of nine cysteine residues highlighted in purple. Calculated mass, 8-fold dehydrated [M+H] monoiso. 8,667, obs. 8,666. *Bottom*. Peptide after reaction with NEM showing one addition of NEM, corresponding to one free cysteine. Calculated mass, 8-fold dehydrated, NEM labeled peptide [M+H] monoiso. 8,792 calc., 8,791 obs.


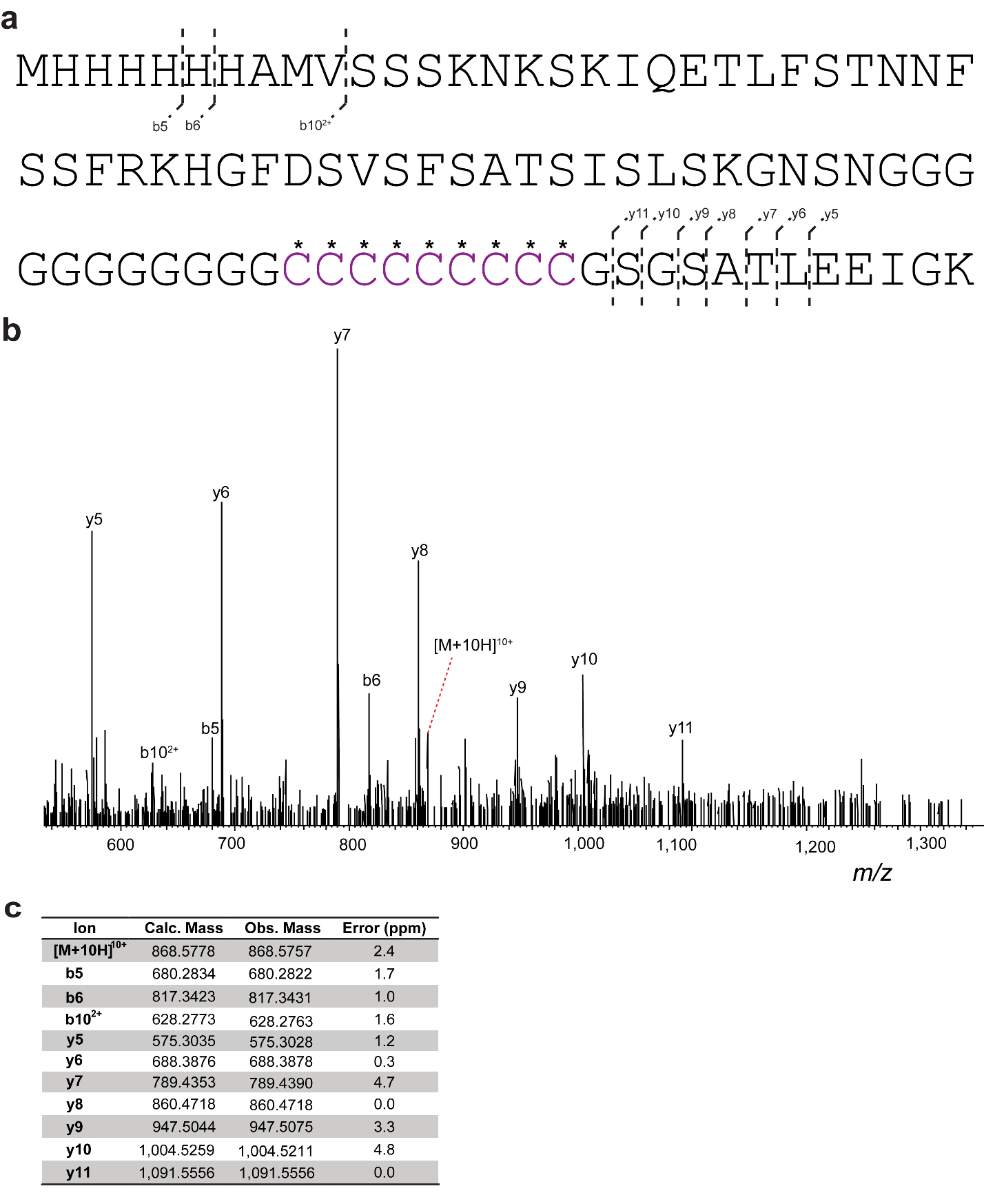


**Supplementary Fig. 45. HR-ESI tandem mass spectrometry analysis of Lap-1.** **a)** Peptide sequence and observed MS/MS ions. Possible cysteine involved in thiazol(in)e formation are marked with asterisks. **b)** Tandem mass spectrum. **c)** Observed and calculated masses for fragments.

**
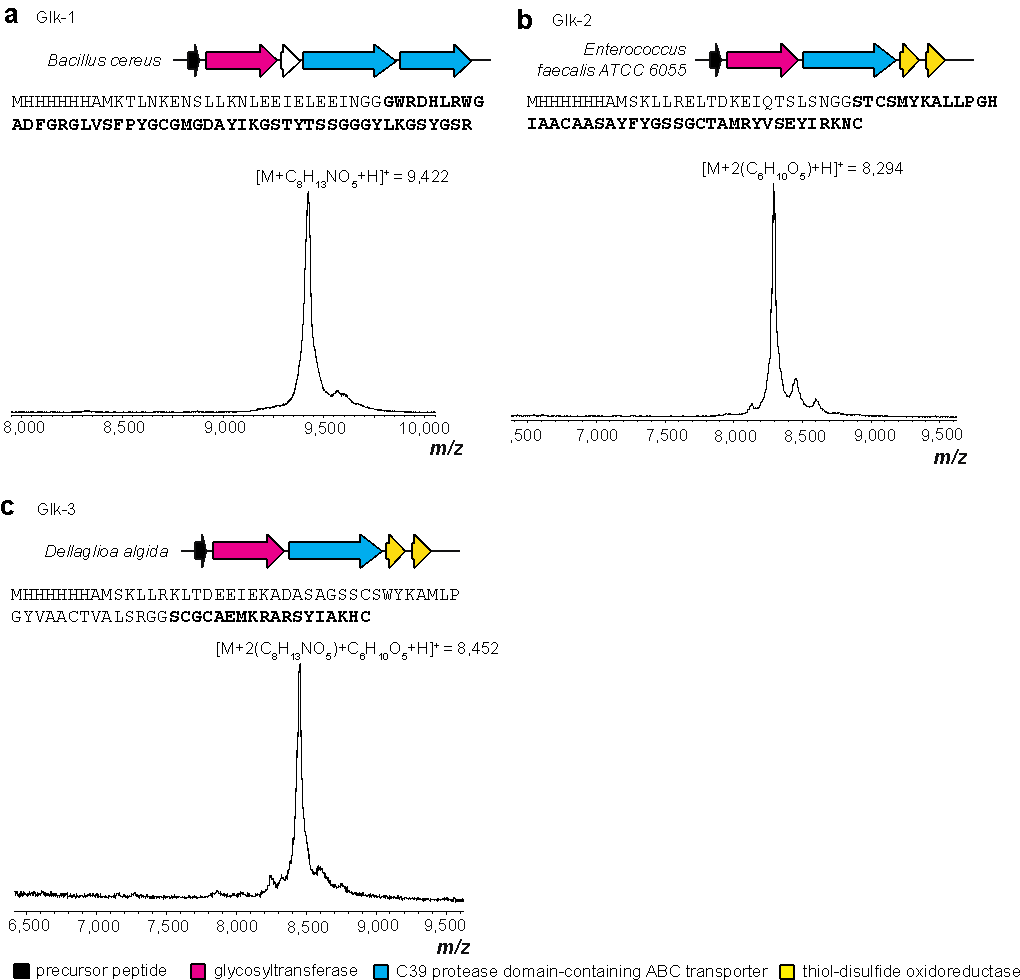
**

**Supplementary Fig. 46. MALDI-TOF mass spectra of purified glycocins with leader peptide attached**. Shown are the producing organism, gene diagram for the BGC, sequence of the precursor peptide with the predicted core peptide bolded, and MALDI-TOF mass spectra of the isolated peptide post Ni-NTA purification. Calculated masses: panel a, Monoglycosylated (*N*-Acetylglucosamine) [M+H] monoiso., *m/z* 9,420.5 calc., 9,422. obs., panel b, Diglycosylated (2 Hexoses) [M+H] monoiso., *m/z* 8,294.9 calc., 8,294 obs., panel c, Triglycosylated (Hexose + 2 *N*-Acetylglucosamines) [M+H] monoiso., *m/z* 8,452 calc., 8,452 obs.


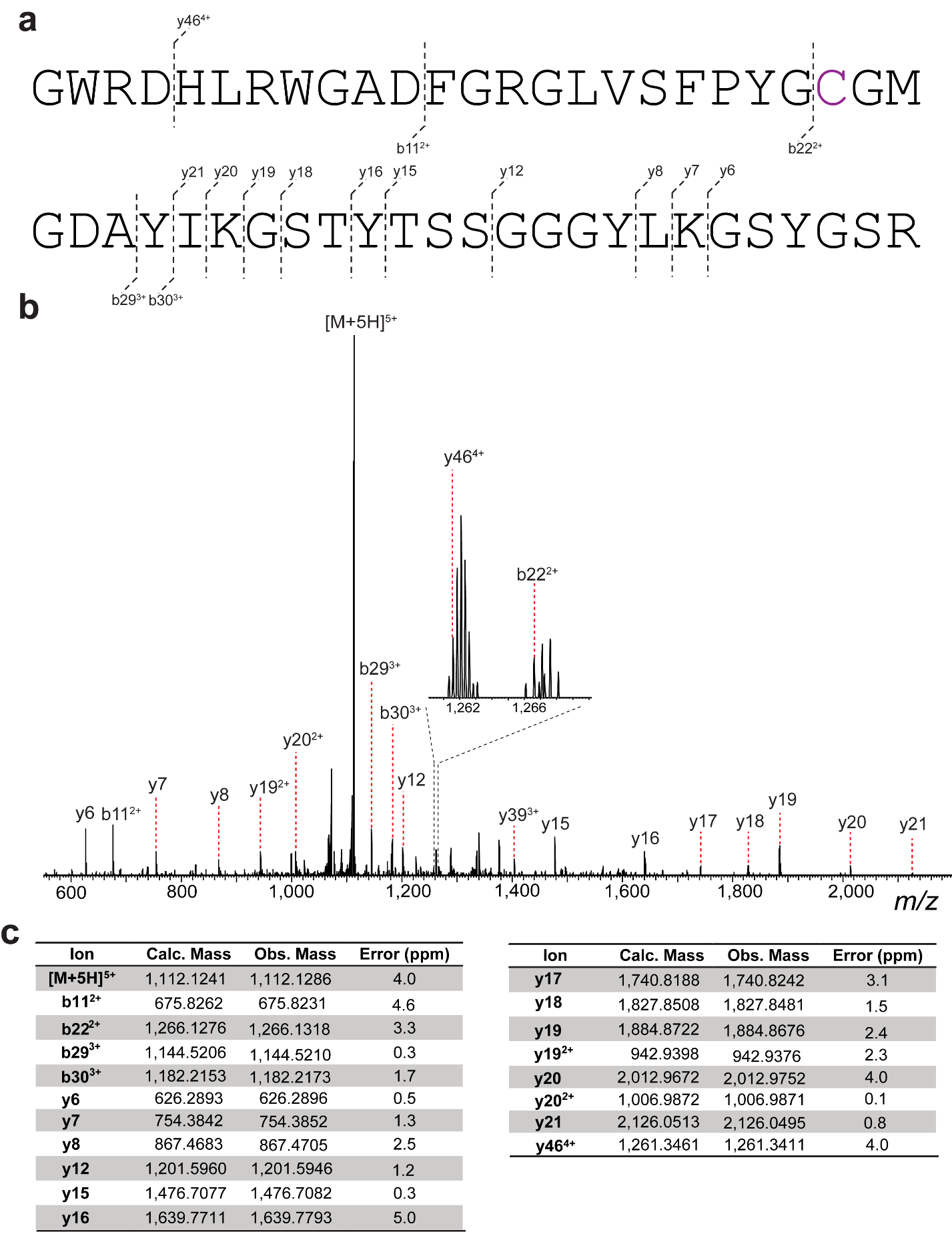


**Supplementary Fig. 47. HR-ESI tandem mass spectrometry analysis of Glk-1 produced after digestion with LahT150**. **a)** Glycosylated peptide sequence and observed MS/MS ions. The cysteine residue highlighted in purple is glycosylated with one *N*-acetylhexosamine (HexNAc). **b)** Tandem mass spectrum. **c)** Observed and calculated masses for fragments.


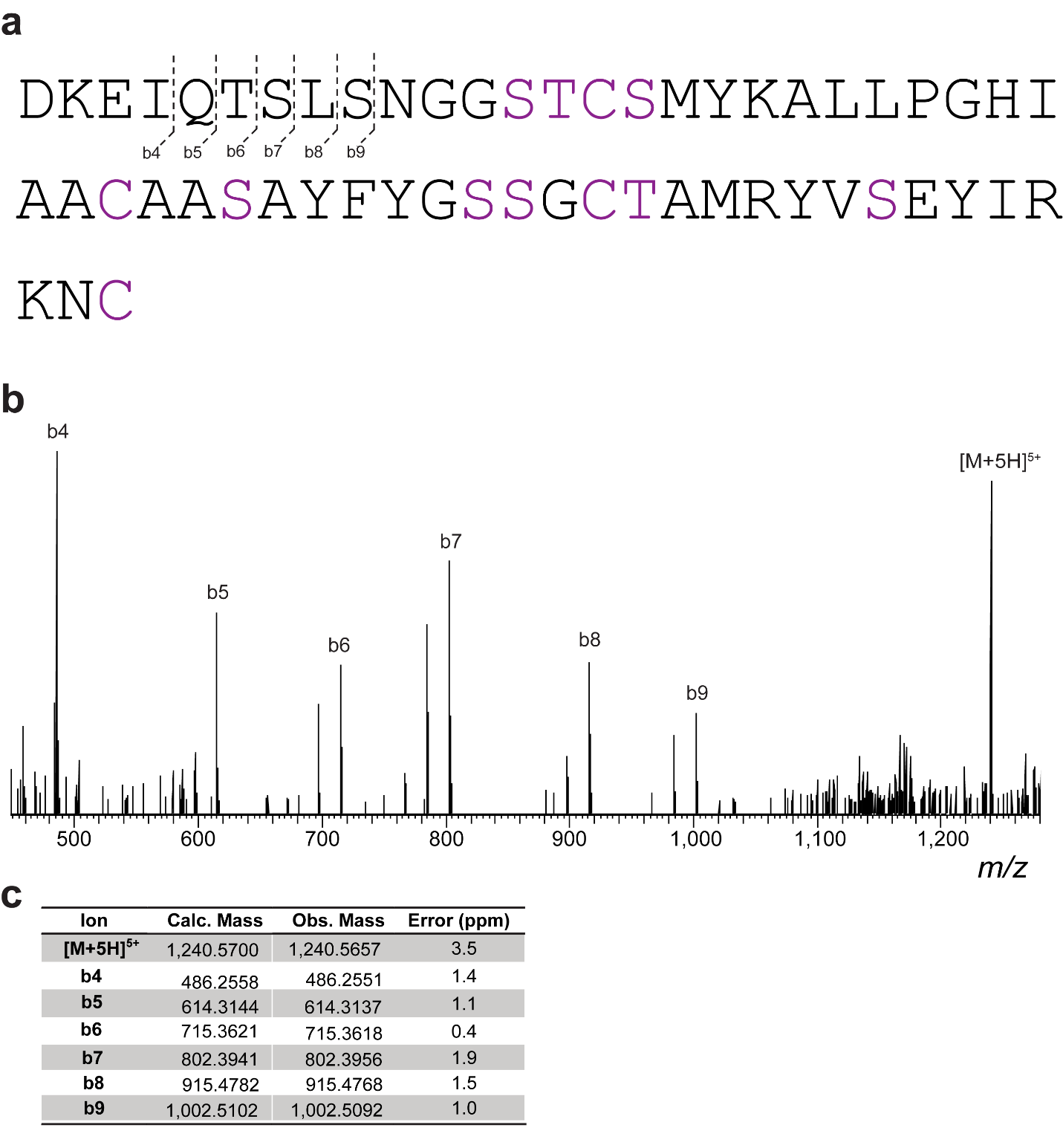


**Supplementary Fig. 48**. **HR-ESI tandem mass spectrometry analysis of Glk-2 produced after digestion with endoproteinase AspN**. **a)** Diglycosylated peptide sequence and observed MS/MS ions. Possible Ser/Thr/Cys glycosylated with one or two hexoses are highlighted in purple. **b)** Tandem mass spectrum. **c)** Observed and calculated masses for fragments.


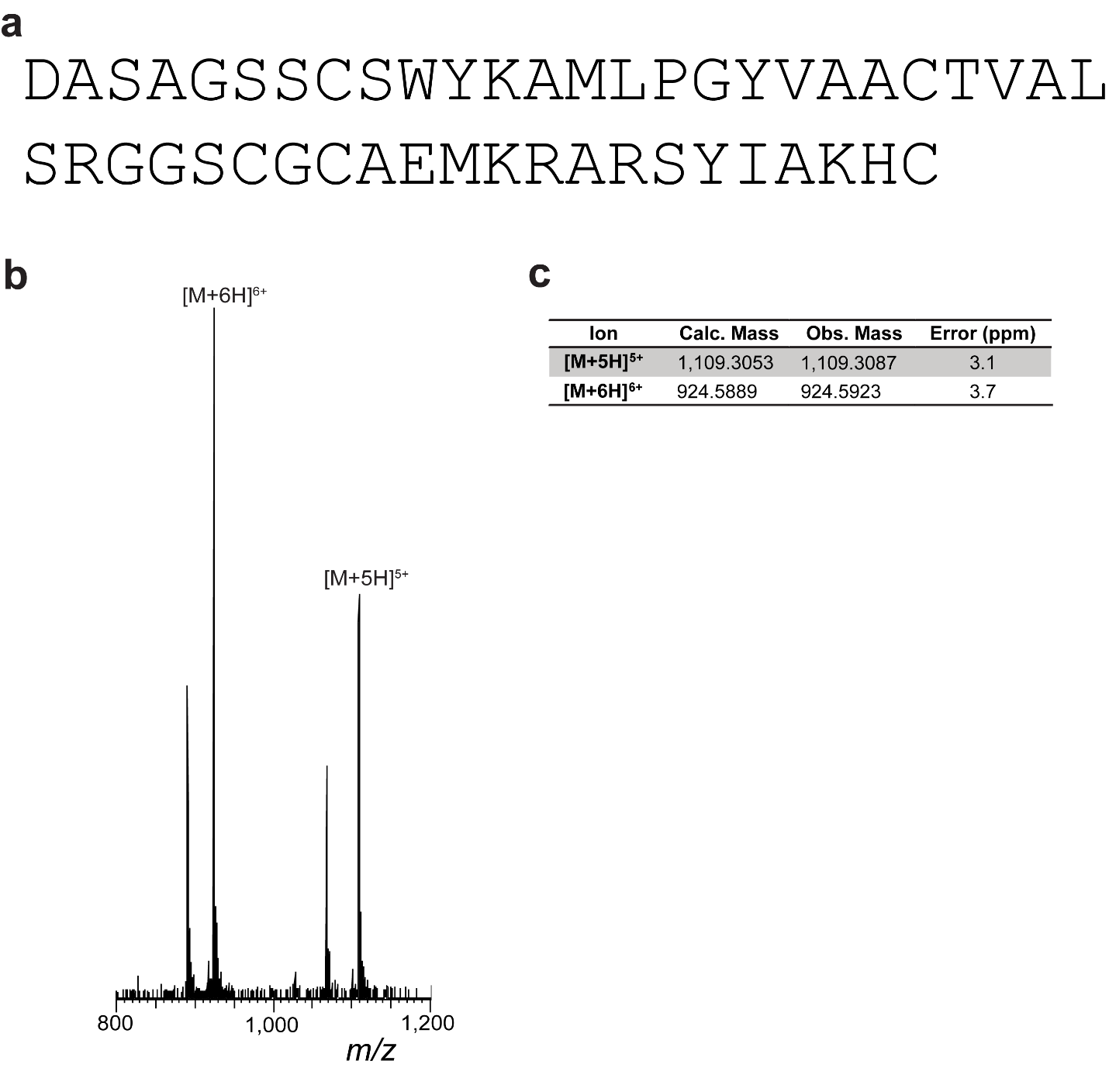


**Supplementary Fig. 49. HRMS analysis of Glk-3 produced after digestion with AspN.** **a)** Peptide sequence of AspN-cleaved C-terminal Glk-3. **b)** HR-MS spectra of AspN-cleaved C-terminal peptide modified with two N-acetylhexosamine (HexNAc) moieties and one hexose. **c)** Observed and expected masses.

**
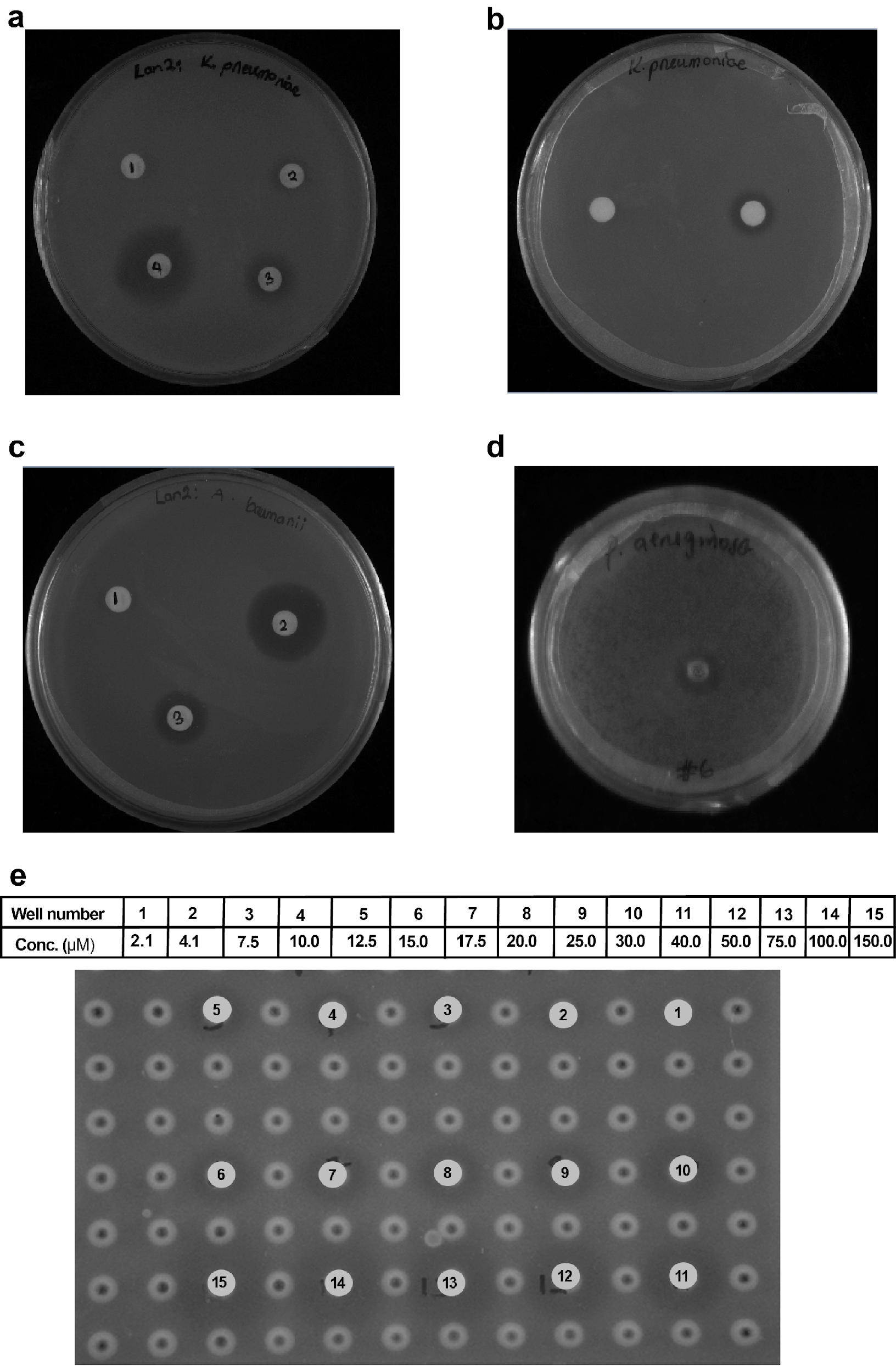
**

**Supplementary Fig. 50.** **Bioactivity screen of HPLC-purified modified core peptides**. **a)** Bioactivity screen of LanII-2A against *K. pneumoniae*. (1) Negative control (5% DMSO), (2) 4.1 µM LanII-2A, (3) 8.5 µM, and (4) 43 µM of LanII-2A dissolved in 5% DMSO. **b)** Bioactivity screen of LanII against *K. pneumoniae* (1) Negative control (30% acetonitrile), (2) 126 µM LanII dissolved in 30% acetonitrile. **c)** Bioactivity of LanII-2B against *A. baumanii* (1) negative control (H_2_O), (2) positive control (kanamycin), (3) 130 µM of LanII-2B. **d)** Bioactivity of LanII-2B against *P. aeruginosa* (130 µM). **e)** Representative example of a 96-well plate used for MIC determination of LanII-2A. MIC values were determined by dilutions in triplicate.

**
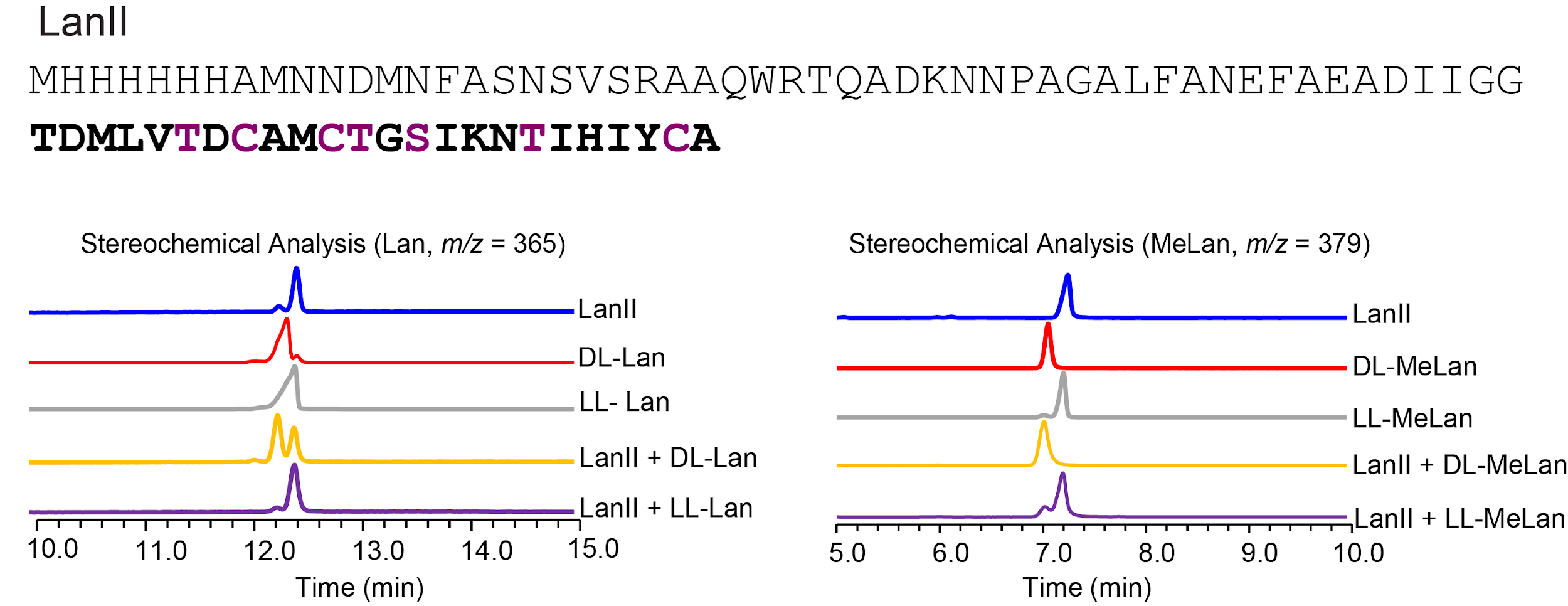
**

**Supplementary Fig. 51. Stereochemical analysis of LanII.** GC/MS traces for co-injections of synthetic, derivatized Lan standards and hydrolyzed/derivatized Lan residues obtained from LanII (selected ion monitoring, SIM, at 365 Da for Lan; 379 for MeLan).


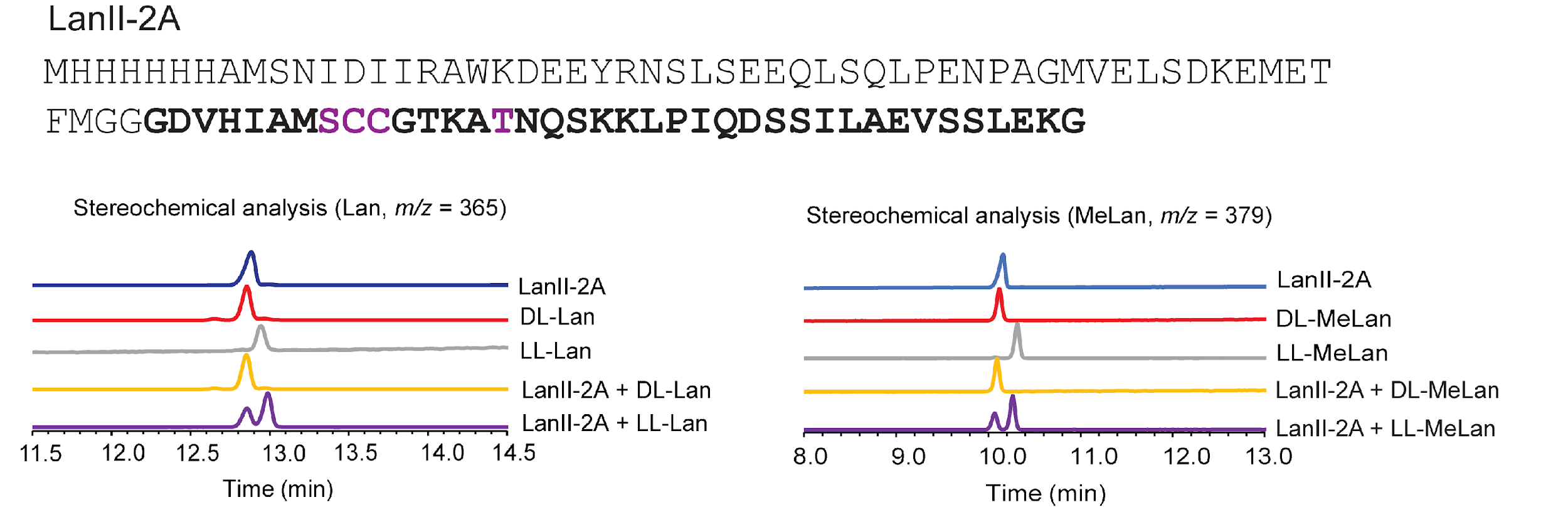


**Supplementary Fig. 52. Stereochemical analysis of LanII-2A.** GC/MS traces for co-injections of synthetic, derivatized Lan standards and hydrolyzed/derivatized Lan residues obtained from LanII-2A product (selected ion monitoring, SIM, at 365 Da for Lan; 379 for MeLan).

**
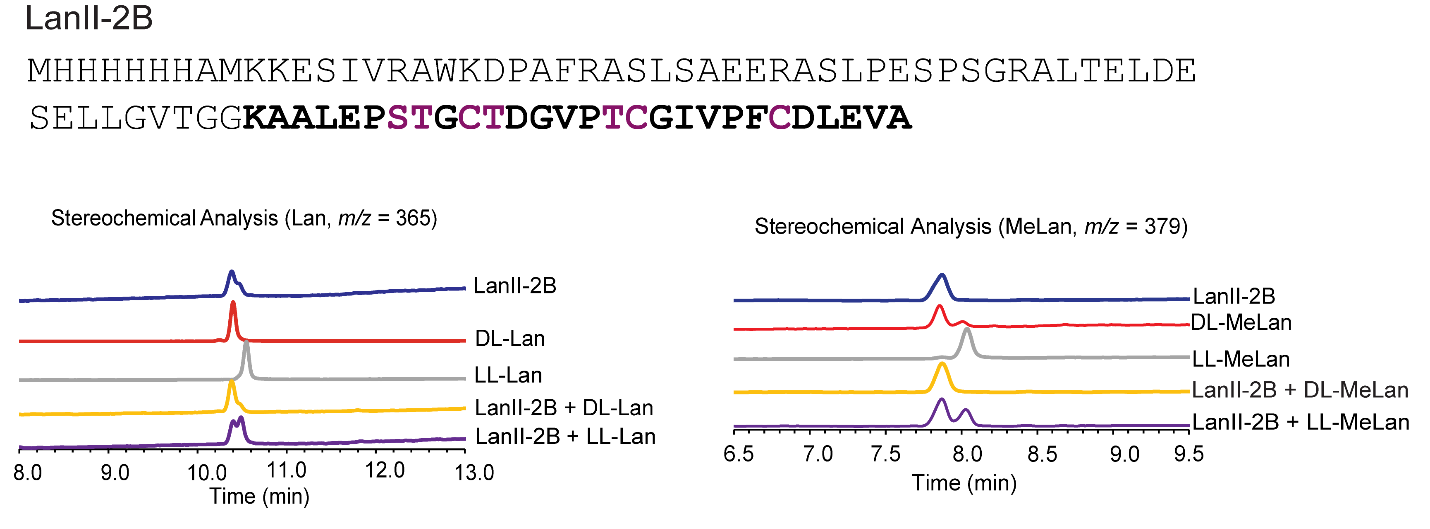
**

**Supplementary Fig. 53. Stereochemical analysis of LanII-2B.** GC/MS traces for co-injections of synthetic, derivatized Lan standards and hydrolyzed/derivatized Lan residues obtained from LanII-2B product (selected ion monitoring, SIM, at 365 Da for Lan; 379 for MeLan).


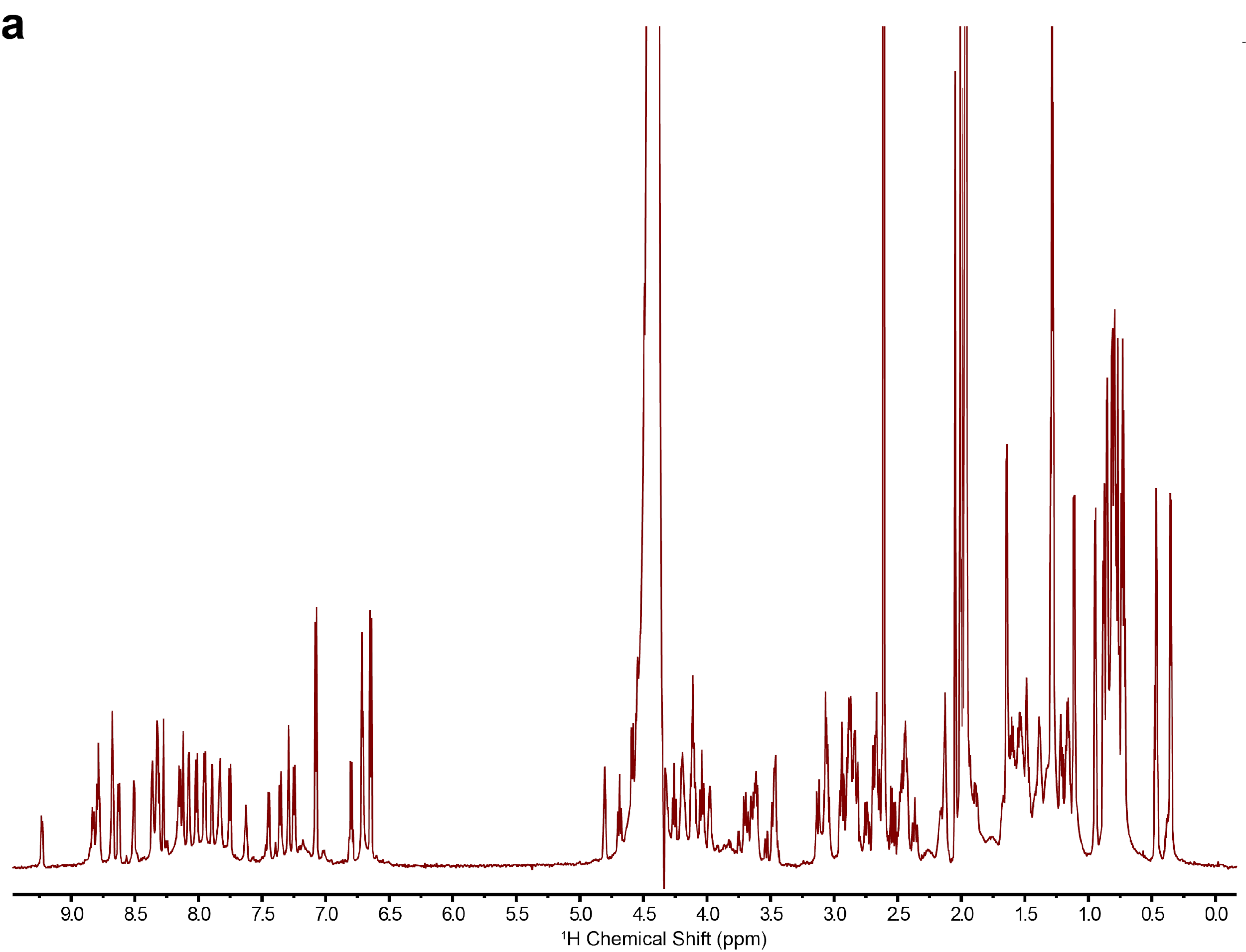


**
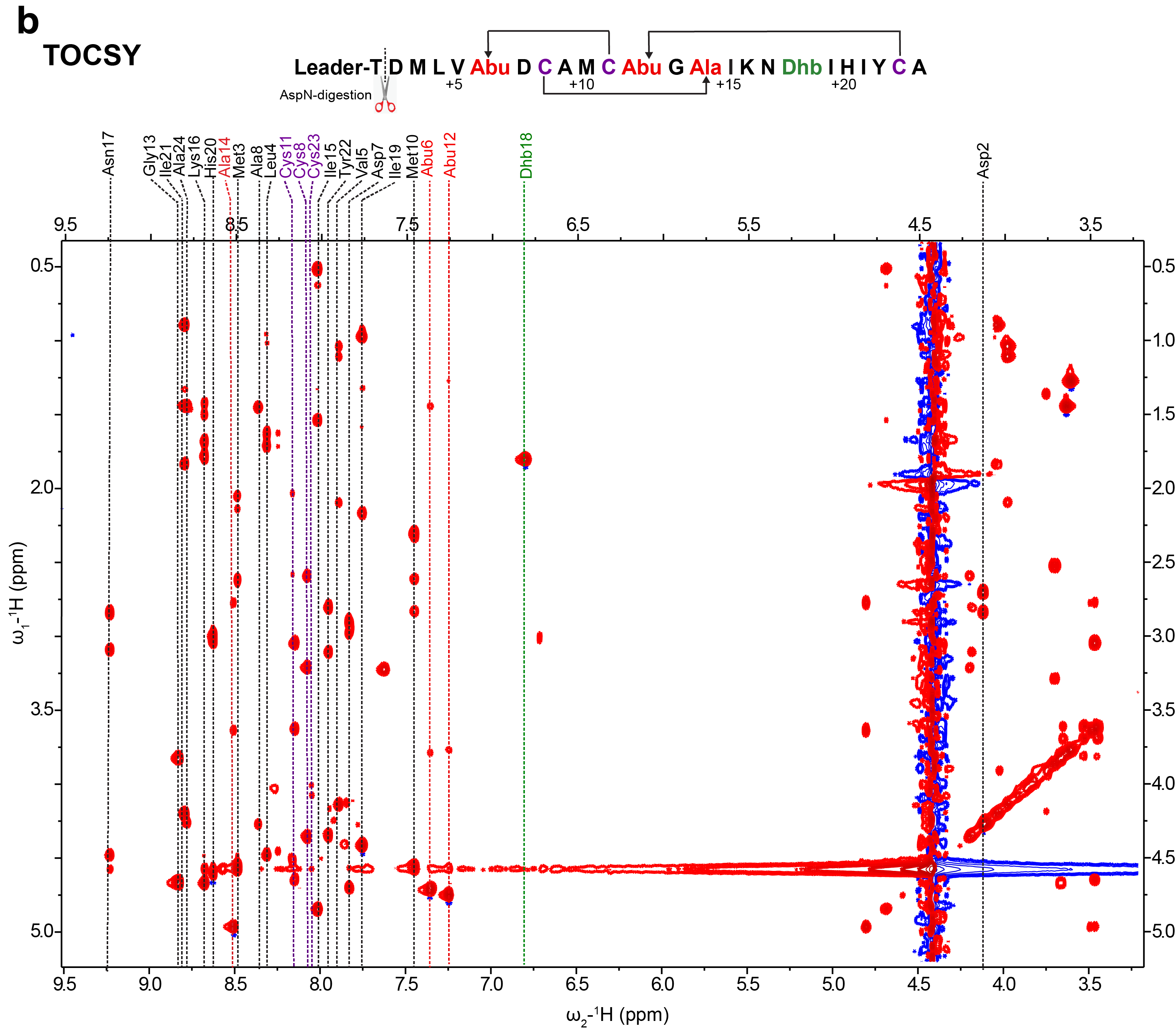
**


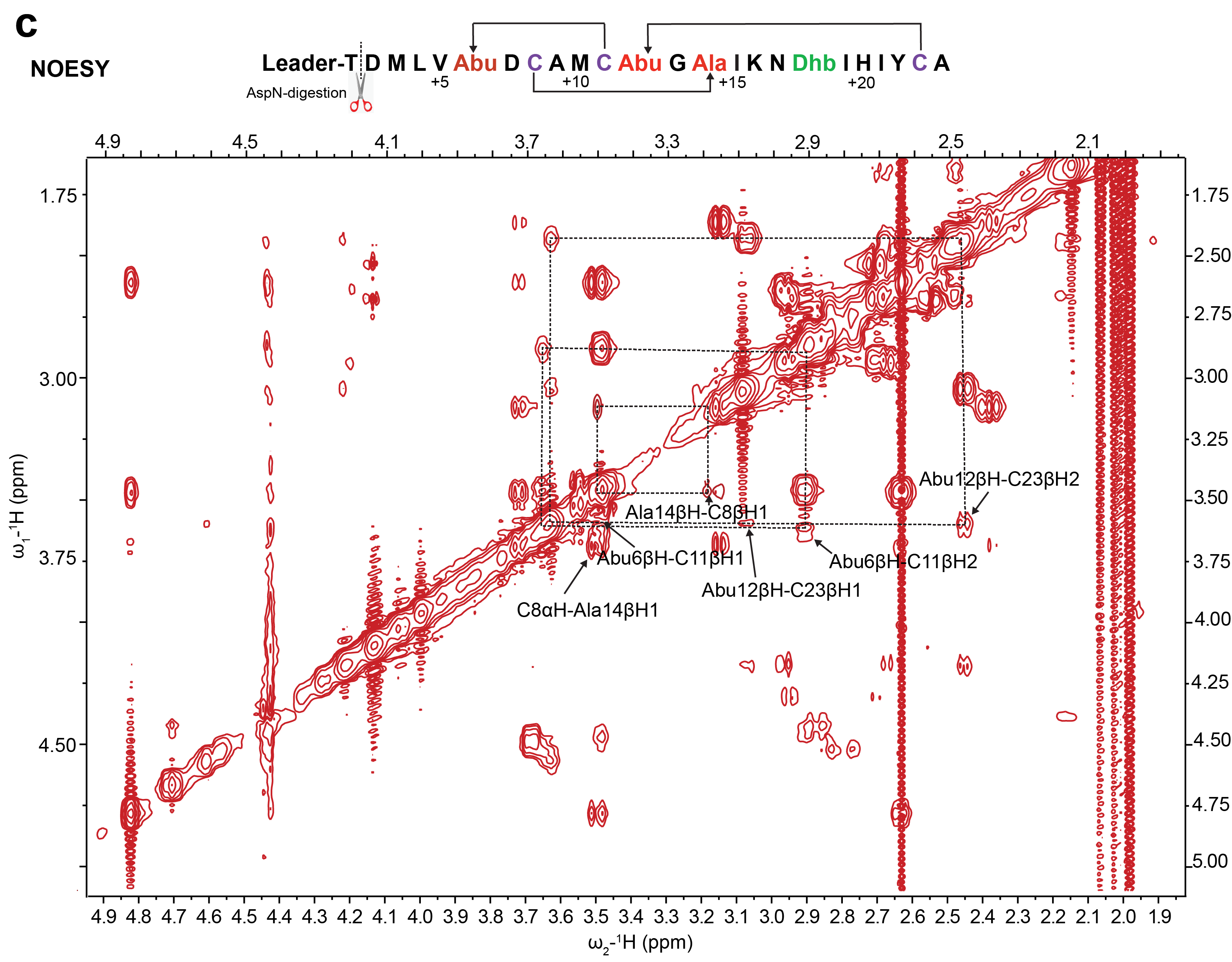


**Supplementary Fig. 54. Homonuclear NMR analysis of AspN-digested LanII. a)** A water-suppressed ^1^H NMR spectrum. **b)** A water-suppressed TOCSY NMR spectrum used to assign each amino acid. Each vertical line indicates a spin system corresponding to the amino acid shown at the top. **c)** Section of a water-suppressed NOESY spectrum showing the correlations of α protons with α and β protons across the thioether bridges in AspN-digested cyclized core peptide. Residues and lanthionine ring systems are indicated with black arrows. Sample was dissolved in 600 µL of 30% CD_3_CN and 70% D_2_O and spectra were acquired using a Varian INOVA 750 MHz spectrometer.


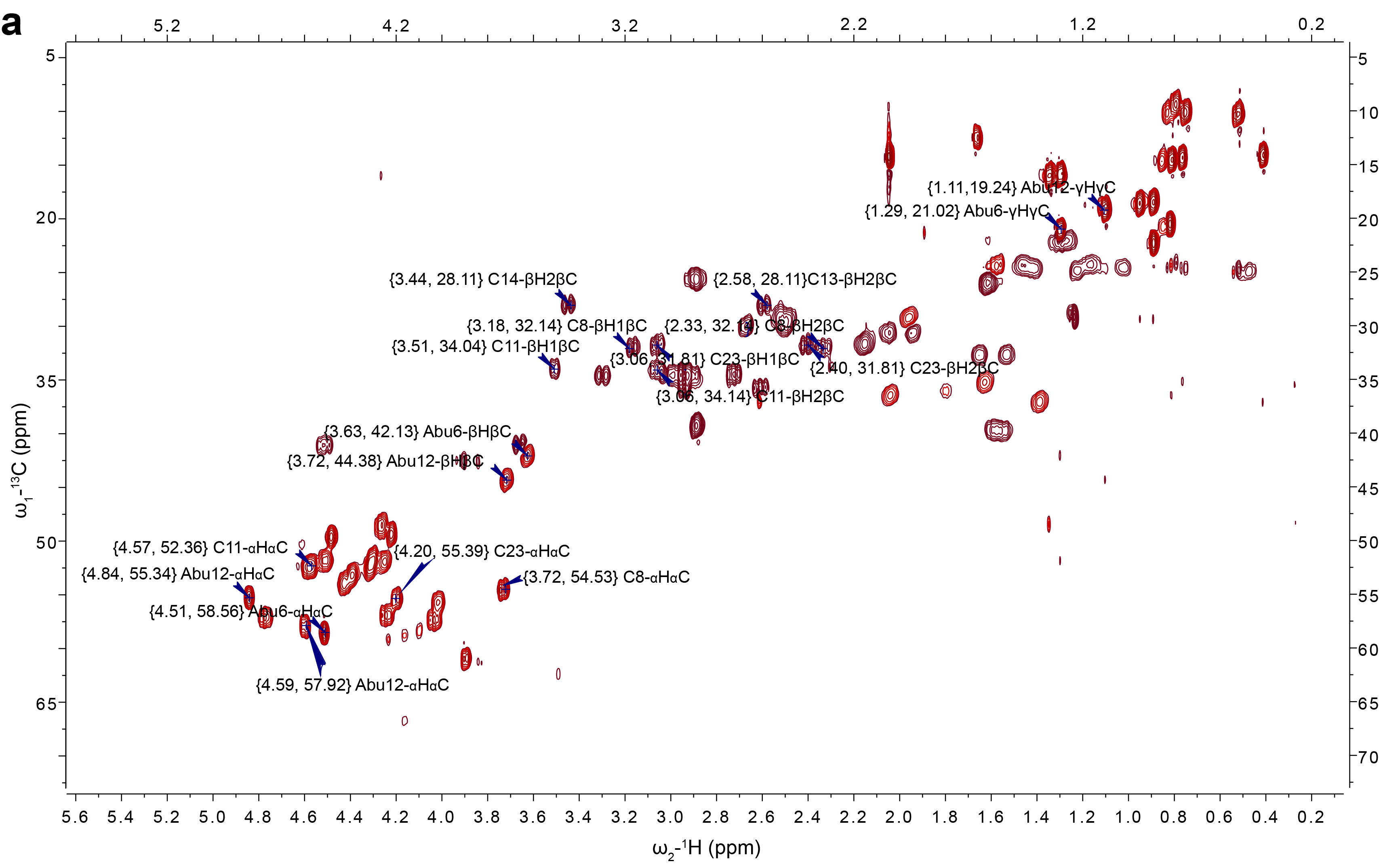


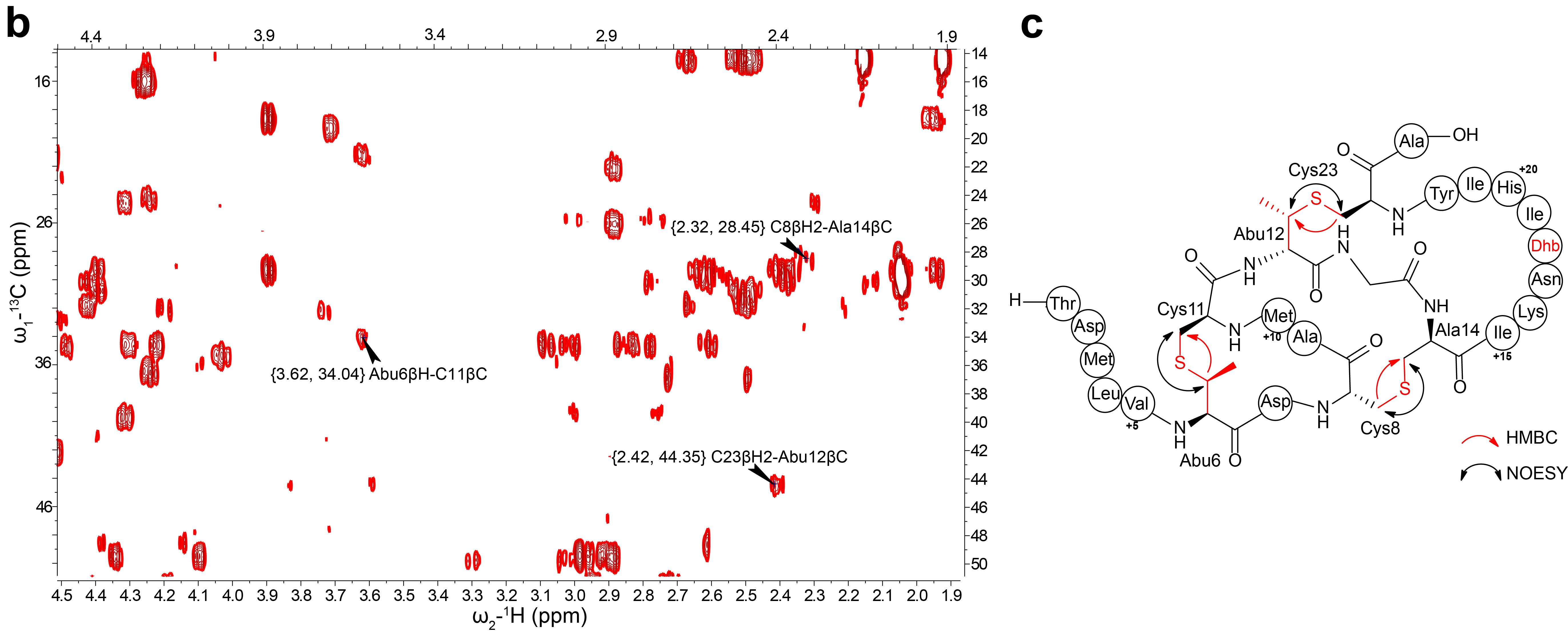


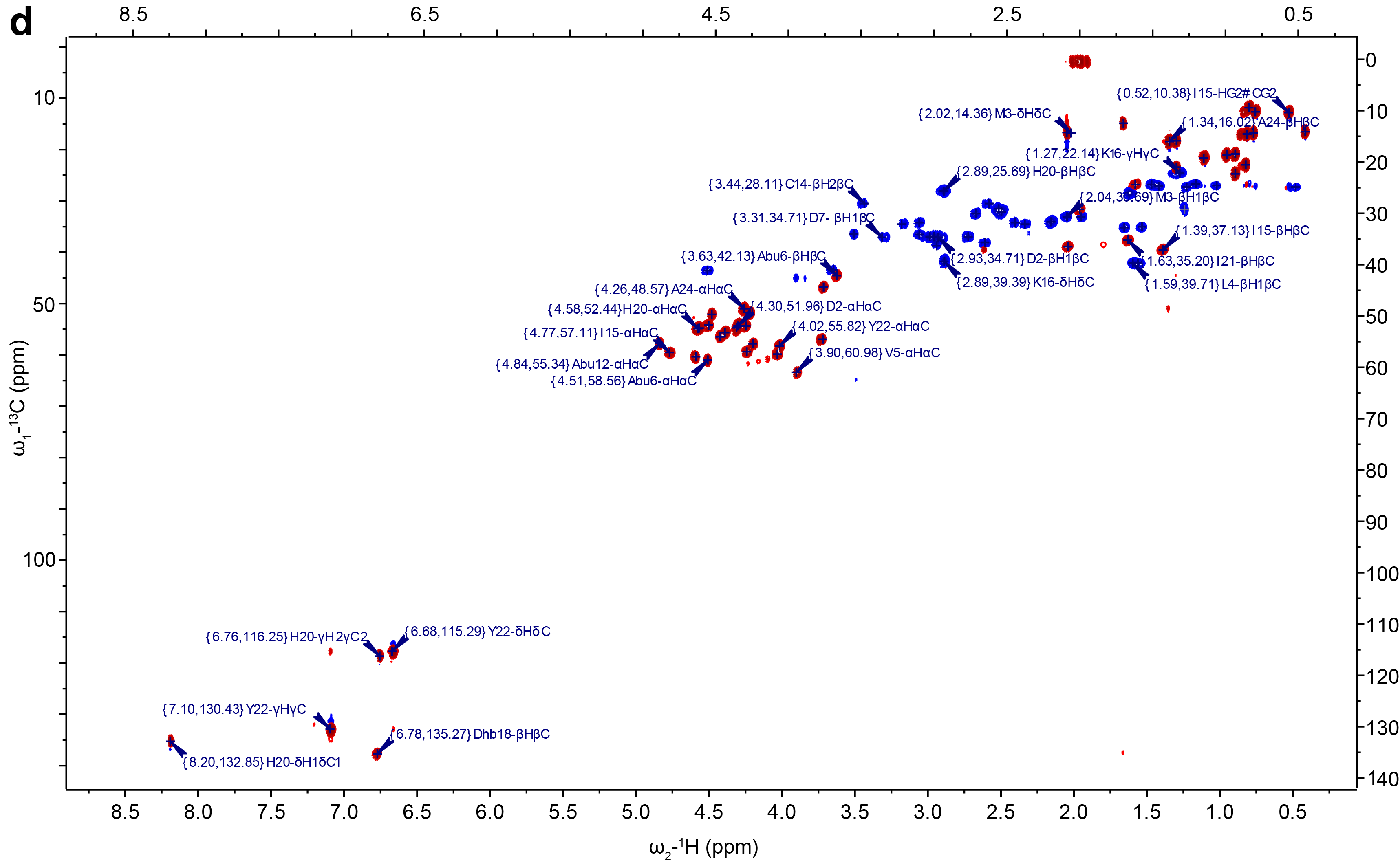


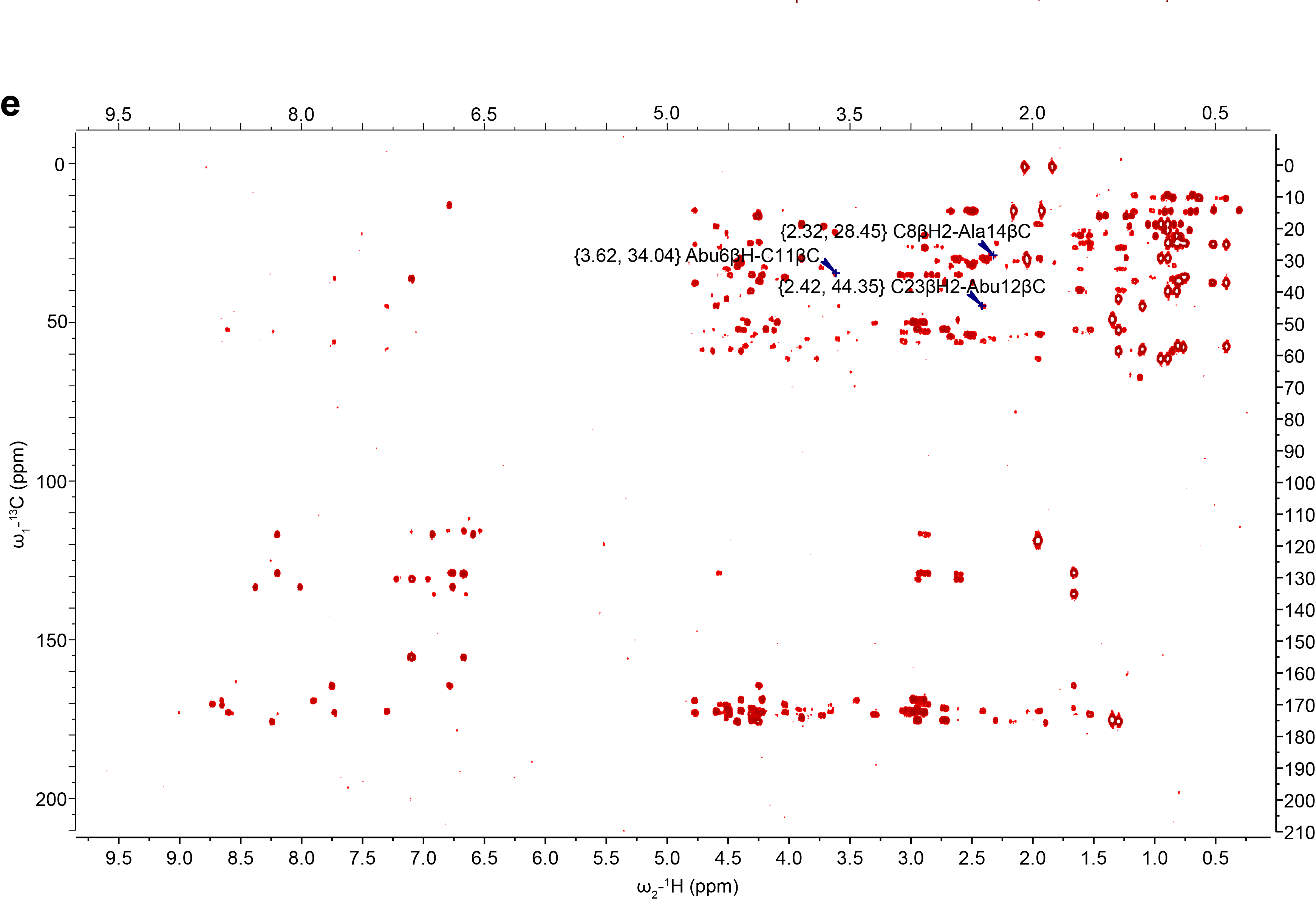


**Supplementary Fig. 55. Heteronuclear NMR analysis of AspN-digested LanII.**  **a)** A section of HSQC NMR spectrum showing ^1^H-^13^C crosspeaks for all residues involved in lanthionine linkages. **b)** A section of HMBC spectrum showing crosspeaks for multiple bond correlations of β protons and carbons across the thioether bridges in AspN-digested cyclized core peptide. **c)** Structure of LanII with arrows showing observed NOE and HMBC correlations. **d)** HSQC NMR spectrum showing ^1^H-^13^C crosspeaks for all residues in LanII core peptide that are not involved in thioether bridges. **e)** Full HMBC spectrum of AspN-digested cyclized core peptide with relevant peaks labeled. Sample was dissolved in 600 µL of 70% CD_3_CN and 30% D_2_O and spectra were acquired using Bruker Avance NEO 600 MHz spectrometer with the prodigy BBO probe.

**
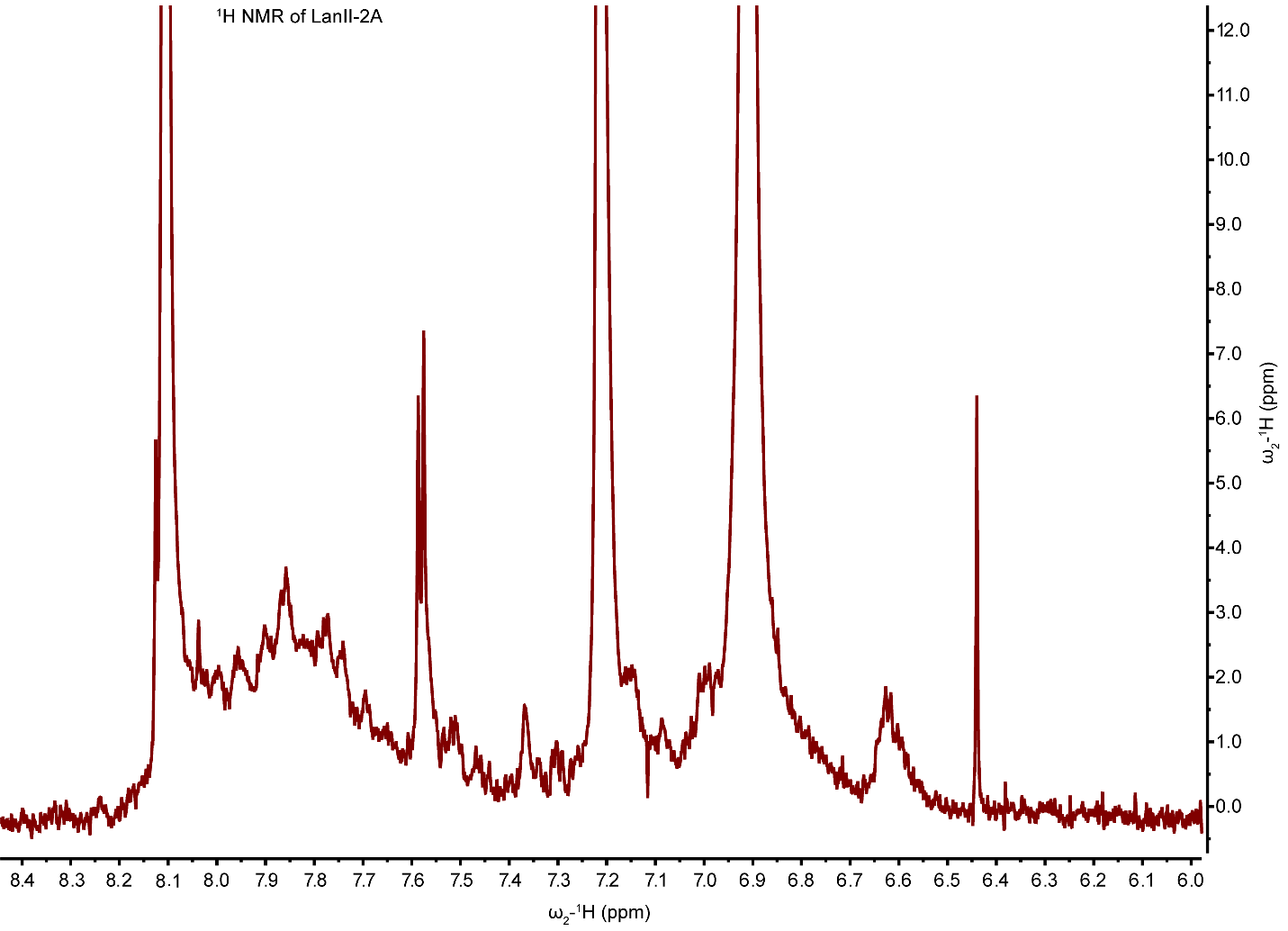
****Supplementary Fig. 56.** Representative ^1^H NMR spectrum of LanII-2A core peptide dissolved in DMSO-*d6* at 55 °C. The spectrum shows severe peak broadening, and the sample was not suitable for TOCSY and NOESY NMR acquisition. The peptide was not soluble in other NMR compatible solvents.

**Leader peptide sequences:**

WP_017747623.1 MSNIDIIRAWKDEEYRNSLSEEQLSQLPENPAG--MVELSDKEMETFMGG

2111288A MSQEAIIRSWKDPFSRENSTQN--------PAGNPFSELKEAQMDKLVGA

Q65DC4.1 MSKKEMILSWKNPMYRTESSYH--------PAGNILKELQEEEQHSIAGG

**: :* :**: * . : . *** : **.: : ..: *.

**Core peptide sequences:**

WP_017747623.1 GDVHIAM**SCC**G**T**KATNQSKKLPIQDSSILAEVSSLEKG

2111288A GDMEAA**CT**F---**T**LPGGGGV**CT**L**T**SE**C**I**C**---------

Q65DC4.1 **T**I**T**L**STC**AIL**S**KPLGNNGYL**CT**V**T**KE**C**MPS**C**------

**Supplementary Figure 57. Multiple sequence alignment of the precursor peptides of LanII-2A (WP_017747623.1), mersacidin (2111288A)^16^, and lichenicidin (Q65DC4.1)^17,18^**. The top lines are the leader peptides that have sequence similarity, the bottom lines are the core peptides that are not similar. For mersacidin and lichenicidin the ring patterns are known and Cys and Ser/Thr residues that form thioether rings are colored accordingly (i.e., red Cys forms ring with red Ser; black bold residues are dehydrated). For LanII-2A, the ring pattern is not known but the alignment illustrates that the product will have a different ring pattern than both mersacidin and lichenicidin. Residues that are post-translationally modified are in bold font. The Glu residue that is believed to be involved in lipid II binding^19,20^ is underlined in the sequences of mersacidin and lichenicidin. No corresponding Glu residue is found in LanII-2A.

**a**

WP_043693394.1 MNNDMNFASNSVSRAAQWR----TQADKNNPAGALFANE-FAEADII----GG**TDMLVTD**

WP_247374243.1 MSNEMIHAAKSVSRATQWR----HQAGSDNPAGELFANG-FAEADIV----GG**TEMLVTG**

WP_036114062.1 MNNDMNFASNSVSRAAQWR----TQADKNNPAGALFANE-FAEADIV----GG**TDMLVTD**

WP_166946955.1 MSNEMGFSSKSVSRAAEWR----KQAGNGNPAGDLFANA-FAEADIV----GG**TEIAVTD**

KAF1008451.1 MSNEMGYSSKSVSRAAEWR----KQAGNGNPAGDLFANA-FAEADIV----GG**TDIAVTD**

WP_247374242.1 MNIETIQASNPASRAALWR----SQAGNGNPAGELFAAD-FAEADIV----GG**TDIIVTN**

KAF1008452.1 MIVESMRASQSSARAAFWR----SNASAESPAGALFASE-FAEADIV----GG**TDIVVTD**

WP_166946956.1 MIVESMRASQPARRAAFWR----SNACADSPAGALFANE-FAEADIV----GG**TDIVVTD**

WP_250877592.1 MIVESMRASQPAGRAAFWR----SNACADSPAGALFANE-FAEADIV----GG**TDIVVTD**

WP_212819812.1 MST--LAPERADEIIESWLTGTAPVDGWENPAGPLFNSGRYAEYDITATGGGG**GITTHTG**

* . * .*** ** :** ** ** ***.**


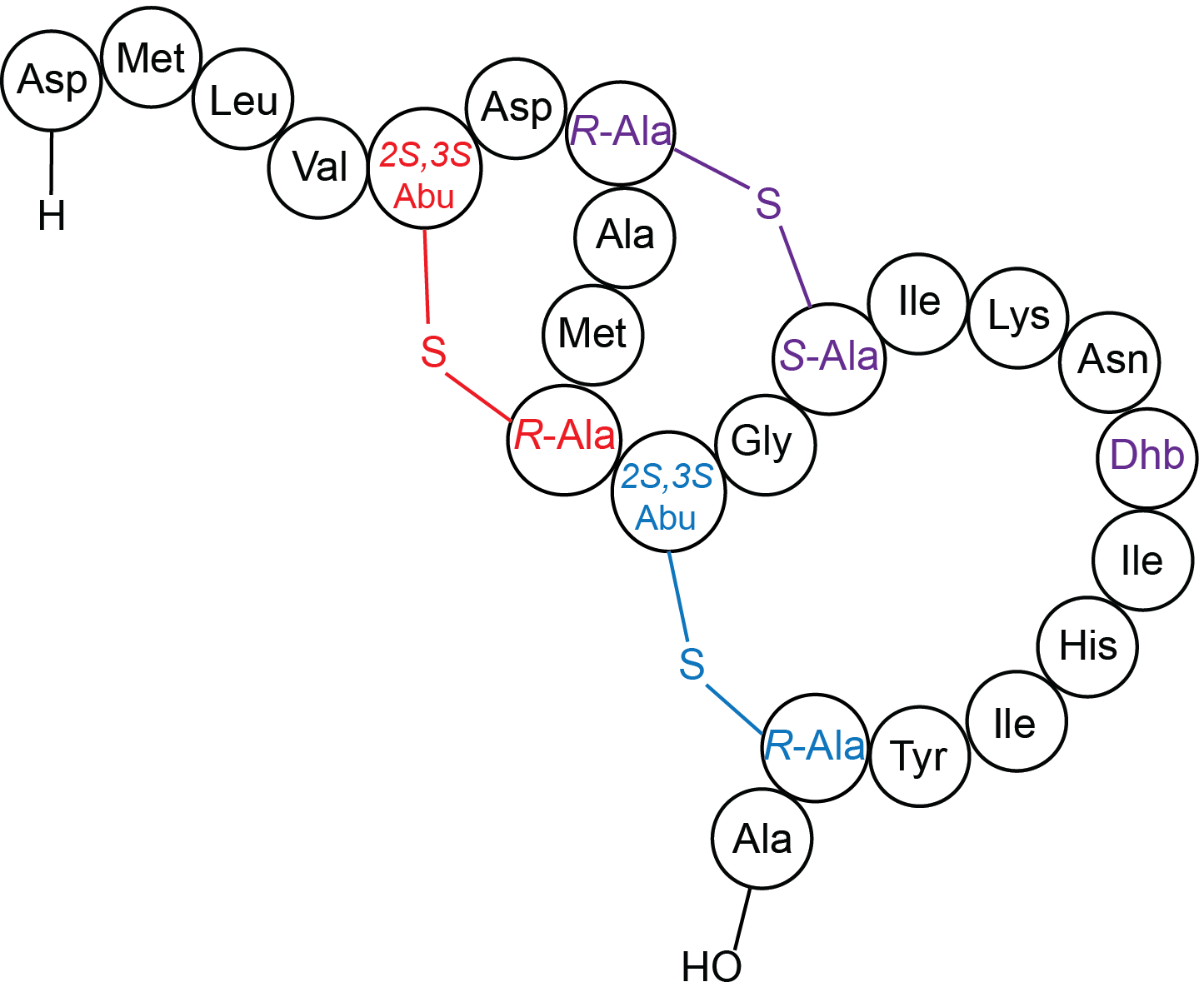


WP_043693394.1 **CAMCTGSIKN-TIHIYCA-**

WP_247374243.1 **CAMCTGSIEH-TIRIACA-**

WP_036114062.1 **CAMCTGSIKN-TIHIYCA-**

A

WP_166946955.1 **CAMCTGSIKT-TKITQCA-**

B

KAF1008451.1 **CATCTGSIKT-TRIIQCA-**

WP_247374242.1 **CVLCTGSVDANTRHVQCGA**

KAF1008452.1 **CLMCTGSIDTRTRHIQCGA**

C

WP_166946956.1 **CVMCTGSIDARTRHIQCGA**

WP_250877592.1 **CVMCTGSIDARTRHIQCGA**

WP_212819812.1 **CGPCTGSYPIE-----CY-**

*** ******

**b**

WP_043693394.1 ..PAGALFA-NEFAEADIIGG**T**DMLV**T_A_**D**C_B_**AM**C_A_T_C_**G**S_B_**IKNTIHIY**C_C_**A

WP_036102482.1 ..PAGPLFIGGEYVESDIIGEVAPAI**T_A_**G**C_B_S**L**C_A_T_C_**G**S_B_**MRIN---**CC_C_**A

**Supplementary Fig. 58. Sequence alignment of precursor peptides from the DUF6229 family. a)** Dehydratable and cyclizable residues in the core sequence of the precursor peptides are highlighted in purple. All entries are from Rhodobacteraceae except WP_212819812.1, which is from *Polymorphospora rubra* and expected to encode a truncated C ring (provided the ring pattern is retained). **b)** All other sequences in DUF6229 (221 total as of June 2022) contain four instead of three Cys and end in CysCysXxx (with Xxx usually an Ala or no amino acid). [WP_036102482.1](https://www.ncbi.nlm.nih.gov/protein/WP_036102482.1/) is an example and is the sequence encoded near the lysobactin BGC from *Lysobacter* ATCC 53042. This sequence encodes a peptide that is quite different from that of *Dyella* sp. 333MFSha (LanII BGC) reported in our study. Given the positions of the Ser/Thr and Cys residues, these lanthipeptide precursors likely result in the same A and B rings (shown in red and magenta with subscripts to indicate the residues linked by thioether bonds), but a C-ring that is likely much shorter and one additional ring, tentatively by the residues in orange. These peptides are found in many phyla including Actinomycetota and Pseudomonodota. Thus, DUF6229 represents at least two different lanthipeptide groups with different ring patterns.

# Supplementary Tables

**Supplementary Table 1.** Expression condition for the 27 BGCs that resulted in new products. LanI, class I lanthipeptide; LanII, class II lanthipeptide; LanIII, class III lanthipeptide; LanIV, class IV lanthipeptide; Gra, graspetide; Glk, glycocin; Las, lasso peptide; Lap, linear azol(in)e-containing peptide; Tha, thioamitide.

| **FAST-RiPPs identifier** | **Phylum** | **Bacterial Strain** | **Expression condition*** |
| --- | --- | --- | --- |
| LanI-101 | Bacteroidota | *Runella limosa* | 1 |
| LanII | Pseudomonadota | *Dyella* sp. 333MFSha | 1 |
| LanII-2A | Cyanobacteriota | *Scytonema hofmannii* | 1 |
| LanII-2B | Pseudomonadota | *Archangium violaceum* | 1 |
| LanII-2C | Cyanobacteriota | *Coleofasciculus chthonoplastes* | 1 |
| LanII-56 | Actinomycetota | *Streptomyces* sp*.* NRRL S-350 | 4 |
| LanII-23 | Pseudomonadota | *Pseudomonas ogarae* | 1 |
| LanII-2D | Actinomycetota | *Streptomyces* sp*.* ADI96-02 | 1 |
| LanII-2E | Cyanobacteriota | *Desertifilum* sp*.* IPPAS B-1220 | 3 |
| LanII-57 | Actinomycetota | *Kitasatospora xanthocidica* | 1 |
| LanIII | Pseudomonadota | *Myxococcus fulvus* | 1 |
| LanIII-6 | Bacillota | *Bacillus amyloliquefaciens* | 1 |
| LanIII-7 | Bacillota | *Bacillus cereus* | 1 |
| LanIV | Pseudomonadota | *Chondromyces crocatus* sp. Cm c5 | 1 |
| LanIV | Actinomycetota | *Streptomyces* sp*.* DvalAA-43 | 1 |
| Lap-1 | Bacillota | *Enterococcus caccae* | 1 |
| Glk-1 | Bacillota | *Bacillus cereus* | 1 |
| Glk-2 | Bacillota | *Enterococcus faecalis* ATCC 6055 | 1 |
| Glk-3 | Bacillota | *Dellaglioa algida* | 1 |
| Las-2 | Bacillota | *Bacillus cereus* | 4 |
| Las-6 | Actinomycetota | *Streptacidiphilus melanogenes* | 1 |
| Gra-3 | Bacillota | *Bacillus* sp*.* S66 | 4 |
| Gra-4 | Pseudomonadota | *Burkholderia seminalis* | 4 |
| Gra-5 | Pseudomonadota | *Glaciecola* sp. KUL10 | 1 |
| Gra-7 | Pseudomonadota | *Legionella pneumophila* | 4 |
| Gra-8 | Pseudomonadota | *Lysobacter capsici* | 1 |
| Tha-1 | Pseudomonadota | *Desulforegula conservatrix* | 2 |
| * 4 expression conditions are described in Methods | | | |

**Supplemental Table 2.** ^1^H chemical shifts assignments for LanII core peptide dissolved in 70% H_2_O/30% CD_3_CN at 25^o^C. **(2)DMLVAbuDCAMCAbuGD-AlaIKNDhbIHIYCA(24)**.

| Residue number | Amino acid | NH | αH | βH | γH | δH | others |
| --- | --- | --- | --- | --- | --- | --- | --- |
| 2 | D |  | 4.12 | 2.67, 2.55 |  |  |  |
| 3 | M | 8.48  (br) | 4.40 | 1.99, 1.91 | 2.46 | CH_3_: 1.99 |  |
| 4 | L | 8.31 | 4.32 | 1.55 | 1.468 | 0.85,  0.80 |  |
| 5 | V | 7.89 | 3.98 | 1.93 | 0.95,  0.88 |  |  |
| 6 | Abu | 7.36 | 4.55 | 3.63 | 1.29 |  |  |
| 7 | D | 7.83 | 4.54 | 2.82, 2.74 |  | | |
| 8 | C | 8.12 | 3.70 | 3.13, 2.36 |  |  |  |
| 9 | A | 8.36 | 4.11 | 1.29 |  |  |  |
| 10 | M | 7.45 | 4.40 | 2.45, 2.15 | 2.67 | CH_3_: 2.05 |  |
| 11 | C | 8.15 | 4.48 | 3.46, 2.88 |  |  |  |
| 12 | Abu | 7.25 | 4.57 | 3.611 | 1.126 |  |  |
| 13 | G | 8.83 | 4.51,  3.67 |  |  |  |  |
| 14 | Ala | 8.51 | 4.81 | 3.48, 2.61 |  |  |  |
| 15 | I | 8.02 | 4.69 | 1.38 | HG1#:  1.16 | HG2#:  0.47 | HD#:  0.354 |
| 16 | K | 8.68 | 4.51 | 1.63, 1.53 | 1.34, 1.27 | | |
| 17 | N | 9.23 | 4.32 | 2.93, 2.69 | 7.29, 6.71 |  | |
| 18 | Dhb | 8.68 |  | 6.80 | 1.64 |  | |
| 19 | I | 7.75 | 4.26 | 2.01 | HG1#:  1.42 | HG2#:  0.77 | HD#:  0.82 |
| 20 | H | 8.63 | 4.45 | 2.88, 2.83 | HD2:6.71, HE1:8.27 | | |
| 21 | I | 8.79 | 4.04 | 1.673 | HG1#: 1.30 | HG2#:  0.73 | HD#:  0.73 |
| 22 | Y | 7.95 | 4.18 | 2.94, 2.64 | HD#:7.08, HE#: 6.65 | | |
| 23 | C | 8.08 | 4.20 | 3.05, 2.43 |  |  |  |
| 24 | A | 8.78 | 4.26 | 1.45 |  |  |  |

Crosslinks: Abu6-Cys11, Cys8-Ala14, Abu12-Cys23

**Supplemental Table 3.** ^1^H-^13^C chemical shifts assignments for the Asp-N digested modified LanII peptide dissolved in 70% CD_3_CN /30% D_2_O at 25^o^C. The sequence is: **(2)DMLVAbuDCAMCAbuGD-AlaIKNDhbIHIYCA(24)**. The CD_3_CN-CH3 peak at 1.96 ppm was used as ^1^H reference and 0.50 ppm as ^13^C reference. Both ^1^H and ^13^C chemical shifts are in red for residues involved in thioether bridges and all others are in blue.

| Residue number | AA | NH | αH/C | βH/C | γH/C | δH/C | | others |
| --- | --- | --- | --- | --- | --- | --- | --- | --- |
| 2 | D |  | 4.30  51.8 | 2.92, 2.72  34.7 |  |  | |  |
| 3 | M |  | 4.39  53.2 | 2.05, 1.94  30.7 | 2.51  29.4 | 2.045 14.3 | |  |
| 4 | L |  | 4.32  52.3 | 1.59, 1.56  39.7 | 1.57,  24.4 | 0.89, 22.3  0.82, 20.5 | | |
| 5 | V |  | 3.90  61.0 | 1.96  29.3 | 0.95,18.7  0.89,18.5 |  | |  |
| 6 | Abu | 7.18 | 4.50  58.7 | 3.62  42.2 | 1.30, 20.9 |  | |  |
| 7 | D |  | 4.48  49.7 | 3.30, 3.02  34.7 |  | | | |
| 8 | C |  | 3.73  54.6 | 3.17, 2.34  32.0 |  |  | |  |
| 9 | A |  | 4.25  52.1 | 1.30  15.8 |  |  | |  |
| 10 | M | 7.60 | 4.42  54.0 | 2.16  31.7 | 2.67, 2.51  30.1 | 2.05 14.3 | |  |
| 11 | C | 8.24 | 4.58  52.6 | 3.52, 3.07  34.1 |  |  | |  |
| 12 | Abu | 7.30 | 4.60  58.1 | 3.72  44.6 | 1.10, 19.1 |  | |  |
| 13 | G | 8.99 | 4.52  3.66  41.1 |  |  |  | |  |
| 14 | Ala | 8.65 | 4.85  55.3 | 3.45, 2.59  28.2 |  |  | |  |
| 15 | I | 7.90 | 4.77  57.1 | 1.39  37.1 | HG1#:  1.22, 0.48  25.0 | HG2#:  0.52  10.3 | | HD#:  0.41, 14.1 |
| 16 | K | 8.60 | 4.51  51.8 | 1.65, 1.53  32.7 | 1.27, 22.1 | | 2.89  39.4 | |
| 17 | N |  | 4.22  49.5 | 2.96, 2.93  34.6 |  |  | | |
| 18 | Dhb |  |  | 6.78  135.3 | 1.66  12.5 |  | | |
| 19 | I | 7.76 | 4.23  57.0 | 2.04  36.4 | HG1#:  1.46, 1.16  24.3 | HG2#:  0.79  9.53 | | HD#:  0.81,14.6 |
| 20 | H | 8.58 | 4.57  52.4 | 2.89  25.7 | 6.76, 112.2; 8.20, 132.8 | | | |
| 21 | I | 8.72 | 4.03  57.4 | 1.61  35.4 | 1.41, 1.02  24.6 | 0.75  10.2 | | 0.76  14.4 |
| 22 | Y | 7.73 | 4.01  55.7 | 2.94, 2.61  35.7 | 7.10, 130.5; 6.67, 115.3 | | | |
| 23 | C |  | 4.20  55.4 | 3.06, 2.42  31.8 |  |  | |  |
| 24 | A |  | 4.26  48.6 | 1.35  16.0 |  |  | |  |

Crosslinks: Abu6-Cys11, Cys8-Ala14, Abu12-Cys23.

# Supplementary References

1. Ren H, Biswas S, Ho S, van der Donk WA, Zhao H. Rapid discovery of glycocins through pathway refactoring in *Escherichia coli*. *ACS Chem Biol* **13**, 2966-2972 (2018).

2. Ramesh S*, et al.* Bioinformatics-Guided Expansion and Discovery of Graspetides. *ACS Chem Biol* **16**, 2787-2797 (2021).

3. Walker MC, Eslami SM, Hetrick KJ, Ackenhusen SE, Mitchell DA, van der Donk WA. Precursor peptide-targeted mining of more than one hundred thousand genomes expands the lanthipeptide natural product family. *BMC Genomics* **21**, 387 (2020).

4. Zhang Y*, et al.* Biosynthesis of Gut-Microbiota-Derived Lantibiotics Reveals a Subgroup of S8 Family Proteases for Class III Leader Removal. *Angew Chem Int Ed Engl* **61**, e202114414 (2022).

5. DiCaprio AJ, Firouzbakht A, Hudson GA, Mitchell DA. Enzymatic reconstitution and biosynthetic investigation of the lasso peptide fusilassin. *J Am Chem Soc* **141**, 290-297 (2019).

6. Montalbán-López M*, et al.* New developments in RiPP discovery, enzymology and engineering. *Nat Prod Rep* **138**, 130 - 239 (2021).

7. Georgiou MA, Dommaraju SR, Guo X, Mast DH, Mitchell DA. Bioinformatic and Reactivity-Based Discovery of Linaridins. *ACS Chem Biol* **15**, 2976-2985 (2020).

8. Cox CL, Doroghazi JR, Mitchell DA. The genomic landscape of ribosomal peptides containing thiazole and oxazole heterocycles. *BMC Genomics* **16**, 778 (2015).

9. Hudson GA*, et al.* Bioinformatic mapping of radical S-adenosylmethionine-dependent ribosomally synthesized and post-translationally modified peptides identifies new Cα, Cβ, and Cγ-linked thioether-containing peptides. *J Am Chem Soc* **141**, 8228-8238 (2019).

10. Liu A*, et al.* Functional elucidation of TfuA in peptide backbone thioamidation. *Nat Chem Biol* **17**, 585-592 (2021).

11. Mahanta N, Liu A, Dong S, Nair SK, Mitchell DA. Enzymatic reconstitution of ribosomal peptide backbone thioamidation. *Proc Natl Acad Sci U S A* **115**, 3030-3035 (2018).

12. Haft DH, Basu MK, Mitchell DA. Expansion of ribosomally produced natural products: a nitrile hydratase- and Nif11-related precursor family. *BMC Biol* **8**, 70 (2010).

13. Schwalen CJ, Hudson GA, Kille B, Mitchell DA. Bioinformatic expansion and discovery of thiopeptide antibiotics. *J Am Chem Soc* **140**, 9494-9501 (2018).

14. Hou J, Robbel L, Marahiel MA. Identification and characterization of the lysobactin biosynthetic gene cluster reveals mechanistic insights into an unusual termination module architecture. *Chem Biol* **18**, 655-664 (2011).

15. Enghiad B*, et al.* PlasmidMaker: a versatile, automated, and high Throughput end-to-end platform for plasmid construction. *Nat Commun* **13**, 2697 (2022).

16. Bierbaum G, Brötz H, Koller KP, Sahl HG. Cloning, sequencing and production of the lantibiotic mersacidin. *FEMS Microbiol Lett* **127**, 121-126 (1995).

17. Caetano T, Krawczyk JM, Mosker E, Süssmuth RD, Mendo S. Heterologous expression, biosynthesis, and mutagenesis of type II lantibiotics from *Bacillus licheniformis* in *Escherichia coli*. *Chem Biol* **18**, 90-100 (2011).

18. Shenkarev ZO*, et al.* Isolation, structure elucidation, and synergistic antibacterial activity of a novel two-component lantibiotic lichenicidin from *Bacillus licheniformis* VK21. *Biochemistry* **49**, 6462-6472 (2010).

19. Brötz H, Bierbaum G, Leopold K, Reynolds PE, Sahl HG. The lantibiotic mersacidin inhibits peptidoglycan synthesis by targeting lipid II. *Antimicrob Agents Chemother* **42**, 154-160 (1998).

20. Szekat C, Jack RW, Skutlarek D, Farber H, Bierbaum G. Construction of an expression system for site-directed mutagenesis of the lantibiotic mersacidin. *Appl Environ Microbiol* **69**, 3777-3783 (2003).
