# Supplementary material for: A scalable platform to discover antimicrobials of ribosomal origin
Source: Nat Commun. 2022 Oct 17;13:6135. doi: 10.1038/s41467-022-33890-w (PMC9576775; doi:10.1038/s41467-022-33890-w)
Supplement: Supplementary file 3 — Description of Additional Supplementary Files [file 41467_2022_33890_MOESM3_ESM.pdf]

Supplementary Data 1: Accessions for each of the genes in each gene cluster used in this study, along with the amino acid sequence of the precursor peptides. The optimized nucleotide sequences that were synthesized for all the genes are also included.

Supplementary Data 2: DNA sequences of 96 plasmids in this study.
