# Supplementary material for: A scalable platform to discover antimicrobials of ribosomal origin
Source: Nat Commun. 2022 Oct 17;13:6135. doi: 10.1038/s41467-022-33890-w (PMC9576775; doi:10.1038/s41467-022-33890-w)
Supplement: Supplementary file 6 — Reporting Summary [file 41467_2022_33890_MOESM6_ESM.pdf]

nature portfolio

Corresponding author(s): Huimin Zhao, Douglas A. Mitchell, Wilfred A. van der Donk

Last updated by author(s): Sep 30, 2022

Reporting Summary

Nature Portfolio wishes to improve the reproducibility of the work that we publish. This form provides structure for consistency and transparency in reporting. For further information on Nature Portfolio policies, see our Editorial Policies and the Editorial Policy Checklist.

Statistics

For all statistical analyses, confirm that the following items are present in the figure legend, table legend, main text, or Methods section.

n/a	Confirmed	
[ ]	[x]	The exact sample size (*n*) for each experimental group/condition, given as a discrete number and unit of measurement
[ ]	[x]	A statement on whether measurements were taken from distinct samples or whether the same sample was measured repeatedly
[x]	[ ]	The statistical test(s) used AND whether they are one- or two-sided<br>*Only common tests should be described solely by name; describe more complex techniques in the Methods section.*
[x]	[ ]	A description of all covariates tested
[x]	[ ]	A description of any assumptions or corrections, such as tests of normality and adjustment for multiple comparisons
[ ]	[x]	A full description of the statistical parameters including central tendency (e.g. means) or other basic estimates (e.g. regression coefficient) AND variation (e.g. standard deviation) or associated estimates of uncertainty (e.g. confidence intervals)
[x]	[ ]	For null hypothesis testing, the test statistic (e.g. *F*, *t*, *r*) with confidence intervals, effect sizes, degrees of freedom and *P* value noted<br>*Give P values as exact values whenever suitable.*
[x]	[ ]	For Bayesian analysis, information on the choice of priors and Markov chain Monte Carlo settings
[x]	[ ]	For hierarchical and complex designs, identification of the appropriate level for tests and full reporting of outcomes
[x]	[ ]	Estimates of effect sizes (e.g. Cohen's *d*, Pearson's *r*), indicating how they were calculated

Our web collection on statistics for biologists contains articles on many of the points above.

Software and code

Policy information about availability of computer code

Data collection	Genome-mining tool RODEO version 2.3 (https://rodeo.scs.illinois.edu/) was used to identify 96 RiPP BGCs. NMR (nuclear magnetic resonance) data were collected using Topspin 4.1.4.
Data analysis	DNA sequences were analyzed by SnapGene viewer software (version 5.0). NMR (nuclear magnetic resonance) data were analyzed by MestReNova (version 11.0.3). Matrix-assisted laser desorption/ionization (MALDI) mass spectrometric data were analyzed using flexAnalysis (version 3.3). High-resolution electrospray ionization mass spectrometric (HRESIMS) data were analyzed by Thermo caliber Roadmap (version 2.2 SP1 build 48). Structures were drawn using ChemDraw professional (version 17.1.0.105(19)) and Adobe Illustrator (version 26.2.1)

For manuscripts utilizing custom algorithms or software that are central to the research but not yet described in published literature, software must be made available to editors and reviewers. We strongly encourage code deposition in a community repository (e.g. GitHub). See the Nature Portfolio guidelines for submitting code & software for further information.

Data

Policy information about availability of data

All manuscripts must include a data availability statement. This statement should provide the following information, where applicable:

- Accession codes, unique identifiers, or web links for publicly available datasets
- A description of any restrictions on data availability
- For clinical datasets or third party data, please ensure that the statement adheres to our policy

We declare that all the data supporting the findings of this study are available within the main text, Supplementary data 1, and Supplementary information

March 2021

documents. All plasmids and raw data are available upon request from the corresponding authors. Source data are provided with this paper. Gene sequences used in this study were obtained from NCBI: https://www.ncbi.nlm.nih.gov/. NCBI accessions for each gene can be found in Supplementary data 1.

Human research participants

Policy information about studies involving human research participants and Sex and Gender in Research.

Reporting on sex and gender	N/A
Population characteristics	N/A
Recruitment	N/A
Ethics oversight	N/A

Note that full information on the approval of the study protocol must also be provided in the manuscript.

Field-specific reporting

Please select the one below that is the best fit for your research. If you are not sure, read the appropriate sections before making your selection.

- [x] Life sciences
- [ ] Behavioural & social sciences
- [ ] Ecological, evolutionary & environmental sciences

For a reference copy of the document with all sections, see nature.com/documents/nr-reporting-summary-flat.pdf

Life sciences study design

All studies must disclose on these points even when the disclosure is negative.

Sample size	96. This was chosen to allow for easy use of 96 well plates.
Data exclusions	No data was excluded from the analysis.
Replication	Due to the large number of BGCs, each experiment was only performed once.
Randomization	No randomization was performed because these experiments did not involve assigning treatments to test groups.
Blinding	Blinding was not performed as no group allocation was performed in this study.

Reporting for specific materials, systems and methods

We require information from authors about some types of materials, experimental systems and methods used in many studies. Here, indicate whether each material, system or method listed is relevant to your study. If you are not sure if a list item applies to your research, read the appropriate section before selecting a response.

Materials & experimental systems

n/a	Involved in the study	
[x]	[ ]	Antibodies
[x]	[ ]	Eukaryotic cell lines
[x]	[ ]	Palaeontology and archaeology
[x]	[ ]	Animals and other organisms
[x]	[ ]	Clinical data
[x]	[ ]	Dual use research of concern

Methods

n/a	Involved in the study	
[x]	[ ]	ChIP-seq
[x]	[ ]	Flow cytometry
[x]	[ ]	MRI-based neuroimaging
